# Second-order group knockoffs with applications to genome-wide association studies

**DOI:** 10.1093/bioinformatics/btae580

**Published:** 2024-09-28

**Authors:** Benjamin B Chu, Jiaqi Gu, Zhaomeng Chen, Tim Morrison, Emmanuel Candès, Zihuai He, Chiara Sabatti

**Affiliations:** Department of Biomedical Data Science, Stanford University, Stanford, CA, 94305, USA; Department of Neurology and Neurological Sciences, Stanford University, Stanford, CA, 94035, USA; Department of Statistics, Stanford University, Stanford, CA, 94035, USA; Department of Statistics, Stanford University, Stanford, CA, 94035, USA; Department of Statistics, Stanford University, Stanford, CA, 94035, USA; Department of Mathematics, Stanford University, Stanford, CA, 94035, USA; Department of Biomedical Data Science, Stanford University, Stanford, CA, 94305, USA; Department of Neurology and Neurological Sciences, Stanford University, Stanford, CA, 94035, USA; Quantitative Sciences Unit, Department of Medicine, Stanford University, Stanford, CA, 94035, USA; Department of Biomedical Data Science, Stanford University, Stanford, CA, 94305, USA; Department of Statistics, Stanford University, Stanford, CA, 94035, USA

## Abstract

**Motivation:**

Conditional testing via the knockoff framework allows one to identify—among a large number of possible explanatory variables—those that carry unique information about an outcome of interest and also provides a false discovery rate guarantee on the selection. This approach is particularly well suited to the analysis of genome-wide association studies (GWAS), which have the goal of identifying genetic variants that influence traits of medical relevance.

**Results:**

While conditional testing can be both more powerful and precise than traditional GWAS analysis methods, its vanilla implementation encounters a difficulty common to all multivariate analysis methods: it is challenging to distinguish among multiple, highly correlated regressors. This impasse can be overcome by shifting the object of inference from single variables to groups of correlated variables. To achieve this, it is necessary to construct “group knockoffs.” While successful examples are already documented in the literature, this paper substantially expands the set of algorithms and software for group knockoffs. We focus in particular on second-order knockoffs, for which we describe correlation matrix approximations that are appropriate for GWAS data and that result in considerable computational savings. We illustrate the effectiveness of the proposed methods with simulations and with the analysis of albuminuria data from the UK Biobank.

**Availability and implementation:**

The described algorithms are implemented in an open-source Julia package Knockoffs.jl. R and Python wrappers are available as knockoffsr and knockoffspy packages.

## 1 Introduction

A common problem, in our data-rich era, is the selection of important features among a large number of recorded variables, with reproducibility guarantees. For example, in genome-wide association studies (GWAS), one observes data on millions of features, corresponding to polymorphic loci in the human genome, and wants to identify those that carry information about a phenotype of medical relevance.

Much research in recent years has focused on testing conditional independent hypotheses with False Discovery Rate (FDR) (Benjamini and Hochberg 1995) control, which provides findings that are informative, precise, and reproducible. An effective way of doing so is via the knockoff framework (Barber and Candès 2015, Candès *et al.* 2018, Sesia *et al.* 2019, He *et al.*, 2021a). Given *p* features $\left(X_{1},\ldots ,X_{p}\right)$ and a response *Y*, the conditional independence hypothesis $H_{j}$ is false only if variable $X_{j}$ carries information on *Y* that is not provided by the other variables under consideration. Formally,

(1) $$H_{j}:X_{j}⊥Y|X_{-j},$$

where $X_{-j}$ indicates all variables except for the *j*th one. The knockoff framework tests these hypotheses by comparing the signal in $X_{j}$ with that of artificial negative controls ${\tilde{X}}_{j}$.

The variables $\tilde{X}=({\tilde{X}}_{1},\ldots ,{\tilde{X}}_{p})$ are constructed without looking at *Y*, and the joint distribution of $\left(X,\tilde{X}\right)$ satisfies a pairwise exchangeability condition

$${\left(X,\tilde{X}\right)}_{\mathrm{swap}(A)}\overset{d}{=}(X,\tilde{X})$$

for any subset A⊆{1,…,p}. Above, ${\left(X,\tilde{X}\right)}_{\mathrm{swap}(A)}$ is obtained from $\left(X,\tilde{X}\right)$ by swapping the entries $X_{j}$ and ${\tilde{X}}_{j}$ for each j∈A. See Candès *et al.* (2018) for an overall description of the approach, as well as detailed discussion of motivation and assumptions.

Variables discovered by rejecting conditional hypotheses (1) are not “guilty by association,” but carry unique information among the features in the data. This precision, however, comes with a price: if a non-null variable is strongly correlated with a null one, the power to reject the associated conditional hypothesis can be diminished in finite samples. To remedy this, one can shift the object of inference from individual variables to groups of (highly correlated) variables. The knockoff framework can be adapted to this task, by constructing artificial control variables that conserve the joint distribution $\left(X_{j},X_{k}\right)$ only when *j* and *k* belong to different groups.

This idea of group knockoffs was introduced in Dai and Barber (2016) and developed by Katsevich and Sabatti (2019), who considered the model-X framework we focus on in this paper. Sesia *et al.* (2020, 2021) applied this extensively to the analysis of GWAS, showing how group knockoffs can be constructed when the distribution of $\left(X_{1},\ldots ,X_{p}\right)$ can be described with a hidden Markov model. For Gaussian designs, Spector and Janson (2022) carried out a proof-of-concept study utilizing more general group knockoff algorithms. However, their python package knockpy encounters a few computational challenges for application in large-scale datasets. All in all, there remains a paucity of efficient algorithms to generate group knockoffs.

We address this need by focusing on second-order knockoffs (Candès *et al.*, 2018). These are constructed to ensure that the necessary exchangeability properties hold up to the first two moments of the joint distribution of *X* and $\tilde{X}$, rather than for the entire distribution. This produces valid knockoffs when *X* has a Gaussian distribution and is a good approximation when the distribution of *X* can be effectively approximated as normal (Barber *et al.*, 2020). For example, the analysis of summary statistics coming from GWAS data is particularly well suited to second-order knockoffs. Furthermore, it is useful to point out that relying on second-order knockoffs has minimal impact on FDR control when the feature importance measures are based on second-order moments.

In this paper, we extend three optimization frameworks for the construction of knockoffs (Candès *et al.* 2018, Gimenez and Zou 2019, Spector and Janson 2022) to develop new group knockoffs algorithms. We then focus on the task of constructing group knockoffs for the analysis of GWAS summary statistics. Leveraging existing databases, we identify variance–covariance matrices representing the correlation between genetic variants. We conclude with various simulations and a detailed study of Albuminuria GWAS from the UK Biobank (Sudlow *et al.* 2015).

## 2 Problem statement

We are interested in discovering which among *p* features $X=(X_{1},\ldots ,X_{p})$ carries information on a response *Y*. We assume that the distribution of *X* is known, with variance–covariance matrix Σ, but make no assumptions on the distribution of Y|X. We consider a partition of [p]={1,2,…,p} into *g* disjoint groups of variables. For γ∈{1,2,…,g}, we denote by $A_{\gamma }$ and $X_{\gamma }$, respectively, the collection of indices corresponding to group γ and the vector of random variables in group γ, and by $X_{-\gamma }$ the collection of all other features. Without loss of generality, we assume that the indices of all variables in the same group are contiguous. Given *n* i.i.d. samples, we use $X\in R^{n\times p}$ to denote the stacked design matrix where each row is a sample. For other variables, we will use bolded uppercase text to denote matrices, unbolded uppercase for random variables, and bolded lowercase for vectors.

Throughout this paper, the problem of interest is to test the “group conditional independence hypotheses”

(2) $$H_{\gamma }:X_{\gamma }⊥Y|X_{-\gamma }$$

for 1≤γ≤g. The idea of exploring the joint influence of multiple correlated variables on an outcome has been proposed multiple times in the literature. When the data are not informative enough to distinguish between the contributions of single variable, moving the inference to the group level can yield an increase in power. For example, in the context of gene mapping, this approach is implemented in methods such as CAVIAR (Hormozdiari *et al.* 2014) and SuSiE (Wang *et al.* 2020). The knockoffs procedure can be adapted to test hypotheses of the form (2) by constructing artificial controls that are “indistinguishable” from the original variables at the group level. Specifically, as in Katsevich and Sabatti (2019), “group knockoffs” $\tilde{X}=({\tilde{X}}_{1},\ldots ,{\tilde{X}}_{p})$ for the variables $X=(X_{1},\ldots ,X_{p})$ are defined by the following properties:

(3) $$\tilde{X}⊥Y|X$$

(4) $${\left(X,\tilde{X}\right)}_{\text{swap}(C)}\overset{d}{=}(X,\tilde{X})\;\;\;\;\forall \;\;C:\;\;C=\cup_{\gamma \in S}A_{\gamma }$$

where S is a set of groups γ, and ${\left(X,\tilde{X}\right)}_{\text{swap}(C)}$ is formed by swapping $X_{j}$ with ${\tilde{X}}_{j}$ for every index *j* in C. Condition (4) is what distinguishes group knockoffs from individual variable knockoffs. The latter require the distributional invariance to hold for all sets C, not just those that can be described as a union of groups. Since fewer exchangeability restrictions are imposed, group knockoffs have added flexibility that can be leveraged for increased power (see online supplementary material S8.1 for illustration).

As in Candès *et al.* (2018), rather than requiring that ${\left(X,\tilde{X}\right)}_{\text{swap}(C)}$ and $\left(X,\tilde{X}\right)$ have the same distribution for any C, we can ask that they have the same first two moments, i.e. the same mean and covariance. This approximate construction is known as “second-order” knockoffs and is the focus of the present paper. If all variables have been standardized to have mean zero, the second-order version of condition (4) translates to the following property of the correlation matrix of $\left(X,\tilde{X}\right)$:

(5) $$\begin{array}{c} \text{Cov}(X,\tilde{X})=G_{S}=\left[\begin{array}{cc} \Sigma & \Sigma -S\\ \Sigma -S & \Sigma \end{array}\right]\in R^{2p\times 2p}, \end{array}$$

with S block-diagonal, where the blocks correspond to the groups γ∈{1,…,g}. That is, $S_{ij}=0$ whenever indices *i* and *j* are not in the same group of the partition. For ease of notation, we thus write $S=\text{diag}\{S_{1},\ldots ,S_{g}\}$, where $S_{i}$ is the $p_{i}\times p_{i}$ symmetric matrix for the *i*th group. Again, it is useful to contrast (5) with the analogous requirement for second-order (single variables) knockoffs, where S needs to be a diagonal matrix. When trying to make inferences at the group level, only correlations across groups need to be conserved, resulting in greater degrees of freedom in the construction of knockoffs. In addition, note that S has to be chosen such that $G_{S}$ remains a valid 2p×2p covariance matrix, leading to the constraints that S≽0^1^ and 2Σ−S≽0, as in Barber and Candès (2015) and Candès *et al.* (2018). In particular, every $S_{i}$ satisfies $S_{i}≽0$.

What choice of S leads to strong performance? While any valid knockoff will guarantee FDR control, we aim for a construction that leads to high power. To develop intuition about this question, it is useful to recall how knockoffs are used for inference on the hypotheses (2).

Once valid group knockoffs $\tilde{X}$ have been constructed for each observation, the augmented data $\left(Y,X,\tilde{X}\right)$ is used to obtain a vector of feature importance statistics $(Z,\tilde{Z})\in R^{2g}$ where, for γ≤g, $Z_{\gamma }$ and ${\tilde{Z}}_{\gamma }$ reflect the importance of group γ and its corresponding set of knockoffs. The only requirement on $\left(Z,\tilde{Z}\right)$ is that swapping all variables in group γ with their corresponding knockoffs also swaps the values of $Z_{\gamma }$ and ${\tilde{Z}}_{\gamma }$. Next, define the knockoff score $W_{\gamma }=w(Z_{\gamma },{\tilde{Z}}_{\gamma })$, where *w* is any antisymmetric function (e.g. w(x,y)=x−y). Intuitively, $W_{\gamma }$ measures the relative importance of group γ as compared to its group of knockoffs. $W_{\gamma }$ should therefore be large and positive for groups with true importance and is equally likely to be positive or negative for null groups. The procedure concludes by rejecting all groups γ for which $W_{\gamma }\geq \tau$, where the data-dependent threshold τ is computed from the knockoff filter (Barber and Candès 2015).

Given that the key to the selection of group γ is the difference between the feature importance statistics $Z_{\gamma }$ and $Z_{\gamma +g}$, it is natural to aim for a construction that makes *X* and $\tilde{X}$ as distinguishable as possible. Here, we describe from the literature, three popular criteria for this task and generalize them to the group setting.

The first suggestion implemented in the literature is to “minimize the average correlation” between $X_{j}$ and ${\tilde{X}}_{j}$ (Barber and Candès 2015, Candès *et al.* 2018), which Dai and Barber (2016) generalize in principle to the group setting. We refer to this as the SDP criterion, since the underlying optimization problem requires solving a semi-definite programming problem. This can be formulated as

(SDP) $$\underset{0\underline{≺}S\underline{≺}2\Sigma }{\min}\sum_{\gamma =1}^{g}\frac{1}{|A_{\gamma }{|}^{2}}\sum_{i,j\in A_{\gamma }}|S_{ij}-\Sigma_{ij}|.$$

Here, the $|A_{\gamma }{|}^{2}$ terms normalize each group by its size to prevent larger groups from dominating the criterion, since all groups ought to matter equally for power consideration. In fact, Dai and Barber (2016) consider only a special case of this problem, where $S_{\gamma }=\tau \cdot \Sigma_{\gamma },$ with $\Sigma_{\gamma }$ the submatrix of Σ corresponding to the variables in group γ. This simplification (which we refer to as equivariant SDP or eSDP) amounts to choosing $\tau =\min\left\{1,2\cdot \lambda_{\min}(B\Sigma B)\right\}$ where $B=\mathrm{diag}\left\{\Sigma_{1}^{-1/2},\ldots ,\Sigma_{g}^{-1/2}\right\}$. Algorithms to solve the more general problem have not been described previously.

Working with individual-level knockoffs, Gimenez and Zou (2019) explored a different suggestion: minimizing the mutual information, or “maximizing the entropy” (ME), between *X* and $\tilde{X}$. In the case of Gaussian variables, the entropy of $\left(X,\tilde{X}\right)$ is proportional to the log-determinant of its covariance matrix. In the single-variable setting, the objective is

(ME) $$\underset{0\underline{≺}S\underline{≺}2\Sigma }{\min}\text{log det}(G_{S}^{-1})$$

with a diagonal matrix S. In the group setting, the diagonal constraint on S translates to a group-block-diagonal constraint as before. In both cases, the entropy maximization program is convex in S.

Finally, Spector and Janson (2022) introduce another criterion that, like ME, focuses on minimizing the reconstructability of $X_{j}$ from $\tilde{X}$. Knockoffs that satisfy the “minimum variance-based reconstructability” (MVR) criterion minimize $\sum_{j=1}^{p}(E[\text{Var}(X_{j}|X_{-j},\tilde{X})]{)}^{-1}.$ In the case of Gaussian variables, or under the second-order approximation, this is equivalent to

(MVR) $$\underset{0\underline{≺}S\underline{≺}2\Sigma }{\min}\text{Tr}(G_{S}^{-1}).$$

Spector and Janson (2022), working primarily with individual level knockoffs, show how ME and MVR can yield better power than SDP.

To summarize, we consider three second-order group knockoff construction procedures, each minimizing a different loss function:

$$\begin{array}{c} L_{\text{SDP}}(S)=\sum_{\gamma =1}^{g}\frac{1}{|A_{\gamma }{|}^{2}}\sum_{i,j\in A_{\gamma }}|S_{ij}-\Sigma_{ij}|\\ L_{\text{MVR}}(S)=\text{Tr}(G_{S}^{-1})\\ L_{\text{ME}}(S)=\text{logdet}(G_{S}^{-1}) \end{array}$$

under the constraints that $0\underline{≺}S\underline{≺}2\Sigma$ and $S=\text{diag}(S_{1},\ldots ,S_{g})$.

In the interest of generality, we will work with the multiple knockoff filter of Gimenez and Zou (2019). That is, instead of generating one knockoff copy of each observation, we will generate *m* and suitably aggregate them for inferential purposes. This approach was used extensively in He *et al.* (2021a, 2022) to improve the power and stability of model-X knockoffs. For second-order knockoffs, this simply means that we will need to use a variance–covariance matrix of the form

(6) $$\begin{array}{cc} G_{S} & =\left[\begin{array}{cccc} \Sigma & \Sigma -S & \cdots & \Sigma -S\\ \Sigma -S & \Sigma & \cdots & \Sigma -S\\ \vdots & \vdots & \ddots & \vdots \\ \Sigma -S & \cdots & \cdots & \Sigma \end{array}\right]\in R^{p(m+1)\times p(m+1)} \end{array}$$

with the new constraints $0\underline{≺}S\underline{≺}\frac{m+1}{m}\Sigma$ and $S=\text{diag}(S_{1},\ldots ,S_{g})$ (note that this recovers the original constraints when m=1).

## 3 Algorithms for second-order group knockoffs

While the modification of the objective from individual level to group knockoffs is formally straightforward, the fact that S becomes block-diagonal poses computational challenges. The added degrees of freedom in the knockoff construction can translate to higher power, but also to a much larger number of variables to optimize over. Indeed, Dai and Barber (2016) immediately reduce the dimensions of the problem by imposing the structure $S_{\gamma }=\tau \cdot \Sigma_{\gamma }$ for all γ. While this leads to a convenient one-dimensional problem, it counters the increased flexibility of group knockoffs, reducing power in a number of situations.

Our goal is to design computationally feasible algorithms that allow researchers to use any of the second-order group knockoff constructions while optimizing over all of S. To do so, we combine full coordinate descent with some coarser updates—somewhat inspired by Dai and Barber (2016); Askari *et al.* (2021); and Spector and Janson (2022).

We start by describing a general coordinate descent strategy, which optimizes over every non-zero entry of S by sequentially updating as

(7) $$S_{ij}^{\mathrm{new}}=S_{ij}+\delta_{ij}.$$

We impose symmetry on S, so we must also have $S_{ji}^{\mathrm{new}}=S_{ij}^{\mathrm{new}}$. Spector and Janson (2022) briefly explored a similar form of update in their software, although a detailed comparison is difficult since their algorithm is sparsely documented. Our approach here is derived independently.

In our approach, there are three key steps in performing this update. The first is identifying the valid values of $\delta_{ij}$. If we write $D=\frac{m+1}{m}\Sigma -S$, then the positive definiteness constraints require that both $S^{\mathrm{new}}$ and $D^{\mathrm{new}}=\frac{m+1}{m}\Sigma -S^{\mathrm{new}}$ are positive definite. If $e_{i},e_{j}$ are the *i*th and *j*th basis vector, then online supplementary material S1.6–S1.7 shows that this feasible region is

(8) $$\frac{-1}{e_{j}^{t}S^{-1}e_{j}}\leq \delta_{ij}\leq \frac{1}{e_{j}^{t}D^{-1}e_{j}}$$

if i=j, otherwise it is

$$\begin{array}{c} \frac{-2}{e_{i}^{t}S^{-1}e_{i}+2e_{i}^{t}S^{-1}e_{j}+e_{j}^{t}S^{-1}e_{j}}\leq \delta_{ij}\leq \\ \frac{2}{e_{i}^{t}D^{-1}e_{i}+2e_{i}^{t}D^{-1}e_{j}+e_{j}^{t}D^{-1}e_{j}}. \end{array}$$

Both region contains $\delta_{ij}=0$. To prevent $\delta_{ij}$ from reaching the boundary condition, which yields S or D numerically singular, in practice, we take away $\epsilon =10^{-6}$ from both ends.

The second step is to optimize for $\delta_{ij}$ in this region for different objective functions, as detailed in the online supplementary material S1.1–S1.5. In the case of the ME objective, we can solve for $\delta_{ij}$ by maximizing

(9) $$\begin{array}{c} g(\delta_{ij})=\text{log}\;\left({\left(1-\delta_{ij}e_{i}^{t}D^{-1}e_{j}\right)}^{2}-\delta_{ij}^{2}e_{i}^{t}D^{-1}e_{i}e_{j}^{t}D^{-1}e_{j}\right)\\ +m\;\text{log}\;\left({\left(1+\delta_{ij}e_{i}^{t}S^{-1}e_{j}\right)}^{2}-\delta_{ij}^{2}e_{j}^{t}S^{-1}e_{j}e_{i}^{t}S^{-1}e_{i}\right). \end{array}$$

If all constants such as $e_{i}^{t}S^{-1}e_{i}$ and $e_{i}^{t}D^{-1}e_{i}$ are stored, then $g(\delta_{ij})$ is a scalar-valued function with scalar inputs, and is therefore easy to optimize within an interval.

The final step is to update the stored terms such as $e_{i}^{t}S^{-1}e_{i}$ and $e_{i}^{t}D^{-1}e_{i}$. We adopt the approach in Askari *et al.* (2021) and Spector and Janson (2022) by precomputing and maintaining Cholesky factors of $D=LL^{t}$ and $S=CC^{t}$. Constants such as $e_{i}^{t}D^{-1}e_{j}$ can then be efficiently computed by noting that $e_{i}^{t}D^{-1}e_{j}=e_{i}^{t}{\left(LL^{t}\right)}^{-1}e_{j}={\left(L^{-1}e_{i}\right)}^{t}(L^{-1}e_{j})\equiv v^{t}u$, where v and u can be computed via forward–backward substitution on $\mathrm{Lu}=e_{j}$ and $\mathrm{Lv}=e_{i}$. After updating $S_{ij}$ and $S_{ji}$, we perform a rank-2 update to the Cholesky factors to maintain the equalities $L_{\mathrm{new}}L_{\mathrm{new}}^{t}=\frac{m+1}{m}\Sigma -S_{\mathrm{new}}$ and $C_{\mathrm{new}}C_{\mathrm{new}}^{t}=S_{\mathrm{new}}$. Full details are available in online supplementary material S1.8.

The coordinate descent updates (7) allow optimization over all free parameters of S rather than reducing the dimension of the problem. However, the local nature of these moves encounters two connected challenges. First, given a large number of optimization variables, a complete update of all elements of S can require substantial time (e.g. if a group comprises 1000 elements, then that single group would contribute $10^{6}$ optimization variables). Second, even if the objectives are convex, coordinate descent can become stuck at a local minimum due to the presence of constraints (positive definiteness of S and D).

To alleviate these difficulties, we found it useful to augment our iterative procedure with “global” updates, which restrict the set of possible values for S, in a similar but less limiting way than what is described in Dai and Barber (2016). Specifically, we consider the following “PCA updates”:

(10) $$S^{\mathrm{new}}=S+\delta_{i}v_{i}v_{i}^{t},$$

where i∈[p] and each $v_{i}$ are precomputed vectors such that the outer product $v_{i}v_{i}^{t}$ respects the block diagonal structure of S. In practice, we choose $v_{1},\ldots ,v_{p}$ to be the eigenvectors of the block-diagonal matrix

(11) $$\begin{array}{cc} \Sigma_{\mathrm{block}} & =\left[\begin{array}{ccc} \Sigma_{1} & & \\ & \ddots & \\ & & \Sigma_{g} \end{array}\right] \end{array}$$

In the case of the ME criterion, we can explicitly compute

(12) $$\delta_{i}=\frac{mv_{i}^{t}S^{-1}v_{i}-v_{i}^{t}D^{-1}v_{i}}{(m+1)v_{i}^{t}S^{-1}v_{i}v_{i}^{t}D^{-1}v_{i}},$$

which conveniently lies in the feasible region

(13) $$\frac{-1}{v_{i}^{t}S^{-1}v_{i}}\leq \delta_{i}\leq \frac{1}{v_{i}^{t}D^{-1}v_{i}}.$$

Each full iteration now requires the optimization of only *p* variables, so the computational complexity is equivalent to that of standard coordinate descent model-X knockoffs, scaling as $O(p^{3})$ (Askari *et al.* 2021). Full details of such PCA updates are provided in online supplementary material S2.

In practice, we find that alternating between updates of types (7) and (10) greatly reduces the possibility of the algorithm being trapped in a local minimum while maintaining a small overall computational cost. This immediately yields the question of how often one should alternate between general coordinate descent (7) and the faster but more restrictive PCA updates (10). Our numerical experiments with a few alternating schemes produce no clear winner. Thus, by default, our software performs them back-to-back, and we leave it as an option for the user to adjust. The overall algorithm is summarized in Algorithm 1.

Algorithm 1: Scheme of iterative solution of (SDP),(ME), (MVR)

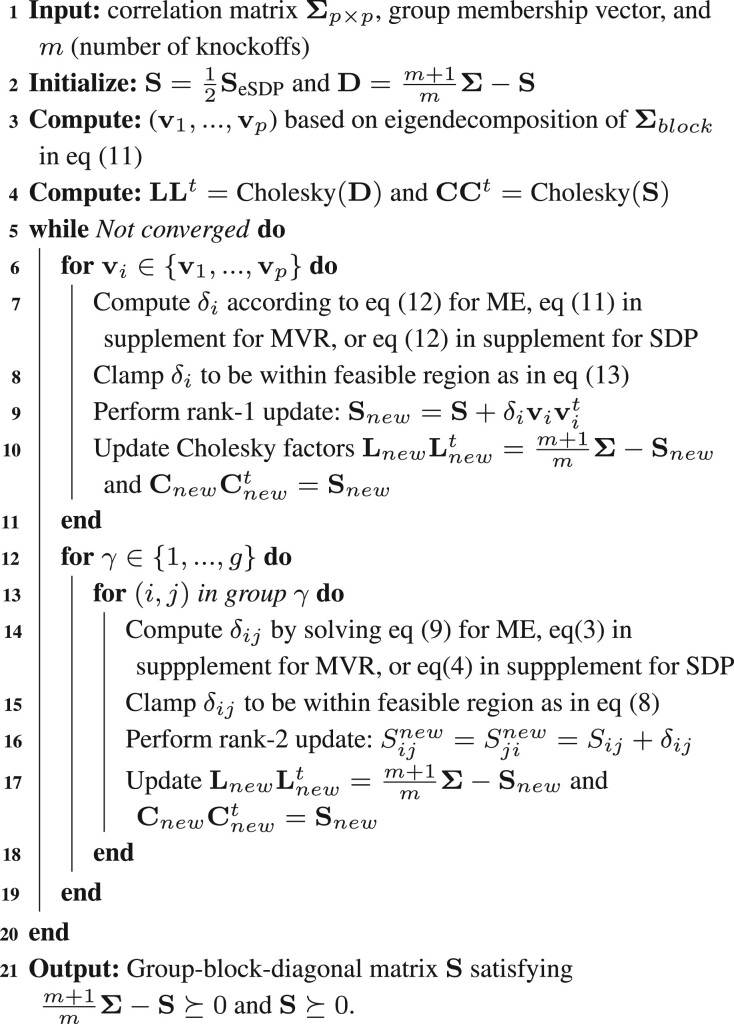

## 4 Exploiting conditional independence

While the approaches just described allow us to identify S that minimizes any of the three loss function, depending on the size of the problem (defined both by *p* and the size of the largest groups), the solution can be computationally intensive. To avoid unnecessary costs, it is important to leverage structure in the distribution of *X*, when this is possible. Bates *et al.* (2021) discuss the general computational advantages in knockoff construction associated with conditional independence and Sesia *et al.* (2019) leverages these in the context of hidden Markov models (HMM) (Theorem 1), while Sesia *et al.* (2020) extend the same construction to group knockoffs (see Proposition 2 in the online supplementary material).

We consider here a particular form of conditional independence, linked to the group structure.

Definition 1 *Let X be p random variables, partitioned in groups*γ=1,…g*. We say that their distribution*$P_{X}$*has a group-key conditional independence property with respect to the partition*${\left\{A_{\gamma }\right\}}_{\gamma =1}^{g}$*if for each*γ*there exists two disjoint subsets*$A_{\gamma }^{†}$*and*$A_{\gamma }^{⋆}$*such that*

$$\begin{array}{c} A_{\gamma }=A_{\gamma }^{†}\cup A_{\gamma }^{⋆}\\ X_{\gamma }^{†}⊥⊥X_{-\gamma }|X_{\gamma }^{⋆},\;\;\gamma \in [g], \end{array}$$

*where*$X_{\gamma }^{†}$*(*$X_{\gamma }^{⋆}$*) collects the variables*$X_{i}$*with*$i\in A_{\gamma }^{†}$*(*$A_{\gamma }^{⋆}$*).*

Algorithm 2. Group knockoffs under group key conditional independence (Definition 1).

**1 Input:** Realizations of the variables *X*, and their joint distribution.

**2** Generate valid group knockoffs ${\tilde{X}}^{⋆}$ of $X^{⋆}=(X_{1}^{⋆},\ldots ,X_{g}^{⋆})$.

**3** Compute the conditional distribution $F_{\gamma }$ of $X_{\gamma }^{†}$ given $X^{⋆}$ (γ=1,…,g).

**4** Generate knockoffs ${\tilde{X}}_{\gamma }^{†}∼F_{\gamma }(\cdot |{\tilde{X}}^{⋆})$ (γ=1,…,g).

**5 Output:** Group knockoffs $\left({\tilde{X}}_{1}^{⋆},\tilde{X}{}_{1}^{†}),\ldots ,(\tilde{X}{}_{g}^{⋆},\tilde{X}{}_{g}^{†}\right)$.

Definition 1 describes a setting where, within each group γ, there is a subset of key variables that “drive” the dependence across groups, and conditioning on these all other variables are independent across groups. Algorithm 2 capitalizes on this conditional independence for the construction of knockoffs. While it resembles that adopted for HMM in Sesia *et al.* (2019), the conditional independence structure it rests upon is different: for each γ∈[g], the size of both $A_{\gamma }^{†}$ and $A_{\gamma }^{⋆}$ is arbitrary and within each group the variables $X_{\gamma }^{†}$ are not independent given $X_{\gamma }^{⋆}$. In the interest of simplicity, we have outlined Algorithm 2 for one knockoff copy, but it easily extends to creating *m* knockoff copies, by generating ${\tilde{X}}^{⋆(1)},\ldots {\tilde{X}}^{⋆(m)}$ in Step 2, and *m*-independent copies in step 4. Finally, note that Algorithm 2 is stated for exact knockoff construction (requiring knowledge on the joint distribution of *X*), even if in the context of this paper, we will use it for second-order knockoffs.

Theorem 1 *Knockoffs*$\tilde{X}$*generated under Algorithm 2, are valid knockoffs for groups*${\left\{A_{\gamma }\right\}}_{\gamma =1}^{g}$*when the distribution of X has group key conditional independence with respect to*${\left\{A_{\gamma }\right\}}_{\gamma =1}^{g}$.

Theorem 1—proven in the online supplementary material S3—assures the validity of Algorithm 2. Assuming that sampling from $F_{\gamma }$ in step 3 is easy, the computational advantages of Algorithm 2 lie in the reduction of the number of the optimization variables. As group knockoffs have to be constructed only for $\left\{A_{\gamma }^{⋆}:\gamma \in [g]\right\}$, we only have to minimize either

$$\begin{array}{c} L_{\text{SDP}}^{⋆}(S^{⋆})=\sum_{\gamma =1}^{g}\frac{1}{|A_{\gamma }^{⋆}{|}^{2}}\sum_{i,j\in A_{\gamma }^{⋆}}|S_{ij}-\Sigma_{ij}|,\\ L_{\text{MVR}}^{⋆}(S^{⋆})=\text{Tr}({\left(G_{S^{⋆}}^{⋆}\right)}^{-1}),\;\;\text{or}\\ L_{\text{ME}}^{⋆}(S^{⋆})=\text{logdet}({\left(G_{S^{⋆}}^{⋆}\right)}^{-1}). \end{array}$$

This reduces the number of optimization variables from $\sum_{\gamma =1}^{g}|A_{\gamma }{|}^{2}$ to $\sum_{\gamma =1}^{g}|A_{\gamma }^{⋆}{|}^{2}$, which can be significant. Supplementary material S8.4 compares the runtime of standard group knockoffs to those utilizing Algorithm 2, empirically achieving 10-100x speedup. 

Algorithm 2, leads to valid knockoffs with potential computational savings. But how “good” are these knockoffs? How do they compare to those constructed to satisfy any of the optimality criteria we considered in the previous section? Theorem 2 (proof in the online supplementary material S4) offers some light on this topic: when ME is the criteria of choice, the group knockoffs constructed using the variance–covariance matrix $G_{S}$ from Algorithm 1 have the same distribution as those constructed starting from $G_{S^{⋆}}^{⋆}$ and following Algorithm 2.

Theorem 2 *Let*${\left\{A_{\gamma }\right\}}_{\gamma =1}^{g}$*be a collection of groups for the variables in X; and let X have the group key conditional independence property with respect to*${\left\{A_{\gamma }\right\}}_{\gamma =1}^{g}$*. Let*$\tilde{X}$*be knockoffs generated according to Algorithm 2, and such that the distribution of*$\left(X^{⋆},{\tilde{X}}^{⋆}\right)$*minimizes*$L_{\text{ME}}^{⋆}(S^{⋆})$*. Then, the distribution of*$\left(X,\tilde{X}\right)$*minimizes*$L_{\text{ME}}(S)$.

Theorems 1 and 2 taken together suggest that, if one is interested in ME group-knockoffs, a particularly convenient computational approach is available when the distribution of *X* follows a precise form of conditional independence.

Unfortunately, however, it is unrealistic to assume that the property described by Definition 1 holds exactly, and that we have knowledge of the variables in the ⋆ set. This being said, especially in the context of second-order (approximate) knockoffs, it is meaningful to try to identify a partition of the *p* variables and a subset of key variables in each of the groups, so as to approximate the true distribution of *X* with one that has the desired properties. The online supplementary material S5 describes a heuristic approach to solve this problem. While we can make no general statement about its validity, we have studied its performance for genetic data, as we will detail in the following section.

## 5 Group knockoffs for GWAS summary statistics

Before applying our group knockoff algorithms, let us first discuss an application of second-order group knockoffs, which is a major motivation for this paper. Sharing individual-level genotype data is generally a difficult process due to privacy and security issues and distribution of summary statistics continues to serve as an attractive option for geneticists. Is it possible to carry out an analysis with the attractive properties of knockoffs starting from this summary data?

He *et al.* (2022) described how conditional independence hypotheses can be tested using a knockoff framework even without access to X and y. This procedure, called *ghost-knockoff*, relies purely on summary statistics and achieves a power only slightly reduced to that of an analysis based on X and y directly. In a nutshell, if the feature importance statistic used in the test of $H_{j}$ (1) is the marginal Z-scores $Z_{j}=\frac{1}{\sqrt{n}}x_{j}^{t}y\in R$ ($x_{j}$ is the *j*th column of X), we can sample a value for the corresponding ${\tilde{Z}}_{j}$ directly without having to generate knockoffs for all the distinct observations. This relies also on the fact that under the model-X knockoff framework, y is fixed and $x_{j}$ is a randomly sampled genotype vector, so $x_{j}^{t}y$ is the sum of random variables, which, by the central limit theorem, converges to a Gaussian distribution.

In a companion work (Chen *et al.* 2024b), we show how a similar approach can be extended to feature importance statistics derived from a lasso-like procedure, which takes as input marginal Z-scores and covariance matrix Σ, where it is assumed that $\left(Z_{1},\ldots ,Z_{p})∼N(0,\Sigma \right)$ and Σ can be estimated. In both cases, once we move from individual level data to summary statistics, we work with Gaussian distributions, making the use of second-order knockoffs particularly appropriate.

When using importance measures derived from penalized regression, we increase our ability to resolve signals. At the same time, the presence of tightly linked variants can lead to highly correlated Z-scores, which can diminish power. Moving to testing of group hypotheses with group knockoffs can alleviate this difficulty.

Given this premise, it is then useful to clarify in the GWAS context how information on the relevant variance–covariance matrices can be gathered, how groups can be identified, and how well the genotype distribution can be approximated with one satisfying group-key conditional independence.

Supplementary Fig. S3 summarizes the interacting pieces for group knockoffs application in GWAS, which are roughly broken down into the following three main steps.

### 5.1 Obtaining suitable variance–covariance matrices

Large consortiums such as Pan-UKB (Pan-UKB team 2020) and gnomAD (Chen *et al.* 2024a) make available sample variance–covariance matrices calculated on genotypes of individuals from different human populations. We rely on the European Pan-UKB panel containing p≈24 million variants across the human genome derived from $\approx 5\times 10^{5}$ British samples. We restricted our attention to variants present in the UK Biobank GWAS genotype array (Sudlow *et al.* 2015) with minor allele frequency exceeding 0.01 in the Pan-UKB panel.

Given ${\hat{\Sigma }}_{\text{PanUK}}$, we identified approximately independent blocks of SNPs by directly adapting the output of ldetect (Berisa and Pickrell 2016). A total of 1703 blocks ${\hat{\Sigma }}_{1},\ldots ,{\hat{\Sigma }}_{1703}$ of size varying between $∼10^{2}$ and $∼10^{3}$ were identified, see online supplementary material S7.1 for summary statistics. To ensure we obtained a reliable estimate of the true population SNP variance–covariance matrix, we regularized the resulting $\Sigma_{i}$s in multiple ways, as detailed in online supplementary material S7.2. These matrices are then used for knockoff generation and computing feature importance statistics, as described below.

### 5.2 Defining groups and key-variables

Within each of the 1703 blocks, we partition the SNPs into groups using average linkage hierarchical clustering with correlation cutoff 0.5 (see online supplementary material S5.1). To identify a set of key variables in each group such that the conditional independence described in Definition 1 might hold, we use Algorithm A2 in the online supplementary material (unless otherwise specified, we use c=0.5), which is motivated by the recent best subset selection algorithms (Sood and Hastie 2023). Supplementary Fig. S1 report information on the size of the groups and group key-variables identified.

### 5.3 Sampling knockoffs and inference

Given the block structure of $\hat{\Sigma }=\mathrm{diag}({\hat{\Sigma }}_{1},\ldots ,{\hat{\Sigma }}_{1703})$, we solve 1703 separate problems by applying Algorithm 2 to each ${\hat{\Sigma }}_{i}$ in parallel to identify the $S_{i}$ that minimizes each of the three criteria (SDP, ME, MVR). Once these are obtained, we sampled m=5 knockoffs ${\tilde{z}}_{i}=({\tilde{z}}_{i1},\ldots ,{\tilde{z}}_{im})\in R^{mp_{i}}$ by ${\tilde{z}}_{i}=P_{i}z_{i}+N(0,V_{i})$ where $P_{i}$ and $V_{i}$ are defined in equations (12) and (13) of He *et al.* (2022), which depend solely on $S_{i}$ and $\Sigma_{i}$. The final z-score knockoffs are formed by $\tilde{z}=({\tilde{z}}_{1},\ldots ,{\tilde{z}}_{1703})\in R^{mp}$.

Finally, we solve the pseudo-Lasso problem (Chen *et al.* 2024b) jointly over all blocks to define feature importance scores. This provides enhanced power compared to directly using the difference between z and $\tilde{z}$ (see online supplementary material S8.5). The unknown hyperparameter λ is tuned via the pseudo-validation approach (Mak *et al.* 2017, Zhang *et al.* 2021), see online supplementary material 7.4 for details. In practice, we use the BASIL algorithm (Qian *et al.* 2020) in the R package ghostbasil (Yang and Hastie 2023) to carry out the Lasso regression step. Although we split knockoff construction over multiple blocks, the Lasso regression step includes ≈3.6 million variables, including 0.6 million Z scores and their knockoffs.

## 6 Results

### 6.1 Simulation studies

We conduct two sets of simulations. In one, we use artificially generated covariance matrices Σ, so as to explore the performance of knockoff constructions under different and controlled forms of dependence. In the other, we use correlation matrices for SNP data derived from the Pan-UKB panel to investigate how well the proposed methods lend themselves to the analysis of genetic data.

The knockoff score is defined as the absolute value of the group-wise Lasso coefficient difference: $Z_{\gamma }=\sum_{i\in A_{\gamma }}|\beta_{i}|$ and ${\tilde{Z}}_{\gamma }^{\left(\ell \right)}=\sum_{i\in A_{\gamma }}|{\tilde{\beta }}_{i}^{\left(\ell \right)}|$ for ℓ=1,…,m, where $\beta =(\beta ,{\tilde{\beta }}^{1},\ldots ,{\tilde{\beta }}^{m})$ is the estimated effect sizes from performing Lasso regression on $\left(X,{\tilde{X}}^{1},\ldots ,{\tilde{X}}^{m}\right)$. The feature importance score for group γ is

$$W_{\gamma }=(Z_{\gamma }-\mathrm{median}({\tilde{Z}}_{\gamma }^{\left(1\right)},\ldots ,{\tilde{Z}}_{\gamma }^{\left(m\right)}))I_{Z_{\gamma }\geq \max({\tilde{Z}}_{\gamma }^{\left(1\right)},\ldots ,{\tilde{Z}}_{\gamma }^{\left(m\right)})}$$

*I* is the indicator function and $W_{\gamma }$ is the feature-importance statistic first introduced in He *et al.* (2021b). Groups with $W_{\gamma }>\tau$ are selected, where τ is calculated from the multiple knockoff filter (Gimenez and Zou 2019).

#### 6.1.1 Power/FDR of different knockoffs under special covariances

We consider five types of covariance $\Sigma_{p\times p}$ as described in online supplementary material S8.2, with p=1000 and always scaling Σ back to a matrix with ones on the diagonal. Here we generate m=5 knockoffs for each experiment. For each of the five special covariance matrices, we simulate 100 copies of Σ, and the corresponding data matrix X is formed by drawing *n* independent samples from N(0,Σ), where *n* is varied as a parameter. The response is simulated as $y=X\beta +N(0,I_{p\times p})$. The true regression vector $\beta \in R^{p}$ has k=50 non-zero coefficients randomly chosen across the *p* features with effect size $\beta_{j}∼N(0,1)$. Groups are defined on the basis of the observed data X using average linkage hierarchical clustering with correlation cutoff 0.5 (see online supplementary material S5.1). To compute group power and group FDR, a group is considered a true null when it contains no causal features, and a group is correctly rejected if it contains at least one causal feature. Power is reported as the fraction of groups correctly discovered among all causal groups, while FDR is the fraction of falsely discovered groups among all discoveries.

**Figure 1.**
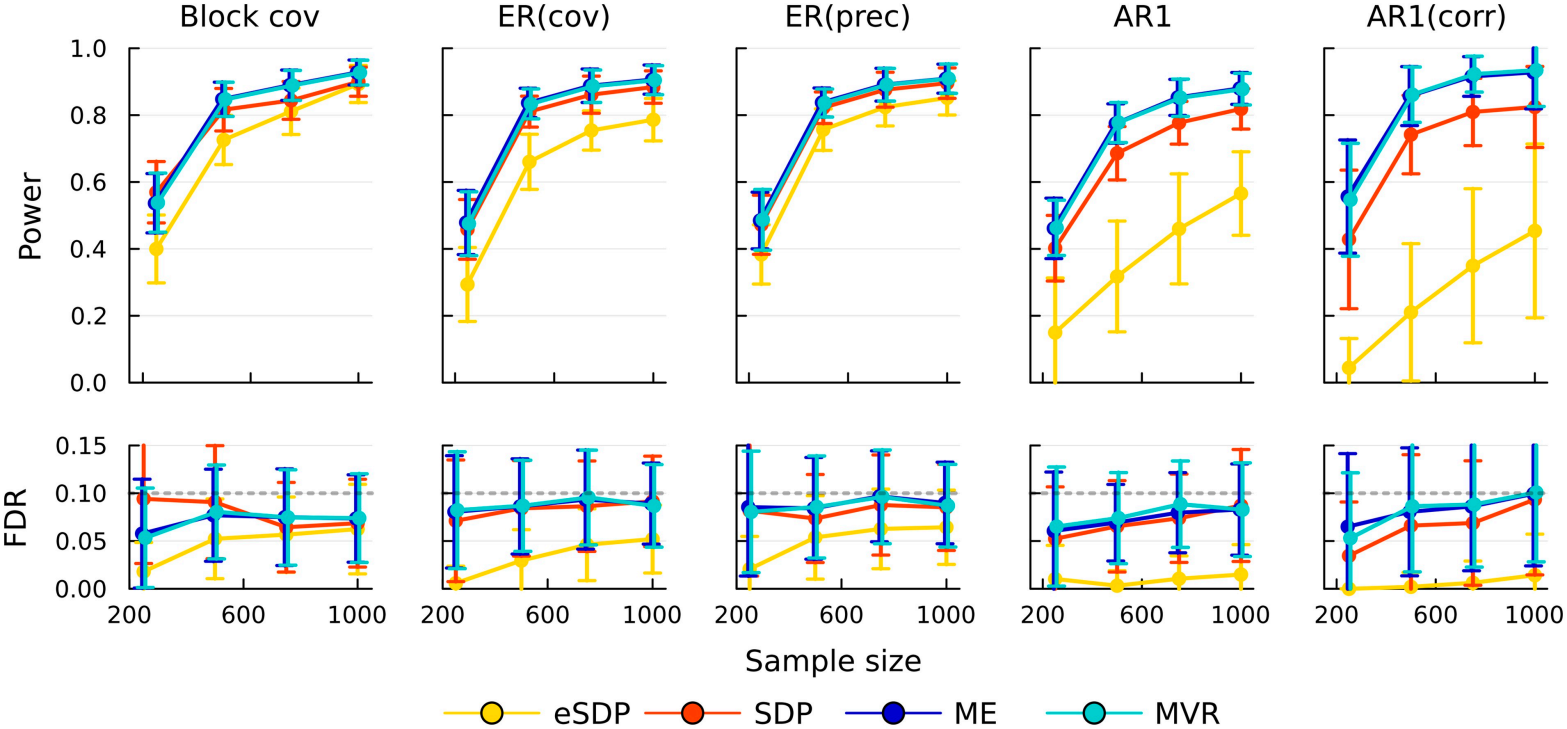
Power/FDR comparison of eSDP, SDP, MVR, and ME group knockoffs across different variance-covariance structure. Each point is average of 100 replicates. Timing results are available in Table 1.

Figure 1 compares the performance of different knockoff constructions with reference to power and FDR, averaging over 100 simulations. All methods control the FDR at target (10%) level. Across different covariance matrices, ME and MVR solvers generally have the best power, followed by SDP, followed by eSDP knockoffs. This behavior is consistent with regular (non-grouped) knockoffs (Spector and Janson 2022). For an indication of the computation time associated with the different methods, see Table 1. A more comprehensive analysis of timing is presented later.

**Table 1.** Runtime (in seconds) of group knockoff optimization for Fig. 1, averaged over 100 simulations.^a^

Model	eSDP	SDP	ME	MVR
Block Cov	4.1±0.5	244.2±141.2	18.8±4.1	33.6±5.9
ER (cov)	2.8±0.4	3858.2±1035.7	157.8±47.1	124.3±38.9
ER (prec)	3.8±0.5	6738.1±1755.9	69.0±17.9	92.0±26.4
AR1	4.0±1.2	668.8±418.3	56.9±32.9	92.6±51.4
AR1 (corr)	4.3±1.3	973.3±580.0	172.4±106.0	225.4±132.9

a There are p=1000 variables, and groups are constructed empirically based on the data matrix X. More comprehensive timing comparisons are presented in Supplementary Fig. S6.

#### 6.1.2 Power/FDR of group-key conditional indep. and genetic data

Here, we use real UKB genotypes (Sudlow *et al.* 2015) in conjunction with covariance matrices extracted from Pan-UKB (Pan-UKB team 2020) to conduct simulations. The goal here is (a) to ensure that our approximately independent blocks of SNPs identified in section 5.1 are really independent, (b) verify that the distribution of genotypes can be approximated by a Gaussian, and (c) explore to what extent the group-key conditional independent hypothesis is appropriate for these matrices.

First, we randomly select n=2000 British samples from the UK Biobank and restricted our attention to p=8844 SNPs residing on chromosome 22. All SNPs are centered to mean 0 variance 1, and partitioned into 24 roughly independent blocks as described in section 5.1. Given this subset of individual-level genotypes, we simulate the response $y=X\beta +N(0,I_{n\times n})$ where β contain k=20 non-zero effects chosen randomly across the chromosome and effect sizes drawn from N(0,0.2). The resulting phenotypes have heritability $h^{2}\approx 0.438$. After forming the phenotypes, we generate m=1 second-order knockoffs where the covariance matrix Σ is estimated as $\hat{\Sigma }=\mathrm{diag}({\hat{\Sigma }}_{\mathrm{chr}22,1},\ldots ,{\hat{\Sigma }}_{\mathrm{chr}22,24})$ and each ${\hat{\Sigma }}_{\mathrm{chr}22,i}$ is extracted from the corresponding region in the Pan-UKB. Importantly, the original genotypes X have entries {0,1,2} (prior to centering/scaling) but their knockoffs are generated from a Gaussian distribution with the covariance estimated without using the original X. We define groups with average linkage hierarchical clustering on each $\Sigma_{\mathrm{chr}22,i}$ with correlation cutoff 0.5, then identify key variables with Algorithm A2 in the online supplementary material for c∈{0.25,0.5,0.75,1.0}. Note that c=1 is equivalent to not using the conditional independence assumption, and when c<1.0, it is possible for a causal variant to not be selected as a key variable. We ran 100 independent simulations with this setup and averaged the power/FDR.

**Figure 2.**
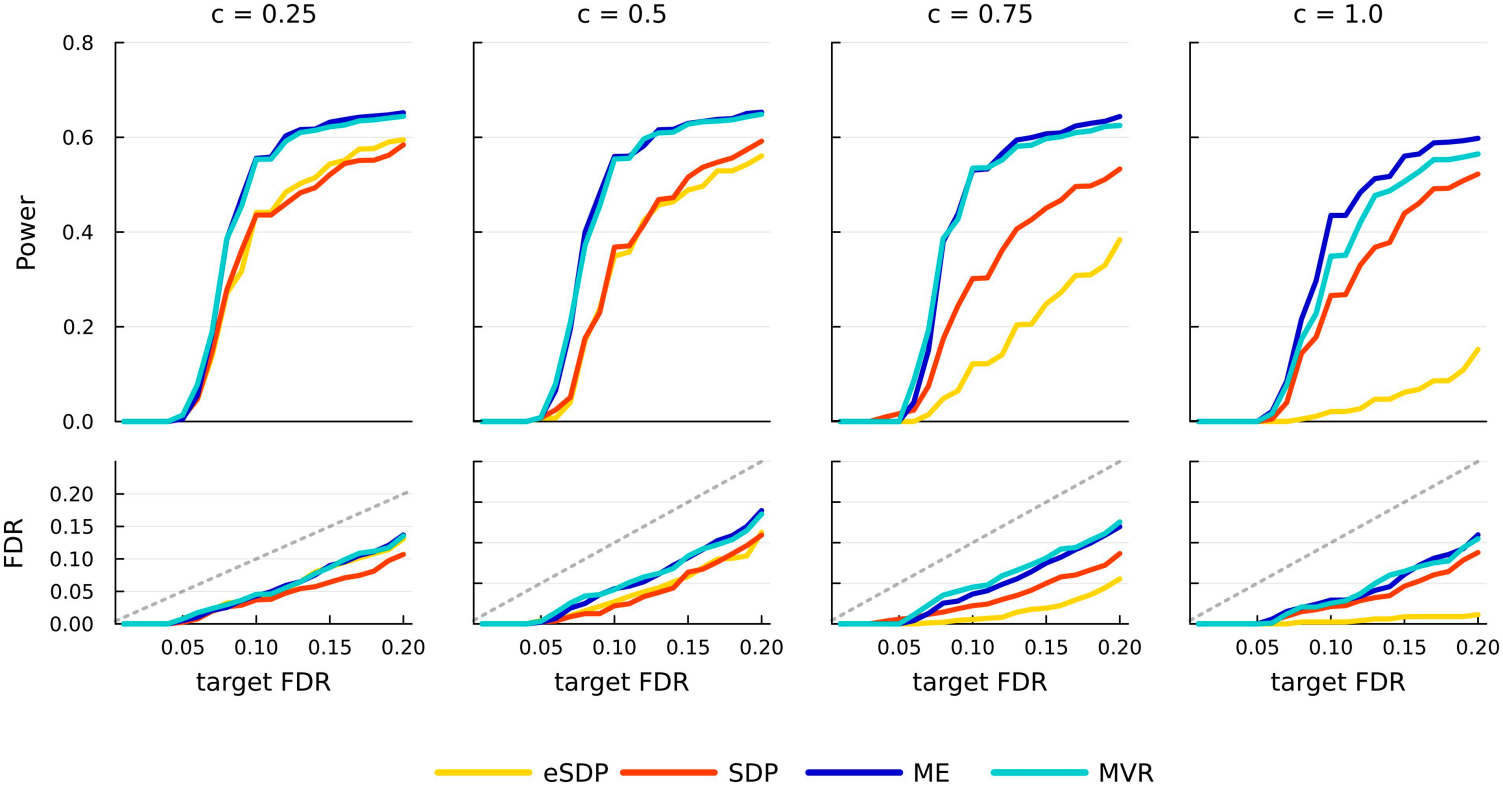
Power/FDR comparison of group knockoffs utilizing conditional independence assumption. Each curve is an average over 100 simulations. There are n=2000 samples and p=8844 genotypes residing on chromosome 22 of UKB. Dashed gray lines indicate the target FDR level. Phenotypes are simulated using real UKB genotypes, while correlation among SNPs is modeled using only LD matrices extracted from the Pan-UKB panel. The case c=1 corresponds to regular group knockoffs.

Figure 2 summarizes power and FDR. In general, ME has the best power, followed by MVR, SDP, and finally eSDP. Utilizing conditional independence offers slightly better power than regular group knockoffs, without sacrificing empirical FDR. In this simulation, group-FDR is controlled for all threshold values *c*, although a separate simulation in Supplementary Fig. S5 shows that c=0.25 could potentially lead to an inflated empirical FDR. The observed power boost is especially beneficial for eSDP constructions, likely because decreasing the number of variables within groups substantially relieves the eSDP constraint to a greater degree than for other methods. Overall, these results suggest the conditional independence assumption can approximate the genetic reality induced by linkage disequilibrium. Given its superior speed and promising empirical performance, we choose a threshold of c=0.5 in our real data analysis of Albuminuria as shown later.

### 6.2 Albuminuria in the UK Biobank

To demonstrate the potential of group knockoffs in analyzing real-world datasets, we applied our methodology to re-analyze an Albuminuria GWAS dataset (Haas *et al.* 2018) with n=382,500 unrelated Europeans and p=11,209,307 SNPs. Elevated level of urine albumin concentration is a hallmark of diabetic kidney disease and is associated with multiple cardiovascular and metabolic diseases. Let us emphasize that the only required inputs are Z-scores and the Pan-UKB linkage disequilibrium (LD) matrices, so knockoff-based conditional independence testing is easily accessible to everyone in the genetics community.

**Figure 3.**
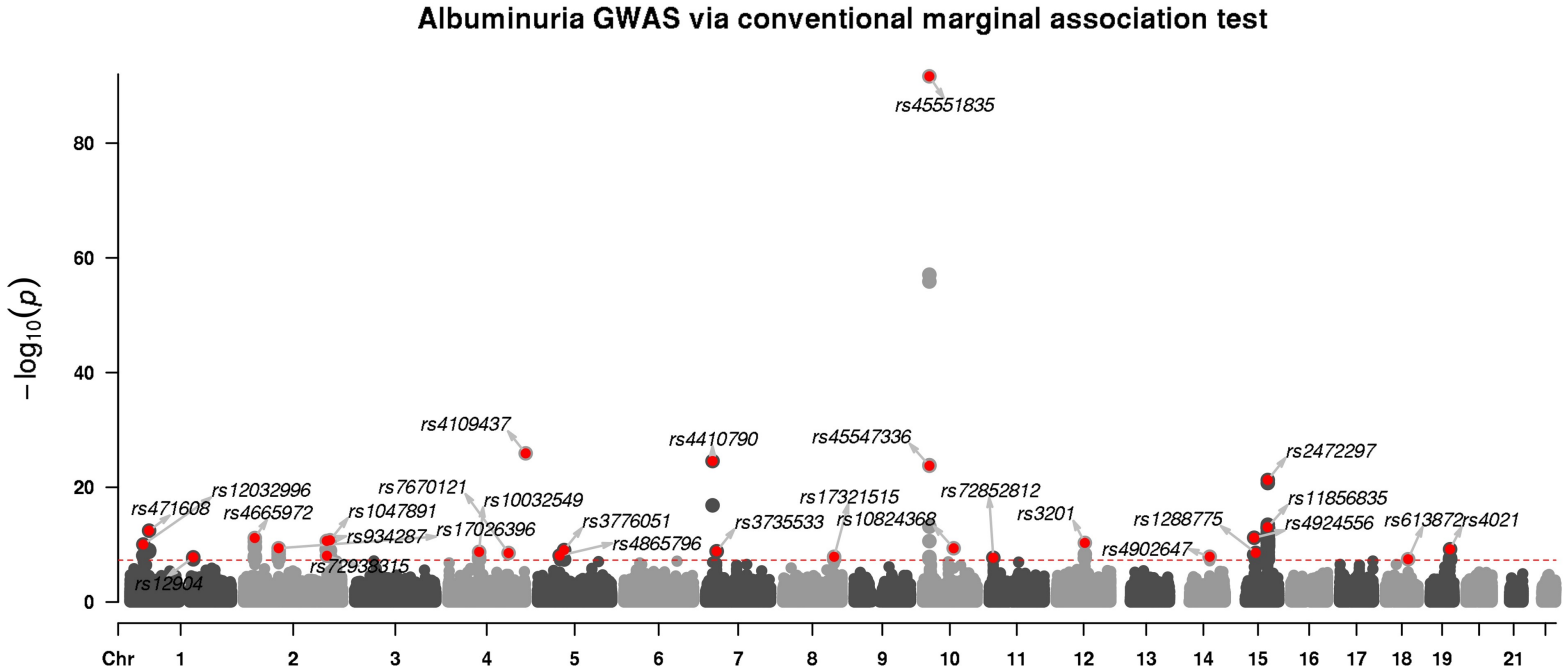
Summary statistics analysis of albuminuria (n=382,500 and p=630,017) using group-knockoff-ghost-Lasso. A conventional marginal-association testing approach is shown in Supplementary Fig. S10. Here, each dot represents a group, where the SNP with the most significant marginal *P*-value within the group is plotted. Only an independent discovery is labeled. A table formatted result is available as Supplementary Table S1.

After matching the study Z-scores to the Pan-UKBB panel, and filtering for genotyped variants with minor allele frequency ≥0.01, we retained p=630,017 Z-scores. We defined groups empirically using average linkage hierarchical clustering with correlation cutoff 0.5. For better speed and power, we use the maximum entropy (ME) solver and exploit the conditional independence assumption by selecting variants within groups such that c=0.5, that is, the key variants explain 50% of the variation within groups. This led to a maximum group size of 4 (see online supplementary material S7.1 for details and summary statistics). The result is visualized in Fig. 3 via the CMplot package (Yin *et al.* 2021).

As a baseline, we utilized the conventional GWAS result in the original paper, based on *t*-tests from marginal linear regression. We note that in Haas *et al.* (2018), the resulting *P*-values are adjusted with the Bonferroni correction, which aims to control family-wise error rate rather than FDR. While it might seem natural to pass these same *P*-values through Benjamini–Hochberg procedure in order to obtain a set of discoveries more directly comparable with the ones of the knockoffs, this strategy is unfortunately not valid. The post-processing procedures of clumping and fine mapping that are needed to obtain interpretable discoveries (Adam *et al.* 2021) invalidate FDR guarantees, as it has been amply documented (Brzyski *et al.* 2017, Sesia *et al.* 2020).

Knockoff-based analysis discovers seven additional independent signals (35 total while controlling FDR at q=0.1) compared to conventional approach, which finds 28 independent signals passing genotype-wide significance threshold of $5\times 10^{-8}$. See Supplementary Table S1 for the full list and Supplementary Table S2 for functional annotations of SNPs missed by traditional GWAS tools. The entire analysis completed in under 3 h, from knockoff construction to a genome-wide Lasso fit. Running genome-wide pseudo-Lasso with 3.6 million variables was the most memory intensive step, requiring roughly 40 GB. An *independent discovery* is defined as the most significant SNP within 1 Mb region, and is highlighted in red. Finally, supplementary Fig. S9 repeats the analysis using the faster but less powerful eSDP knockoffs.

A number of our discoveries have marginal *P*-values close to the genome-wide threshold. Many of these discoveries have been mapped to functional genes that are directly or closely related to the disease phenotype. For instance, rs1077216 ($p=6.8\times 10^{-8}$) was previously reported by an independent study (Teumer *et al.* 2016) for the same trait and rs117287096 ($p=4.6\times 10^{-7}$) is significant with urinary sodium excretion (Pazoki *et al.* 2019). Interestingly, the SNP rs33950747 is missed by a traditional GWAS ($p=7.21\times 10^{-7}$), but it is known to cause a non-synonymous mutation in the NPHS1 gene, which leads to proteinuria (Deltas *et al.* 2023). Other SNPs such as rs12150031 are not previously known, and therefore would be interesting candidates for follow-up studies.

## 7 Discussion

We developed several algorithms for constructing second-order group knockoffs. Group knockoffs promote model selection at the group level, which leads to an improvement in power compared to standard knockoffs when there are highly correlated variables. Of the three group knockoff objectives considered, MVR/ME/SDP, we find that ME tends to exhibit the best power and computational efficiency in simulations with real or artificial data. These algorithms and pipelines are provided as individual open-sourced packages freely available to the scientific community.

Compared to regular model-X knockoffs, group knockoffs require solving a more computationally intensive optimization problem because it requires optimizing significantly more variables. As such, we developed a number of algorithms that are computationally efficient and can flexibly fall back onto less general search spaces when it is not feasible to optimize every variable separately. Furthermore, we also propose a procedure that pre-selects key variables from each group such that the remaining variables are conditionally independent by groups. Group knockoff generation can be done separately for the key and non-key variables, which dramatically reduces the number of parameters for optimization. The validity of this procedure is confirmed theoretically, and we show empirically that it leads to no sacrifice in power or FDR.

In our real-data example, we applied the group knockoff methodology to perform a summary statistics analysis on the albuminuria dataset, where the only required inputs are Z-scores and external LD matrices. Running the pipeline on p=630,017 variables completed in under 3 h, from knockoff construction to a genome-wide Lasso fit. Thus, one can easily conduct knockoff-based conditional independence testing by (1) running standard marginal association testing and (2) inputting the resulting Z-scores into our knockoff pipeline. Recent work (Qi *et al.* 2023) has shown that the first step can rely on commonly used linear mixed models, while the later step can be massively simplified if pre-computed knockoff statistics (e.g. $\Sigma_{i}$ s, $S_{i}$ s,…,etc.) are made widely accessible by storing them on the cloud. In a companion paper (He *et al.* 2024), we try to provide such an ideal resource and conduct more in-depth analyses by applying it to over 60 phenotypes.

Finally, let us discuss a few limitations of the proposed methods and questions worthy of future research. First, the group ME/MVR/SDP algorithms we propose only operate on Gaussian covariates, or in the context of second-order knockoffs, covariates that can be approximated by a normal variable. Next, although selecting group-key variables partially overcomes many computational barriers, further speedup is possible if the covariance matrix can be factored into a low rank model $\Sigma =D+UU^{t}$ where D is a diagonal and U is low rank (Askari *et al.* 2021). One may also consider approaches to pinpoint the causal variant within a discovered group, e.g. by leveraging recent advances in signal localization (Gablenz and Sabatti 2023, Gu *et al.* 2024). Finally, in our summary statistics analysis, it is unclear how knockoffs will respond when samples in a study have ancestral backgrounds that deviate too much from the ethnic backgrounds of the subject used to estimate the LD matrices. To overcome this uncertainty, we recommend analyzing homogeneous populations whose ethnic background matches the subject backgrounds from the LD matrices. As such, we will continue to explore improvements to group knockoffs. Given its promising empirical performance, we recommend it for general use with the understanding that analysts respect its limitations and complement its usage with standard feature selection tools.

## Supplementary Material

btae580_Supplementary_Data

## Data Availability

We implemented the proposed algorithms in an open-sourced Julia (Bezanson *et al.* 2017) package Knockoffs.jl available at https://github.com/biona001/Knockoffs.jl. We also provide R and Python wrappers via the packages knockoffsr and knockoffspy available at https://github.com/biona001/knockoffsr and https://github.com/biona001/knockoffspy. To facilitate downloading and extraction of LD matrices featured in our real data analysis, we developed another software EasyLD.jl available at https://github.com/biona001/EasyLD.jl. Scripts to reproduce our real and simulated experiments are at https://github.com/biona001/ghostknockoff-gwas-reproducibility. The Pan-UKB data were accessed at https://pan.ukbb.broadinstitute.org/docs/hail-format. The UK Biobank data used in supplemental S6.1.2 were accessed under Material Transfer Agreement for UK Biobank Application 27837.
