# Supplementary material for: Second-order group knockoffs with applications to genome-wide association studies
Source: Bioinformatics. 2024 Sep 28;40(10):btae580. doi: 10.1093/bioinformatics/btae580 (PMC11639161; doi:10.1093/bioinformatics/btae580)
Supplement: btae580_Supplementary_Data [file btae580_supplementary_data.pdf]

Supplement to: Second-order group knockoffs with applications to GWAS

Benjamin B. Chu[1], Jiaqi Gu[2], Zhaomeng Chen[3], Tim Morrison[3],
Emmanuel Candès[3,4,*], Zihuai He[2,5,*], Chiara Sabatti[1,3,*]

[1]Department of Biomedical Data Science, Stanford University
[2]Department of Neurology and Neurological Sciences, Stanford University
[3]Department of Statistics, Stanford University
[4]Department of Mathematics, Stanford University
[5]Quantitative Sciences Unit, Department of Medicine, Stanford University

Contents

S1 Fully General Coordinate Descent **2**
S1.1 Fully general coordinate descent for ME (off-diagonal entries) 2
S1.2 Fully general coordinate descent for ME (diagonal entries) 3
S1.3 Fully general coordinate descent for MVR (off-diagonal entries) 4
S1.4 Fully general coordinate descent for MVR (diagonal entries) 5
S1.5 Fully general coordinate descent for SDP 5
S1.6 Feasible region of diagonal entries for general coordinate descent 6
S1.7 Feasible region of off-diagonal entries for general coordinate descent 6
S1.8 Efficiently obtaining needed constants by maintaining Cholesky factors 7
S1.9 Simplifying MVR objective 7
S1.10Initializating coordinate descent algorithms 8
S1.11Declaring convergence 8
S1.12Algorithm summary 8

S2 PCA-based coordinate descent **8**
S2.1 PCA-based Coordinate descent for ME 10
S2.2 PCA-based coordinate descent for MVR. 11
S2.3 PCA-based coordinate descent for SDP 11

S3 Proof of Theorem 1 **12**

S4 Proof of Theorem 2 **12**
S4.1 Sufficient and Necessary Conditions of the minimizer of $L_{\text{ME}}(\mathbf{S})$ 13

[*]Joint corresponding authors

S5 Heuristic strategy to identify groups and group-key variables to approximately achieve conditional independence **15**
S5.1 Defining groups by Hierarchical clustering . . . 15
S5.2 Defining groups by Interpolative Decomposition (ID) . . . 15
S5.2.1 Interpolative decomposition for covariance matrices . . . 16
S5.3 Selecting group-key variables to exploit conditional independence . . . 16

S6 Practical strategy for estimating $\hat{\mu}$ and $\hat{\Sigma}$ **17**

S7 GhostKnockoff pipeline on the Pan-UKB panel **18**
S7.1 Summary statistics on Pan-UKB matrices . . . 18
S7.2 Regularizations applied to Pan-UKB LD matrices . . . 18
S7.3 Visualization of group knockoff exchangeability with different thresholds for selecting group-key variables . . . 19
S7.4 Tuning Lasso hyperparameter without individual level data . . . 19
S7.5 Summary of Ghost Knockoff pipeline . . . 21

S8 Additional simulations **21**
S8.1 The advantage of group-based inference . . . 21
S8.2 Simulation details for section 6.1.1 in main text . . . 22
S8.3 Additional simulations using the Pan-UKB panel . . . 23
S8.4 Additional runtime comparisons of group knockoff solver vs utilization of conditional indepenence . . . 25
S8.5 Marginal correlation as feature importance statistics . . . 25
S8.6 Distribution of S_{ij} and minimum eigenvalues of $\mathbf{G_S}$ . . . 26

S9 Albuminuria GWAS - in depth analysis **27**
S9.1 Result using eSDP knockoffs . . . 27
S9.2 Manhattan plot for Marginal association test of Albuminuria . . . 27
S9.3 Full list of discoveries from Albuminuria GWAS . . . 27
S9.4 Functional annotations for additionally discovered SNPs . . . 27

S1 Fully General Coordinate Descent

In general coordinate descent, we update $\mathbf{S}$ with

$$S_{ij}^{new} = S_{ij} + \delta.$$

Here, we fill in algorithmic details for the general coordinate descent algorithm featured in Section 3 of the main paper. Details for PCA updates are provided in section S2. A summary is provided as Algorithm (A1), which is a more detailed version of Algorithm 1 in the main text.

S1.1 Fully general coordinate descent for ME (off-diagonal entries)

The objective for maximum entropy knockoffs can be simplified to maximizing the following (Gimenez and Zou 2019)

$$\max_{\mathbf{S}} \log\det\left(\frac{m+1}{m}\mathbf{\Sigma} - \mathbf{S}\right) + m\log\det(\mathbf{S}) \quad \text{subject to} \begin{cases} \frac{m+1}{m}\mathbf{\Sigma} - \mathbf{S} \succeq 0 \\ \mathbf{S} \succeq 0 \end{cases}.$$

Consider updating $S_{ij}^{new} = S_{ij} + \delta$ where $i \neq j$. Our goal is to compute δ. First note that by symmetry, we must also set $S_{ji}^{new} = S_{ij}^{new}$. Let $\mathbf{e}_i$ be the ith basis vector and $\mathbf{H} = \mathbf{e}_i \mathbf{e}_j^t + \mathbf{e}_j \mathbf{e}_i^t$ be a $p \times p$ matrix that is zero everywhere except $H_{ij} = H_{ji} = 1$. Thus, we have $\mathbf{S}^{new} = \mathbf{S} + \delta \mathbf{H}$. If we define $\mathbf{D} = \frac{m+1}{m}\boldsymbol{\Sigma} - \mathbf{S}$, the objective becomes

$$\log\det(\mathbf{D} - \delta\mathbf{H}) + m \log\det\left(\mathbf{S} + \delta\mathbf{H}\right).$$

Letting $\mathbf{H} = \mathbf{U}\mathbf{V}$, where $\mathbf{U} = \begin{bmatrix}\mathbf{e}_i & \mathbf{e}_j\end{bmatrix} \in \mathbb{R}^{p\times 2}$ and $\mathbf{V} = \begin{bmatrix}\mathbf{e}_j^t \\ \mathbf{e}_i^t\end{bmatrix} \in \mathbb{R}^{2\times p}$, the objective can be simplified by the matrix determinant lemma

$$\begin{aligned}
\log\det(\mathbf{D} - \delta\mathbf{H}) &= \log\left(\det(\mathbf{I}_2 - \delta\mathbf{V}\mathbf{D}^{-1}\mathbf{U})\det(\mathbf{D})\right) = \log\det(\mathbf{I}_2 - \delta\mathbf{V}\mathbf{D}^{-1}\mathbf{U}) + c, \\
\log\det(\mathbf{S} + \delta\mathbf{H}) &= \log\left(\det(\mathbf{I}_2 + \delta\mathbf{V}\mathbf{S}^{-1}\mathbf{U})\det(\mathbf{S})\right) = \log\det(\mathbf{I}_2 + \delta\mathbf{V}\mathbf{S}^{-1}\mathbf{U}) + c.
\end{aligned}$$

Due to the special structures of $\mathbf{U}$ and $\mathbf{V}$, we can explicitly compute

$$\mathbf{V}\mathbf{D}^{-1}\mathbf{U} = \begin{bmatrix} a_{ji} & a_{jj} \\ a_{ii} & a_{ij}\end{bmatrix}, \qquad \mathbf{V}\mathbf{S}^{-1}\mathbf{U} = \begin{bmatrix} b_{ji} & b_{jj} \\ b_{ii} & b_{ij}\end{bmatrix},$$

where

$$\begin{aligned}
a_{ij} &= \mathbf{e}_i^t\mathbf{D}^{-1}\mathbf{e}_j, \quad a_{ii} = \mathbf{e}_i^t\mathbf{D}^{-1}\mathbf{e}_i, \quad a_{jj} = \mathbf{e}_j^t\mathbf{D}^{-1}\mathbf{e}_j, \\
b_{ij} &= \mathbf{e}_i^t\mathbf{S}^{-1}\mathbf{e}_j, \quad b_{ii} = \mathbf{e}_i^t\mathbf{S}^{-1}\mathbf{e}_i, \quad b_{jj} = \mathbf{e}_j^t\mathbf{S}^{-1}\mathbf{e}_j.
\end{aligned}$$

Thus, evaluating the 2×2 determinants, the objective becomes

$$g(\delta) = \log\left((1 - \delta a_{ij})^2 - \delta^2 a_{ii}a_{jj}\right) + m\log\left((1 + \delta b_{ij})^2 - \delta^2 b_{jj}b_{ii}\right). \tag{S.1}$$

This is a scalar-valued function with scalar inputs, so it is easy to optimize within an interval that defines the feasible region of δ. In our software, we use Brent's method implemented in `Optim.jl` to solve this problem. Note that δ needs to reside within an interval to ensure the positive definite constraints are satisfied. This interval is derived in sections S1.6 and S1.7, and we discuss how to efficiently obtain constants $a_{ij}, a_{ii}, a_{jj}, b_{ij}, b_{ii}, b_{jj}$ in section S1.8.

S1.2 Fully general coordinate descent for ME (diagonal entries)

Now we consider optimizing the diagonal entries, i.e. we want to find δ for the update $S_{jj}^{new} = S_{jj} + \delta\mathbf{e}_j\mathbf{e}_j^t$. Again letting $\mathbf{D} = \frac{m+1}{m}\boldsymbol{\Sigma} - \mathbf{S}$, the objective becomes

$$\begin{aligned}
g(\delta) &= \ln\det(\mathbf{D} - \delta\mathbf{e}_j\mathbf{e}_j^t) + m\ln\det(\mathbf{S} + \delta\mathbf{e}_j\mathbf{e}_j^t) \\
&= \ln\left[(1 - \delta\mathbf{e}_j\mathbf{D}^{-1}\mathbf{e}_j)\det(\mathbf{D})\right] + m\ln\left[(1 + \delta\mathbf{e}_j^t\mathbf{S}^{-1}\mathbf{e}_j)\det(\mathbf{S})\right] \\
&\propto \ln(1 - \delta\mathbf{e}_j\mathbf{D}^{-1}\mathbf{e}_j) + m\ln(1 + \delta\mathbf{e}_j\mathbf{S}^{-1}\mathbf{e}_j)
\end{aligned}$$

where the second equality follows from the matrix determinant lemma. The first order optimality condition states

$$\frac{d}{d\delta}g(\delta) = \frac{-\mathbf{e}_j\mathbf{D}^{-1}\mathbf{e}_j}{1 - \delta\mathbf{e}_j\mathbf{D}^{-1}\mathbf{e}_j} + \frac{m\mathbf{e}_j\mathbf{S}^{-1}\mathbf{e}_j}{1 + \delta\mathbf{e}_j\mathbf{S}^{-1}\mathbf{e}_j} = 0.$$

In the notation for computing off-diagonal entries, we have

$$\delta = \frac{m\mathbf{e}_j\mathbf{S}^{-1}\mathbf{e}_j - \mathbf{e}_j\mathbf{D}^{-1}\mathbf{e}_j}{(m+1)\mathbf{e}_j\mathbf{S}^{-1}\mathbf{e}_j\mathbf{e}_j\mathbf{D}^{-1}\mathbf{e}_j} = \frac{mb_{jj} - a_{jj}}{(m+1)b_{jj}a_{jj}}. \tag{S.2}$$

S1.3 Fully general coordinate descent for MVR (off-diagonal entries)

In section S1.9 we show that minimum variance-based reconstructability (MVR) knockoffs solve the problem

$$\min_{\mathbf{S}} \; m^2 \operatorname{tr}(\mathbf{S}^{-1}) + \operatorname{tr}\left(\frac{m+1}{m}\boldsymbol{\Sigma} - \mathbf{S}\right)^{-1} \qquad \text{subject to } \begin{cases} \frac{m+1}{m}\boldsymbol{\Sigma} - \mathbf{S} \succeq 0 \\ \mathbf{S} \succeq 0 \end{cases}.$$

Again we consider updating $S_{ij}^{new} = S_{ij} + \delta$ where $i \neq j$. Let $\mathbf{D} = \frac{m+1}{m}\boldsymbol{\Sigma} - \mathbf{S}$ and $\mathbf{H}$ be the $p \times p$ matrix that is zero everywhere except $H_{ij} = H_{ji} = 1$. The objective becomes

$$m^2 \operatorname{tr}(\mathbf{S} + \delta\mathbf{H})^{-1} + \operatorname{tr}(\mathbf{D} - \delta\mathbf{H})^{-1}.$$

Since $\mathbf{H} = \mathbf{U}\mathbf{V}$, where $\mathbf{U} = \begin{bmatrix}\mathbf{e}_i & \mathbf{e}_j\end{bmatrix} \in \mathbb{R}^{p\times 2}$ and $\mathbf{V} = \begin{bmatrix}\mathbf{e}_j^t \\ \mathbf{e}_i^t\end{bmatrix} \in \mathbb{R}^{2\times p}$, Woodbury's formula gives

$$\begin{aligned}
\operatorname{tr}(\mathbf{D} - \delta\mathbf{H})^{-1} &= \operatorname{tr}(\mathbf{D} - \delta\mathbf{U}\mathbf{V})^{-1} \\
&= \operatorname{tr}\left(\mathbf{D}^{-1} + \delta\mathbf{D}^{-1}\mathbf{U}(\mathbf{I}_2 - \delta\mathbf{V}\mathbf{D}^{-1}\mathbf{U})^{-1}\mathbf{V}\mathbf{D}^{-1}\right) \\
&= \delta \operatorname{tr}\left(\mathbf{D}^{-1}\mathbf{U}(\mathbf{I}_2 - \delta\mathbf{V}\mathbf{D}^{-1}\mathbf{U})^{-1}\mathbf{V}\mathbf{D}^{-1}\right) + c \\
&= \delta \operatorname{tr}\left(\mathbf{V}\mathbf{D}^{-2}\mathbf{U}(\mathbf{I}_2 - \delta\mathbf{V}\mathbf{D}^{-1}\mathbf{U})^{-1}\right) + c, \\
\operatorname{tr}(\mathbf{S} + \delta\mathbf{H})^{-1} &= -\delta \operatorname{tr}\left(\mathbf{V}\mathbf{S}^{-2}\mathbf{U}(\mathbf{I}_2 + \delta\mathbf{V}\mathbf{S}^{-1}\mathbf{U})^{-1}\right)
\end{aligned}$$

We already have explicit expressions for $\mathbf{V}\mathbf{S}^{-1}\mathbf{U}$ and $\mathbf{V}\mathbf{D}^{-1}\mathbf{U}$, thus

$$\begin{aligned}
(\mathbf{I}_2 - \delta\mathbf{V}\mathbf{D}^{-1}\mathbf{U})^{-1} &= \begin{bmatrix} 1-\delta a_{ij} & -\delta a_{jj} \\ -\delta a_{ii} & 1-\delta a_{ij}\end{bmatrix}^{-1} = \frac{1}{(1-\delta a_{ij})^2 - \delta^2 a_{ii}a_{jj}} \begin{bmatrix} 1-\delta a_{ij} & \delta a_{jj} \\ \delta a_{ii} & 1-\delta a_{ij}\end{bmatrix}, \\
(\mathbf{I}_2 + \delta\mathbf{V}\mathbf{S}^{-1}\mathbf{U})^{-1} &= \begin{bmatrix} 1+\delta b_{ij} & \delta b_{jj} \\ \delta b_{ii} & 1+\delta b_{ij}\end{bmatrix}^{-1} = \frac{1}{(1+\delta b_{ij})^2 - \delta^2 b_{ii}b_{jj}} \begin{bmatrix} 1+\delta b_{ij} & -\delta b_{jj} \\ -\delta b_{ii} & 1+\delta b_{ij}\end{bmatrix}.
\end{aligned}$$

Similarly, $\mathbf{V}\mathbf{S}^{-2}\mathbf{U}$ and $\mathbf{V}\mathbf{D}^{-2}\mathbf{U}$ can be computed as

$$\mathbf{V}\mathbf{S}^{-2}\mathbf{U} = \begin{bmatrix} c_{ji} & c_{jj} \\ c_{ii} & c_{ij}\end{bmatrix}, \qquad \mathbf{V}\mathbf{D}^{-2}\mathbf{U} = \begin{bmatrix} d_{ji} & d_{jj} \\ d_{ii} & d_{ij}\end{bmatrix},$$

where

$$\begin{aligned}
c_{ij} = \mathbf{e}_i^t\mathbf{S}^{-2}\mathbf{e}_j, \quad c_{ii} = \mathbf{e}_i^t\mathbf{S}^{-2}\mathbf{e}_i, \quad c_{jj} = \mathbf{e}_j^t\mathbf{S}^{-2}\mathbf{e}_j, \\
d_{ij} = \mathbf{e}_i^t\mathbf{D}^{-2}\mathbf{e}_j, \quad d_{ii} = \mathbf{e}_i^t\mathbf{D}^{-2}\mathbf{e}_i, \quad d_{jj} = \mathbf{e}_j^t\mathbf{D}^{-2}\mathbf{e}_j.
\end{aligned}$$

The objective is therefore a scalar function of δ

$$\begin{aligned}
g(\delta) &= \frac{-m^2\delta}{(1+\delta b_{ij})^2 - \delta^2 b_{ii}b_{jj}} \operatorname{tr}\left(\begin{bmatrix} c_{ij} & c_{jj} \\ c_{ii} & c_{ij}\end{bmatrix}\begin{bmatrix} 1+\delta b_{ij} & -\delta b_{jj} \\ -\delta b_{ii} & 1+\delta b_{ij}\end{bmatrix}\right) + \\
&\quad \frac{\delta}{(1-\delta a_{ij})^2 - \delta^2 a_{ii}a_{jj}} \operatorname{tr}\left(\begin{bmatrix} d_{ij} & d_{jj} \\ d_{ii} & d_{ij}\end{bmatrix}\begin{bmatrix} 1-\delta a_{ij} & \delta a_{jj} \\ \delta a_{ii} & 1-\delta a_{jj}\end{bmatrix}\right) \\
&= \frac{-m^2\delta\left\{(c_{ij}b_{ij} - c_{jj}b_{ii} - c_{ii}b_{jj} + c_{ij}b_{ij})\delta + 2c_{ij}\right\}}{(1+\delta b_{ij})^2 - \delta^2 b_{ii}b_{jj}} + \\
&\quad \frac{\delta\left\{(-d_{ij}a_{ij} + d_{jj}a_{ii} + d_{ii}a_{jj} - d_{ij}a_{ij})\delta + 2d_{ij}\right\}}{(1-\delta a_{ij})^2 - \delta^2 a_{ii}a_{jj}}. \qquad \text{(S.3)}
\end{aligned}$$

If all the constants $a_{ii}, a_{ij}, a_{jj}, b_{ii}, b_{ij}, b_{jj}, c_{ii}, c_{ij}, c_{jj}, d_{ii}, d_{ij}, d_{jj}$ are known, the objective can be minimized using Brent's method similar to the maximum entropy case. If we hold the Cholesky factors $\mathbf{D} = \mathbf{L}\mathbf{L}^t$ and $\mathbf{S} = \mathbf{C}\mathbf{C}^t$, then the first six of these constants can be evaluated in the same way as in S1.8. To evaluate the other six constants, e.g. d_{ij}, note that

$$d_{ij} = \mathbf{e}_i^t \mathbf{D}^{-2} \mathbf{e}_j = \mathbf{e}_i^t (\mathbf{L}\mathbf{L}^t)^{-1} (\mathbf{L}\mathbf{L}^t)^{-1} \mathbf{e}_j \equiv \mathbf{u}^t \mathbf{v}.$$

Here $\mathbf{u}, \mathbf{v}$ can be obtained by noting that $\mathbf{u} = (\mathbf{L}\mathbf{L}^t)^{-1}\mathbf{e}_i \iff \mathbf{L}\mathbf{L}^t\mathbf{u} = \mathbf{e}_i$ and using forward-backward substitution twice; that is, first solve for $\mathbf{y}$ in $\mathbf{L}\mathbf{y} = \mathbf{e}_i$ and then for $\mathbf{u}$ in $\mathbf{L}^t\mathbf{u} = \mathbf{y}$.

Finally, we need to ensure that the proposed δ is feasible. Because the PSD constraint is the same as the maximum entropy case, the feasible region is already derived in sections S1.6 and S1.7. Once we update $S_{ij}^{new} = S_{ij} + \delta$ and $S_{ji}^{new} = S_{ji} + \delta$, we perform a rank-2 update to maintain Cholesky equalities $\mathbf{L}_{new}\mathbf{L}_{new}^t = \frac{m+1}{m}\mathbf{\Sigma} - \mathbf{S}_{new}$ and $\mathbf{C}_{new}\mathbf{C}_{new}^t = \mathbf{S}_{new}$. Again, this can be achieved with rank-1 updates in equation (S.8).

S1.4 Fully general coordinate descent for MVR (diagonal entries)

Now we consider optimizing the diagonal entries, i.e. we want to find δ for the update $S_{jj}^{new} = S_{jj} + \delta \mathbf{e}_j \mathbf{e}_j^t$. Again letting $\mathbf{D} = \frac{m+1}{m}\mathbf{\Sigma} - \mathbf{S}$, the objective becomes

$$\begin{aligned}
g(\mathbf{S} + \delta \mathbf{e}_j \mathbf{e}_j^t) &= m^2 \operatorname{tr}[(\mathbf{S} + \delta \mathbf{e}_j \mathbf{e}_j^t)^{-1}] + \operatorname{tr}[(\mathbf{D} - \delta \mathbf{e}_j \mathbf{e}_j^t)^{-1}] \\
&= m^2 \operatorname{tr}\left[\mathbf{S}^{-1} - \frac{\delta \mathbf{S}^{-1}\mathbf{e}_j\mathbf{e}_j^t\mathbf{S}^{-1}}{1 + \delta \mathbf{e}_j^t \mathbf{S}^{-1}\mathbf{e}_j}\right] + \operatorname{tr}\left[\mathbf{D}^{-1} + \frac{\delta \mathbf{D}^{-1}\mathbf{e}_j\mathbf{e}_j^t\mathbf{D}^{-1}}{1 - \delta \mathbf{e}_j^t \mathbf{D}^{-1}\mathbf{e}_j}\right] \quad \text{(Sherman-Morrison)} \\
&= \frac{-m^2\delta}{1 + \delta \mathbf{e}_j^t \mathbf{S}^{-1}\mathbf{e}_j} \operatorname{tr}(\mathbf{S}^{-1}\mathbf{e}_j\mathbf{e}_j^t\mathbf{S}^{-1}) + \frac{\delta}{1 - \delta \mathbf{e}_j^t \mathbf{D}^{-1}\mathbf{e}_j} \operatorname{tr}(\mathbf{D}^{-1}\mathbf{e}_j\mathbf{e}_j^t\mathbf{D}^{-1}) + m^2 \operatorname{tr}(\mathbf{S}^{-1}) + \operatorname{tr}(\mathbf{D}^{-1}) \\
&= \frac{-m^2 \mathbf{e}_j^t \mathbf{S}^{-2}\mathbf{e}_j \delta}{1 + \delta \mathbf{e}_j^t \mathbf{S}^{-1}\mathbf{e}_j} + \frac{\delta \mathbf{e}_j^t \mathbf{D}^{-2}\mathbf{e}_j}{1 - \delta \mathbf{e}_j^t \mathbf{D}^{-1}\mathbf{e}_j} + m^2 \operatorname{tr}(\mathbf{S}^{-1}) + \operatorname{tr}(\mathbf{D}^{-1}) \quad \text{(cyclic property of trace)} \\
&\equiv \frac{-m^2\delta c_{jj}}{1 + \delta b_{jj}} + \frac{\delta d_{jj}}{1 - \delta a_{jj}} + g(\mathbf{S}),
\end{aligned}$$

with the convention that $a_{jj} = \mathbf{e}_j^t \mathbf{D}^{-1}\mathbf{e}_j, b_{jj} = \mathbf{e}_j^t \mathbf{S}^{-1}\mathbf{e}_j, c_{jj} = \mathbf{e}_j^t \mathbf{S}^{-2}\mathbf{e}_j$, and $d_{jj} = \mathbf{e}_j^t \mathbf{D}^{-2}\mathbf{e}_j$. The first-order optimality condition states

$$\begin{aligned}
0 &= \frac{-(1 + \delta b_{jj}) m^2 c_{jj} + \delta m^2 c_{jj} b_{jj}}{(1 + \delta b_{jj})^2} + \frac{(1 - \delta a_{jj}) d_{jj} + \delta d_{jj} a_{jj}}{(1 - \delta a_{jj})^2} \\
&= -m^2 c_{jj}(1 - \delta a_{jj})^2 + (1 + \delta b_{jj})^2 d_{jj} \\
&= \delta^2(-a_{jj}^2 m^2 c_{jj} + b_{jj}^2 d_{jj}) + \delta(2a_{jj}m^2 c_{jj} + 2b_{jj}d_{jj}) - m^2 c_{jj} + d_{jj}.
\end{aligned}$$

We apply the quadratic formula to find the roots of this objective, and enforce the boundary condition $-1/b_{jj} \leq \delta \leq 1/a_{jj}$ derived in section S1.6.

S1.5 Fully general coordinate descent for SDP

Following the same definition of $\mathbf{H}$ as MVR/ME case, the SDP objective is

$$\min_S \sum_{g \in G} \frac{1}{|\mathcal{A}_g|^2} \sum_{i,j \in \mathcal{A}_g} |\Sigma_{ij}^{(g)} - S_{ij}^{(g)} - \delta H_{ij}|.$$

Only two entries of the objective depend on δ:

$$|\Sigma_{ij} - S_{ij} - \delta| + |\Sigma_{ji} - S_{ji} - \delta| = 2|\Sigma_{ij} - S_{ij} - \delta|.$$

Computing the feasible region of $\delta \in [a, b]$ according to sections S1.6 and S1.7, the solution is

$$\delta = \begin{cases} \Sigma_{ij} - S_{ij} & \Sigma_{ij} - S_{ij} \in [a, b] \\ a & \Sigma_{ij} - S_{ij} \leq a \\ b & \Sigma_{ij} - S_{ij} \geq b \end{cases}. \tag{S.4}$$

S1.6 Feasible region of diagonal entries for general coordinate descent

What is the feasible region of δ? To satisfy the PSD constraints, we must choose δ so that $\mathbf{D} - \delta \mathbf{e}_j \mathbf{e}_j^t \succeq 0$ and $\mathbf{S} + \delta \mathbf{e}_j \mathbf{e}_j^t \succeq 0$. Applying the matrix determinant lemma again,

$$\begin{aligned} \det(\mathbf{D} - \delta \mathbf{e}_j \mathbf{e}_j^t) \geq 0 \quad &\Longleftrightarrow \quad (1 - \delta \mathbf{e}_j \mathbf{D}^{-1} \mathbf{e}_j) \det(\mathbf{D}) \geq 0, \\ \det(\mathbf{S} + \delta \mathbf{e}_j \mathbf{e}_j^t) \geq 0 \quad &\Longleftrightarrow \quad (1 + \delta \mathbf{e}_j \mathbf{S}^{-1} \mathbf{e}_j) \det(\mathbf{S}) \geq 0. \end{aligned}$$

Since $\mathbf{D}$ and $\mathbf{S}$ are positive definite, we must have

$$\frac{1}{\mathbf{e}_j^t \mathbf{D}^{-1} \mathbf{e}_j} \geq \delta \geq \frac{-1}{\mathbf{e}_j^t \mathbf{S}^{-1} \mathbf{e}_j} \quad \Longleftrightarrow \quad \frac{1}{a_{jj}} \geq \delta \geq \frac{-1}{b_{jj}}. \tag{S.5}$$

S1.7 Feasible region of off-diagonal entries for general coordinate descent

δ must satisfy $\mathbf{D} - \delta \mathbf{H} \succeq 0$ and $\mathbf{S} + \delta \mathbf{H} \succeq 0$. By convexity and continuity, these conditions define a closed interval of feasible values of δ, with the endpoints satisfying either $\det(\mathbf{D} - \delta \mathbf{H}) = 0$ or $\det(\mathbf{S} + \delta \mathbf{H}) = 0$. In the first case, $\det(\mathbf{D} - \delta \mathbf{H}) = (1 - \delta a_{ij})^2 - \delta^2 a_{ii} a_{jj}$, which is a quadratic function. Let s_1, s_2 be its roots and d_1, d_2 be the roots of $\det(\mathbf{S} + \delta \mathbf{H})$. Then

$$\begin{aligned} s_1 &= \frac{a_{ij} - \sqrt{a_{ii} a_{jj}}}{a_{ij}^2 - a_{ii} a_{jj}}, \quad & s_2 &= \frac{a_{ij} + \sqrt{a_{ii} a_{jj}}}{a_{ij}^2 - a_{ii} a_{jj}}, \\ d_1 &= \frac{-b_{ij} - \sqrt{b_{ii} b_{jj}}}{b_{ij}^2 - b_{ii} b_{jj}}, \quad & d_2 &= \frac{-b_{ij} + \sqrt{b_{ii} b_{jj}}}{b_{ij}^2 - b_{ii} b_{jj}}, \end{aligned}$$

and the feasible region is defined by

$$\max\{s_1, d_1\} \leq \delta \leq \min\{s_2, d_2\}. \tag{S.6}$$

In practice, we often need more stringent lower and upper bounds for computational reasons. Specifically, note that the updates $\mathbf{S} + \delta \mathbf{H}$ and $\mathbf{D} - \delta \mathbf{H}$ require us to maintain Cholesky equalities in equation (S.8), which is achieved via two separate rank-1 updates. Because we perform these actions sequentially, it is possible to violate positive definiteness in an intermediate step even though the overall update does not. Thus, we in fact need all of the following

$$\begin{cases} \mathbf{D} - \frac{\delta}{2} \left((\mathbf{e}_i + \mathbf{e}_j)(\mathbf{e}_i + \mathbf{e}_j)^t \right) \succeq 0 & \text{(first rank-1 update to } \mathbf{L}) \\ \mathbf{D} - \delta(\mathbf{e}_i \mathbf{e}_j^t + \mathbf{e}_j \mathbf{e}_i^t) \succeq 0 & \text{(final rank 1 update to } \mathbf{L}\text{, feasible region derived in (S.6))} \end{cases},$$

$$\begin{cases} \mathbf{S} + \frac{\delta}{2} \left((\mathbf{e}_i + \mathbf{e}_j)(\mathbf{e}_i + \mathbf{e}_j)^t \right) \succeq 0 & \text{(first rank-1 update to } \mathbf{C}) \\ \mathbf{S} + \delta(\mathbf{e}_i \mathbf{e}_j^t + \mathbf{e}_j \mathbf{e}_i^t) \succeq 0 & \text{(final rank 1 update to } \mathbf{C}\text{, feasible region derived in (S.6))} \end{cases}.$$

Applying the matrix determinant lemma to the first terms of these equations,

$$\det(\mathbf{D} - \frac{\delta}{2}(\mathbf{e}_i + \mathbf{e}_j)(\mathbf{e}_i + \mathbf{e}_j)^t) \geq 0 \iff (1 - \frac{\delta}{2}(\mathbf{e}_i + \mathbf{e}_j)^t \mathbf{D}^{-1}(\mathbf{e}_i + \mathbf{e}_j)) \det(\mathbf{D}) \geq 0,$$
$$\det(\mathbf{S} + \frac{\delta}{2}(\mathbf{e}_i + \mathbf{e}_j)(\mathbf{e}_i + \mathbf{e}_j)^t) \geq 0 \iff (1 + \frac{\delta}{2}(\mathbf{e}_i + \mathbf{e}_j)^t \mathbf{S}^{-1}(\mathbf{e}_i + \mathbf{e}_j)) \det(\mathbf{S}) \geq 0.$$

Solving for δ, a different lower/upper bound emerges:

$$\frac{2}{\mathbf{e}_i^t \mathbf{D}^{-1}\mathbf{e}_i + \mathbf{e}_i \mathbf{D}^{-1}\mathbf{e}_j + \mathbf{e}_j^t \mathbf{D}^{-1}\mathbf{e}_i + \mathbf{e}_j \mathbf{D}^{-1}\mathbf{e}_j} \geq \delta \geq \frac{-2}{\mathbf{e}_i^t \mathbf{S}^{-1}\mathbf{e}_i + \mathbf{e}_i \mathbf{S}^{-1}\mathbf{e}_j + \mathbf{e}_j^t \mathbf{S}^{-1}\mathbf{e}_i + \mathbf{e}_j \mathbf{S}^{-1}\mathbf{e}_j}.$$

Thus, in the notation of the objective, δ must additionally satisfy the (computational) feasible region

$$\frac{2}{a_{ii} + 2a_{ij} + a_{jj}} \geq \delta \geq \frac{-2}{b_{ii} + 2b_{ij} + b_{jj}}. \tag{S.7}$$

S1.8 Efficiently obtaining needed constants by maintaining Cholesky factors

To efficiently evaluate constants such as $\mathbf{e}_i^t \mathbf{D}^{-1}\mathbf{e}_j$ and $\mathbf{e}_i^t \mathbf{S}^{-1}\mathbf{e}_j$, the natural way is to precompute and constantly update $\mathbf{D}^{-1}, \mathbf{S}^{-1}$ via Woodbury formulas. However, past work (Spector and Janson 2022; Askari et al. 2021) suggests that these low-rank updates are numerically unstable. The typical approach is to maintain two Cholesky decompositions $\mathbf{D} = \mathbf{L}\mathbf{L}^t$ and $\mathbf{S} = \mathbf{C}\mathbf{C}^t$, and proceed to extract necessary constants as described in section 3 of the main text.

After updating $S_{ij}^{new} = S_{ij} + \delta$ and $S_{ji}^{new} = S_{ij}^{new}$, we need to update the Cholesky factors $\mathbf{C}$ and $\mathbf{L}$ to maintain equalities $\mathbf{L}_{new}\mathbf{L}_{new}^t = \frac{m+1}{m}\mathbf{\Sigma} - \mathbf{S}_{new}$ and $\mathbf{C}_{new}\mathbf{C}_{new}^t = \mathbf{S}_{new}$. In general, if we have $\mathbf{L}\mathbf{L}^t = \mathbf{A}$, we can obtain the Cholesky factor of $\mathbf{A} + \mathbf{w}\mathbf{w}^t$ (i.e. a rank-1 update from $\mathbf{A}$) in $\mathcal{O}(p^2)$ time. In light of this, lets write the equality we wish to maintain as:

$$\begin{aligned}
\mathbf{L}_{new}\mathbf{L}_{new}^t &= \frac{m+1}{m}\mathbf{\Sigma} - \mathbf{S} - \delta(\mathbf{e}_i\mathbf{e}_j^t + \mathbf{e}_j\mathbf{e}_i^t) \\
&= \frac{m+1}{m}\mathbf{\Sigma} - \mathbf{S} - \frac{\delta}{2}\left((\mathbf{e}_i + \mathbf{e}_j)(\mathbf{e}_i + \mathbf{e}_j)^t - (\mathbf{e}_i - \mathbf{e}_j)(\mathbf{e}_i - \mathbf{e}_j)^t\right), \qquad \text{(S.8)} \\
\mathbf{C}_{new}\mathbf{C}_{new}^t &= \mathbf{S} + \delta(\mathbf{e}_i\mathbf{e}_j^t + \mathbf{e}_j\mathbf{e}_i^t) = \mathbf{S} + \frac{\delta}{2}\left((\mathbf{e}_i + \mathbf{e}_j)(\mathbf{e}_i + \mathbf{e}_j)^t - (\mathbf{e}_i - \mathbf{e}_j)(\mathbf{e}_i - \mathbf{e}_j)^t\right).
\end{aligned}$$

Thus, the required rank-2 update can be achieved by first rank-1 updating using $\mathbf{w} = \sqrt{\frac{\delta}{2}}(\mathbf{e}_i + \mathbf{e}_j)$ and then perform rank-1 downdate via $\mathbf{w} = \sqrt{\frac{\delta}{2}}(\mathbf{e}_i - \mathbf{e}_j)$.

S1.9 Simplifying MVR objective

When generating m multiple knockoffs, the MVR objective (Spector and Janson 2022) is

$$\operatorname{tr}(\mathbf{G}_{\mathbf{S}}^{-1}) \equiv \operatorname{tr}\begin{pmatrix} \mathbf{\Sigma} & \mathbf{\Sigma} - \mathbf{S} & \cdots & \mathbf{\Sigma} - \mathbf{S} \\ \mathbf{\Sigma} - \mathbf{S} & \mathbf{\Sigma} & \cdots & \mathbf{\Sigma} - \mathbf{S} \\ \cdots & \cdots & \cdots & \cdots \\ \mathbf{\Sigma} - \mathbf{S} & \mathbf{\Sigma} - \mathbf{S} & \cdots & \mathbf{\Sigma} \end{pmatrix}_{p(m+1)\times p(m+1)}^{-1}.$$

This can be simplified as follows

$$\operatorname{tr}(\mathbf{G}_{\mathbf{S}}^{-1}) = m \operatorname{tr}(\mathbf{S}^{-1}) + \operatorname{tr}\left((m+1)\mathbf{\Sigma} - m\mathbf{S}\right)^{-1}.$$

Then using $(k\mathbf{A})^{-1} = k^{-1}\mathbf{A}^{-1}$ for matrix $\mathbf{A}$ and scalar k, we can scale the objective by m to get the MVR objective

$$m^2 \operatorname{tr}(\mathbf{S}^{-1}) + \operatorname{tr}\left(\frac{m+1}{m}\mathbf{\Sigma} - \mathbf{S}\right)^{-1}.$$

S1.10 Initializating coordinate descent algorithms

One plausible way to initialize the SDP/MVR/ME algorithms is to start at the equi-correlated solution $\mathbf{S}_{\text{eSDP}}$ due to its convenient closed-form solution (Dai and Barber 2016). However, note that the equi-correlated solution solves for the largest $\mathbf{S}$ matrix that satisfies $\frac{m+1}{m}\mathbf{\Sigma} - \mathbf{S} \succeq 0$, i.e. the smallest eigenvalue of $\mathbf{D} \equiv \frac{m+1}{m}\mathbf{\Sigma} - \mathbf{S}$ is numerically 0. This can cause the initial Cholesky factorization for $\mathbf{D}$ to fail. Thus, in `Knockoffs.jl`, we initialize the optimization problem with $\frac{1}{2}\mathbf{S}_{\text{eSDP}}$, which circumvents numerical issues but also serves as a reasonable starting point.

S1.11 Declaring convergence

Each of the SDP/MVR/ME group knockoff problems have an objective function g. In `Knockoffs.jl`, we declare convergence when either one of the conditions below is met

1. $\frac{|g^{new} - g|}{g} \leq \epsilon$
2. $\max_{i,j} |S_{ij}^{new} - S_{ij}| < 0.0001$

where the default $\epsilon = 0.0001$. The first condition checks if the objective improves, and exits if improvement is small. The second condition is checking whether the optimization variables $\mathbf{S}$ are changing sufficiently. This early exit criteria is motivated by the fact that model-X knockoffs control the FDR for any $\mathbf{S}$, and thus the estimation to $\mathbf{S}$ does not have to be very precise. In other words, if entries in $\mathbf{S}$ are not really changing, then optimization halts even if the objective can still be improved.

S1.12 Algorithm summary

Algorithm A1 summarizes the group knockoff optimization procedure in the ME case. For MVR and SDP, the overall structure remains the same, but the computation of δ needs to be modified according to the relevant equations derived in the sections above. Also, note that the algorithm summary does not check for backtracking, which is done in practice for better stability.

S2 PCA-based coordinate descent

Recall that, in PCA optimization, we perturb $\mathbf{S}$ via

$$\mathbf{S}^{new} = \mathbf{S} + \delta \mathbf{v}\mathbf{v}^t,$$

where $\mathbf{v}$ is a precomputed vector such that $\mathbf{v}^t\mathbf{v} = 1$ and the outer product $\mathbf{v}\mathbf{v}^t$ respects the block diagonal structure of $\mathbf{S}$. Here $\mathbf{v}$ can be viewed as a direction and δ a step size. Thus, we naturally would like to have a set of different proposed directions. One option is to precompute $\mathbf{v}_1, ..., \mathbf{v}_p$

Algorithm A1 Coordinate descent for ME group knockoffs (for MVR and SDP, only expression for δ changes but overall structure is the same)

Input: correlation matrix $\mathbf{\Sigma}_{p \times p}$, group membership vector, and number of knockoff copies to generate m
Initialize: $\mathbf{S} = \frac{1}{2}\mathbf{S}_{\text{eSDP}}$ with $\mathbf{S}_{\text{eSDP}}$ from (Dai and Barber 2016) and $\mathbf{D} = \frac{m+1}{m}\mathbf{\Sigma} - \mathbf{S}$
Compute: $(\mathbf{v}_1, ..., \mathbf{v}_p)$ based on eigendecomposition of $\mathbf{\Sigma}_{block}$ in eq 11 of main text
Compute: Cholesky factors $\mathbf{L}\mathbf{L}^t = \text{cholesky}(\mathbf{D})$ and $\mathbf{C}\mathbf{C}^t = \text{Cholesky}(\mathbf{S})$
while Not converged **do**
    ## PCA iterations
    for $\mathbf{v}_i \in (\mathbf{v}_1, ..., \mathbf{v}_p)$ **do**
        Compute constants $\mathbf{v}_i^t\mathbf{D}^{-1}\mathbf{v}_i, \mathbf{v}_i^t\mathbf{S}^{-1}\mathbf{v}_i$ from $\mathbf{L}, \mathbf{C}$ via method derived in section S1.8
        Compute δ in Eq (S.10)
        Rank-1 update: $\mathbf{S}_{new} = \mathbf{S} + \delta\mathbf{v}_i\mathbf{v}_i^t$
        Update Cholesky factors $\mathbf{L}_{new}\mathbf{L}_{new}^t = \frac{m+1}{m}\mathbf{\Sigma} - \mathbf{S}_{new}$ and $\mathbf{C}_{new}\mathbf{C}_{new}^t = \mathbf{S}_{new}$
    end for
    ## Full optimization
    for $\gamma = \{1, ..., g\}$ **do**
        for (i, j) in group γ **do**
            if $i = j$ **then**
                Compute constants a_{jj}, b_{jj} from $\mathbf{L}, \mathbf{C}$ via method in section S1.8
                Compute δ by Eq (S.2)
                Clamp δ to be within feasible region derived in Eq (S.5)
                Rank-1 update: $S_{ii}^{new} = S_{ii} + \delta$
            else
                Compute constants $a_{ii}, a_{jj}, a_{ij}, b_{ii}, b_{jj}, b_{ij}$ from $\mathbf{L}, \mathbf{C}$ via method in section S1.8
                Compute δ by solving 1-D optimization problem in (S.1)
                Clamp δ to be within feasible region derived in Eq (S.7)
                Rank-2 update: $S_{ij}^{new} = S_{ji}^{new} = S_{ij} + \delta$
            end if
            Update Cholesky factors $\mathbf{L}_{new}\mathbf{L}_{new}^t = \frac{m+1}{m}\mathbf{\Sigma} - \mathbf{S}_{new}$ and $\mathbf{C}_{new}\mathbf{C}_{new}^t = \mathbf{S}_{new}$
        end for
    end for
end while
Output: Group-block-diagonal matrix $\mathbf{S}$ satisfying $\frac{m+1}{m}\mathbf{\Sigma} - \mathbf{S} \succeq 0$ and $\mathbf{S} \succeq 0$.

where $\mathbf{v}_i$ is the ith eigenvector (hence the name "PCA-based") of the block diagonalized covariance matrix

$$\mathbf{\Sigma}_{blocked} = \begin{bmatrix} \mathbf{\Sigma}_1 & & \\ & \ddots & \\ & & \mathbf{\Sigma}_g \end{bmatrix}_{p\times p}. \tag{S.9}$$

Eigendecomposition of $\mathbf{\Sigma}_{blocked}$ is efficient due to its block structure, since we can just compute the eigenvectors for each block and pad them with zeros. We can obviously include more directions as long as the outer product $\mathbf{v}\mathbf{v}^t$ respects the block diagonal structure of $\mathbf{S}$. For example, adding p basis vectors (which allows updating just the diagonal entries) dramatically speeds up convergence.

S2.1 PCA-based Coordinate descent for ME

We will optimize

$$\max_{\mathbf{S}} \log\det\left(\frac{m+1}{m}\mathbf{\Sigma} - \mathbf{S}\right) + m\log\det(\mathbf{S}) \quad \text{subject to } \begin{cases} \frac{m+1}{m}\mathbf{\Sigma} - \mathbf{S} \succeq 0 \\ \mathbf{S} \succeq 0 \end{cases}.$$

Consider updating $\mathbf{S}^{new} = \mathbf{S} + \delta\mathbf{v}\mathbf{v}^t$. If $\mathbf{D} = \frac{m+1}{m}\mathbf{\Sigma} - \mathbf{S}$, then the objective becomes

$$\begin{aligned} g(\mathbf{S} + \delta\mathbf{v}\mathbf{v}^t) &= \log\det(\mathbf{D} - \delta\mathbf{v}\mathbf{v}^t) + m\log\det(\mathbf{S} + \delta\mathbf{v}\mathbf{v}^t) \\ &= \log\left[(1 - \delta\mathbf{v}^t\mathbf{D}^{-1}\mathbf{v})\det(\mathbf{D})\right] + m\log\left[(1 + \delta\mathbf{v}^t\mathbf{S}^{-1}\mathbf{v})\det(\mathbf{S})\right] \\ &\propto \log(1 - \delta\mathbf{v}^t\mathbf{D}^{-1}\mathbf{v}) + m\log(1 + \delta\mathbf{v}^t\mathbf{S}^{-1}\mathbf{v}), \end{aligned}$$

where the second equality follows from the matrix determinant lemma. The first-order optimality condition is

$$\frac{d}{d\delta}g = \frac{-\mathbf{v}^t\mathbf{D}^{-1}\mathbf{v}}{1 - \delta\mathbf{v}^t\mathbf{D}^{-1}\mathbf{v}} + \frac{m\mathbf{v}^t\mathbf{S}^{-1}\mathbf{v}}{1 + \delta\mathbf{v}^t\mathbf{S}^{-1}\mathbf{v}} = 0.$$

Thus,

$$\delta = \frac{m\mathbf{v}^t\mathbf{S}^{-1}\mathbf{v} - \mathbf{v}^t\mathbf{D}^{-1}\mathbf{v}}{(m+1)\mathbf{v}^t\mathbf{S}^{-1}\mathbf{v}\mathbf{v}^t\mathbf{D}^{-1}\mathbf{v}}. \tag{S.10}$$

Simple algebraic manipulation reveals that δ satisfies

$$\frac{-1}{\mathbf{v}^t\mathbf{S}^{-1}\mathbf{v}} \le \delta \le \frac{1}{\mathbf{v}^t\mathbf{D}^{-1}\mathbf{v}}.$$

As derived in section S1.6, any δ within this range will satisfy the PSD constraints. Constants $\mathbf{v}^t\mathbf{D}^{-1}\mathbf{v}$ and $\mathbf{v}^t\mathbf{S}^{-1}\mathbf{v}$ can be extracted efficiently with the strategy outlined in section S1.8 as long as we have Cholesky factors of $\mathbf{S}$ and $\mathbf{D}$. Finally, we efficiently update the objective

$$g(\mathbf{S}^{new}) = \log(1 - \delta\mathbf{v}^t\mathbf{D}^{-1}\mathbf{v}) + m\log(1 + \delta\mathbf{v}^t\mathbf{S}^{-1}\mathbf{v}) + g(\mathbf{S}).$$

S2.2 PCA-based coordinate descent for MVR.

As shown above, the MVR objective can be written as

$$\min_{\mathbf{S}} m^2 \operatorname{tr}(\mathbf{S})^{-1} + \operatorname{tr}\left(\frac{m+1}{m}\boldsymbol{\Sigma} - \mathbf{S}\right)^{-1} \quad \text{subject to } \begin{cases} \frac{M+1}{M}\boldsymbol{\Sigma} - \mathbf{S} \succeq 0 \\ \mathbf{S} \succeq 0 \end{cases}.$$

Consider updating $\mathbf{S}^{new} = \mathbf{S} + \delta\mathbf{v}\mathbf{v}^t$. If $\mathbf{D} = \frac{m+1}{m}\boldsymbol{\Sigma} - \mathbf{S}$, then the objective becomes

$$\begin{aligned} g(\mathbf{S} + \delta\mathbf{v}\mathbf{v}^t) &= m^2 \operatorname{tr}\left((\mathbf{S} + \delta\mathbf{v}\mathbf{v}^t)^{-1}\right) + \operatorname{tr}\left((\mathbf{D} - \delta\mathbf{v}\mathbf{v}^t)^{-1}\right) \\ &= m^2 \operatorname{tr}\left(\mathbf{S}^{-1} - \delta\frac{\mathbf{S}^{-1}\mathbf{v}\mathbf{v}^t\mathbf{S}^{-1}}{1+\delta\mathbf{v}^t\mathbf{S}^{-1}\mathbf{v}}\right) + \operatorname{tr}\left(\mathbf{D}^{-1} + \delta\frac{\mathbf{D}^{-1}\mathbf{v}\mathbf{v}^t\mathbf{D}^{-1}}{1-\delta\mathbf{v}^t\mathbf{D}^{-1}\mathbf{v}}\right) \quad \text{(Sherman-Morrison)} \\ &= \frac{-m^2\delta\mathbf{v}^t\mathbf{S}^{-2}\mathbf{v}}{1+\delta\mathbf{v}^t\mathbf{S}^{-1}\mathbf{v}} + \frac{\delta\mathbf{v}^t\mathbf{D}^{-2}\mathbf{v}}{1-\delta\mathbf{v}^t\mathbf{D}^{-1}\mathbf{v}} + g(\mathbf{S}) \quad \text{(Cyclic property of trace).} \end{aligned}$$

The first-order optimality states

$$\begin{aligned} 0 &= \delta^2(-m^2(\mathbf{v}^t\mathbf{D}^{-1}\mathbf{v})^2\mathbf{v}^t\mathbf{S}^{-2}\mathbf{v} + (\mathbf{v}^t\mathbf{S}^{-1}\mathbf{v})^2\mathbf{v}^t\mathbf{D}^{-2}\mathbf{v}) + \\ &\quad \delta(2m^2\mathbf{v}^t\mathbf{D}^{-1}\mathbf{v}\mathbf{v}^t\mathbf{S}^{-2}\mathbf{v} + 2\mathbf{v}^t\mathbf{S}^{-1}\mathbf{v}\mathbf{v}\mathbf{D}^{-2}\mathbf{v}) - m^2\mathbf{v}^t\mathbf{S}^{-2}\mathbf{v} + \mathbf{v}\mathbf{D}^{-2}\mathbf{v}. \end{aligned}$$

Thus,

$$\delta = \frac{-b \pm \sqrt{b^2-4ac}}{2a} \text{ where } \begin{cases} a &= -m^2(\mathbf{v}^t\mathbf{D}^{-1}\mathbf{v})^2\mathbf{v}^t\mathbf{S}^{-2}\mathbf{v} + (\mathbf{v}^t\mathbf{S}^{-1}\mathbf{v})^2\mathbf{v}^t\mathbf{D}^{-2}\mathbf{v} \\ b &= 2m^2\mathbf{v}^t\mathbf{D}^{-1}\mathbf{v}\mathbf{v}^t\mathbf{S}^{-2}\mathbf{v} + 2\mathbf{v}^t\mathbf{S}^{-1}\mathbf{v}\mathbf{v}\mathbf{D}^{-2}\mathbf{v} \\ c &= -m^2\mathbf{v}^t\mathbf{S}^{-2}\mathbf{v} + \mathbf{v}\mathbf{D}^{-2}\mathbf{v} \end{cases}. \quad \text{(S.11)}$$

A unique solution for δ exists given the boundary condition

$$\frac{-1}{\mathbf{v}^t\mathbf{S}^{-1}\mathbf{v}} \leq \delta \leq \frac{1}{\mathbf{v}^t\mathbf{D}^{-1}\mathbf{v}}.$$

S2.3 PCA-based coordinate descent for SDP

We will optimize

$$\min_{\mathbf{S}} \sum_{\gamma=1}^{g} \frac{1}{|\mathcal{A}_\gamma|^2} \sum_{i,j\in\mathcal{A}_\gamma} |\Sigma_{ij} - S_{ij}| \quad \text{subject to } \begin{cases} \frac{m+1}{m}\boldsymbol{\Sigma} - \mathbf{S} \succeq 0 \\ \mathbf{S} \succeq 0 \end{cases}.$$

Consider updating $\mathbf{S}^{new} = \mathbf{S} + \delta\mathbf{v}\mathbf{v}^t$, the objective becomes

$$g(\mathbf{S} + \delta\mathbf{v}\mathbf{v}^t) = \sum_{\gamma=1}^{g} \frac{1}{|\mathcal{A}_\gamma|^2} \sum_{i,j\in\mathcal{A}_\gamma} |\Sigma_{ij} - S_{ij} - \delta v_i v_j|. \quad \text{(S.12)}$$

Although a closed form solution may exist for this problem, our software solves it numerically using Brent's method implemented in `Optim.jl`. This is a very fast operation because $\mathbf{v}\mathbf{v}^t$ respects the block diagonal structure of $\mathbf{S}$, and thus only a single block in $\mathbf{S}$ depends on δ.

S3 Proof of Theorem 1

To prove Theorem 1 in the main paper, we need to show that when knockoffs $\widetilde{X}$ are generated under Algorithm 2, the distribution of $(X, \tilde{X})$ satisfies both the conditional indepedendence and the group exchangeability.

According to Algorithm 2, $\tilde{X}$ is generated using only information of X without looking at the response Y. Thus, the conditional independence stands.

To prove group exchangeability of the distribution of $(X, \tilde{X})$, we rearrange elements of X such that $X = (X_1^\star, \ldots, X_g^\star, X_1^\dagger, \ldots, X_g^\dagger)$. According to step 2 of Algorithm 2, it is clear that

$$(X_1^\star, \ldots, X_g^\star, \tilde{X}_1^\star, \ldots, \tilde{X}_g^\star)_{\text{swap}(\mathcal{C})} \overset{d}{=} (X_1^\star, \ldots, X_g^\star, \tilde{X}_1^\star, \ldots, \tilde{X}_g^\star) \quad \forall\ \mathcal{C}: \ \ \mathcal{C} = \cup_{\gamma \in \mathcal{S}} \mathcal{A}_\gamma^\star.$$

In other words, for any value $x_1^\star, \ldots, x_g^\star, \tilde{x}_1^\star, \ldots, \tilde{x}_g^\star$, it is equally possible for

$$(X^\star, \tilde{X}^\star) = (X_1^\star, \ldots, X_g^\star, \tilde{X}_1^\star, \ldots, \tilde{X}_g^\star) \quad \text{and} \quad (X^\star, \tilde{X}^\star)_{\text{swap}(\mathcal{C})} = (X_1^\star, \ldots, X_g^\star, \tilde{X}_1^\star, \ldots, \tilde{X}_g^\star)_{\text{swap}(\mathcal{C})}$$

to take values $(x^\star, \tilde{x}^\star) = (x_1^\star, \ldots, x_g^\star, \tilde{x}_1^\star, \ldots, \tilde{x}_g^\star)$.

By Definition 1 and steps 3-4 of Algorithm 2, the probability that $(X^\star, X^\dagger, \tilde{X}^\star, \tilde{X}^\dagger)$ takes values $(x^\star, x^\dagger, \tilde{x}^\star, \tilde{x}^\dagger)$ is equal to

$$\Pr\left\{(X^\star, \tilde{X}^\star) = (x^\star, \tilde{x}^\star)\right\} \times \prod_{\gamma=1}^{g} \left\{F_\gamma(\widetilde{x}_\gamma^\dagger | \widetilde{x}_\gamma^\star) F_\gamma(x_\gamma^\dagger | x_\gamma^\star)\right\}. \tag{S.13}$$

Because

$$\Pr\left\{(X^\star, \tilde{X}^\star)_{\text{swap}(\mathcal{C})} = (x^\star, \tilde{x}^\star)\right\} \times \prod_{\gamma=1}^{g} \left\{F_\gamma(\widetilde{x}_\gamma^\dagger | \widetilde{x}_\gamma^\star) F_\gamma(x_\gamma^\dagger | x_\gamma^\star)\right\} = (\text{S.13}),$$

we have

$$(X^\star, X^\dagger, \tilde{X}^\star, \tilde{X}^\dagger)_{\text{swap}(\mathcal{C})} \overset{d}{=} (X^\star, X^\dagger, \tilde{X}^\star, \tilde{X}^\dagger)$$

and thus the group exchangeability stands.

S4 Proof of Theorem 2

To prove Theorem 2 in the main text, we need to

- first derive sufficient and necessary conditions of the minimizers of $L_{\text{ME}}(\mathbf{S}) = \log\det(\mathbf{G}_\mathbf{S}^{-1})$ in the case that X has the group key conditional independence property with respect to $\{\mathcal{A}_\gamma\}_{\gamma=1}^g$;
- and then show the variance-covariance matrix of $(X, \tilde{X})$ generated according to Algorithm 2 and minimizing $L_{\text{ME}}^\star(S^\star)$ satisfies all sufficient and necessary conditions.

S4.1 Sufficient and Necessary Conditions of the minimizer of $L_{\mathbf{ME}}(\mathbf{S})$

For simplicity, we rearrange elements of X such that $X = (X_1^\star, \ldots, X_g^\star, X_1^\dagger, \ldots, X_g^\dagger)$ whose variance-covariance matrix is

$$\mathbf{\Sigma} = \left(\begin{array}{c:c} \mathbf{\Sigma}^\star & \mathbf{\Sigma}^{\star\dagger} \\ \hdashline (\mathbf{\Sigma}^{\star\dagger})^T & \mathbf{\Sigma}^\dagger \end{array}\right) = \left(\begin{array}{cccc:cccc} \mathbf{\Sigma}_{11}^{\star\star} & \mathbf{\Sigma}_{12}^{\star\star} & \cdots & \mathbf{\Sigma}_{1g}^{\star\star} & \mathbf{\Sigma}_{11}^{\star\dagger} & \mathbf{\Sigma}_{12}^{\star\dagger} & \cdots & \mathbf{\Sigma}_{1g}^{\star\dagger} \\ \mathbf{\Sigma}_{21}^{\star\star} & \mathbf{\Sigma}_{22}^{\star\star} & \cdots & \mathbf{\Sigma}_{2g}^{\star\star} & \mathbf{\Sigma}_{21}^{\star\dagger} & \mathbf{\Sigma}_{22}^{\star\dagger} & \cdots & \mathbf{\Sigma}_{2g}^{\star\dagger} \\ \vdots & \vdots & \ddots & \vdots & \vdots & \vdots & \ddots & \vdots \\ \mathbf{\Sigma}_{g1}^{\star\star} & \mathbf{\Sigma}_{g2}^{\star\star} & \cdots & \mathbf{\Sigma}_{gg}^{\star\star} & \mathbf{\Sigma}_{g1}^{\star\dagger} & \mathbf{\Sigma}_{g2}^{\star\dagger} & \cdots & \mathbf{\Sigma}_{gg}^{\star\dagger} \\ \hdashline \mathbf{\Sigma}_{11}^{\dagger\star} & \mathbf{\Sigma}_{12}^{\dagger\star} & \cdots & \mathbf{\Sigma}_{1g}^{\dagger\star} & \mathbf{\Sigma}_{11}^{\dagger\dagger} & \mathbf{\Sigma}_{12}^{\dagger\dagger} & \cdots & \mathbf{\Sigma}_{1g}^{\dagger\dagger} \\ \mathbf{\Sigma}_{21}^{\dagger\star} & \mathbf{\Sigma}_{22}^{\dagger\star} & \cdots & \mathbf{\Sigma}_{2g}^{\dagger\star} & \mathbf{\Sigma}_{21}^{\dagger\dagger} & \mathbf{\Sigma}_{22}^{\dagger\dagger} & \cdots & \mathbf{\Sigma}_{2g}^{\dagger\dagger} \\ \vdots & \vdots & \ddots & \vdots & \vdots & \vdots & \ddots & \vdots \\ \mathbf{\Sigma}_{g1}^{\dagger\star} & \mathbf{\Sigma}_{g2}^{\dagger\star} & \cdots & \mathbf{\Sigma}_{gg}^{\dagger\star} & \mathbf{\Sigma}_{g1}^{\dagger\dagger} & \mathbf{\Sigma}_{g2}^{\dagger\dagger} & \cdots & \mathbf{\Sigma}_{gg}^{\dagger\dagger} \end{array}\right).$$

By equation (5) of the main text, we have correspondingly

$$\mathbf{G}_\mathbf{S} = \left(\begin{array}{c:c} \mathbf{G}_{\mathbf{S}^\star}^\star & \mathbf{G}_\mathbf{S}^{\star\dagger} \\ \hdashline (\mathbf{G}_\mathbf{S}^{\star\dagger})^T & \mathbf{G}_\mathbf{S}^\dagger \end{array}\right)$$

where

$$\mathbf{G}_{\mathbf{S}^\star}^\star = \begin{pmatrix} \mathbf{\Sigma}^\star & \mathbf{\Sigma}^\star - \mathbf{S}^\star \\ \mathbf{\Sigma}^\star - \mathbf{S}^\star & \mathbf{\Sigma}^\star \end{pmatrix}, \quad \mathbf{S}^\star = \mathrm{diag}(\mathbf{S}_1^\star, \mathbf{S}_2^\star, \ldots, \mathbf{S}_g^\star),$$
$$\mathbf{G}_{\mathbf{S}^\star}^{\star\dagger} = \begin{pmatrix} \mathbf{\Sigma}^{\star\dagger} & \mathbf{\Sigma}^{\star\dagger} - \mathbf{S}^{\star\dagger} \\ \mathbf{\Sigma}^{\star\dagger} - \mathbf{S}^{\star\dagger} & \mathbf{\Sigma}^{\star\dagger} \end{pmatrix}, \quad \mathbf{S}^{\star\dagger} = \mathrm{diag}(\mathbf{S}_1^{\star\dagger}, \mathbf{S}_2^{\star\dagger}, \ldots, \mathbf{S}_g^{\star\dagger}),$$
$$\mathbf{G}_\mathbf{S}^\dagger = \begin{pmatrix} \mathbf{\Sigma}^\dagger & \mathbf{\Sigma}^\dagger - \mathbf{S}^\dagger \\ \mathbf{\Sigma}^\dagger - \mathbf{S}^\dagger & \mathbf{\Sigma}^\dagger \end{pmatrix}, \quad \mathbf{S}^\dagger = \mathrm{diag}(\mathbf{S}_1^\dagger, \mathbf{S}_2^\dagger, \ldots, \mathbf{S}_g^\dagger).$$

Consider a transformation matrix

$$\mathbf{Q} = \left(\begin{array}{cc:cc} \mathbf{I} & \mathbf{0} & \mathbf{Q}^{\star\dagger} & \mathbf{0} \\ \mathbf{0} & \mathbf{I} & \mathbf{0} & \mathbf{Q}^{\star\dagger} \\ \hdashline \mathbf{0} & \mathbf{0} & \mathbf{I} & \mathbf{0} \\ \mathbf{0} & \mathbf{0} & \mathbf{0} & \mathbf{I} \end{array}\right), \quad \text{where } \mathbf{Q}^{\star\dagger} = -\mathrm{diag}((\mathbf{\Sigma}_{11}^{\star\star})^{-1}\mathbf{\Sigma}_{11}^{\star\dagger}, (\mathbf{\Sigma}_{22}^{\star\star})^{-1}\mathbf{\Sigma}_{22}^{\star\dagger}, \ldots, (\mathbf{\Sigma}_{gg}^{\star\star})^{-1}\mathbf{\Sigma}_{gg}^{\star\dagger}).$$

Because X has group key conditional independence property with respect to $\{\mathcal{A}_\gamma\}_{\gamma=1}^g$, we have for any $\gamma_1 \neq \gamma_2$,

$$\begin{cases} \mathbf{\Sigma}_{\gamma_1\gamma_2}^{\star\dagger} = \mathbf{\Sigma}_{\gamma_1\gamma_2}^{\star\star}(\mathbf{\Sigma}_{\gamma_2\gamma_2}^{\star\star})^{-1}\mathbf{\Sigma}_{\gamma_2\gamma_2}^{\star\dagger} \\ \mathbf{\Sigma}_{\gamma_1\gamma_2}^{\dagger\dagger} = \mathbf{\Sigma}_{\gamma_1\gamma_2}^{\dagger\star}(\mathbf{\Sigma}_{\gamma_2\gamma_2}^{\star\star})^{-1}\mathbf{\Sigma}_{\gamma_2\gamma_2}^{\star\dagger}. \end{cases}$$

Therefore,

$$\begin{cases} \mathbf{\Sigma}^\star\mathbf{Q}^{\star\dagger} + \mathbf{\Sigma}^{\star\dagger} = \mathbf{0} \\ (\mathbf{\Sigma}^{\star\dagger})^T\mathbf{Q}^{\star\dagger} + \mathbf{\Sigma}^\dagger = \mathrm{diag}(\mathbf{\Sigma}_{11|1}^{\dagger\dagger|\star}, \mathbf{\Sigma}_{22|2}^{\dagger\dagger|\star}, \ldots, \mathbf{\Sigma}_{gg|g}^{\dagger\dagger|\star}), \end{cases} \tag{S.14}$$

leading to a transformed variance-covariance matrix

$$\check{\mathbf{G}}_\mathbf{S} = \mathbf{Q}^T\mathbf{G}_\mathbf{S}\mathbf{Q} = \left(\begin{array}{c:c} \mathbf{G}_{\mathbf{S}^\star}^\star & \check{\mathbf{G}}_\mathbf{S}^{\star\dagger} \\ \hdashline (\check{\mathbf{G}}_\mathbf{S}^{\star\dagger})^T & \check{\mathbf{G}}_\mathbf{S}^\dagger \end{array}\right)$$

where

$$
\check{\mathbf{G}}^{\star\dagger}_{\mathbf{S}^\star} = \begin{pmatrix} \mathbf{0} & -\check{\mathbf{S}}^{\star\dagger} \\ -\check{\mathbf{S}}^{\star\dagger} & \mathbf{0} \end{pmatrix}, \quad \check{\mathbf{S}}^{\star\dagger} = \mathbf{S}^\star\mathbf{Q}^{\star\dagger} + \mathbf{S}^{\star\dagger},
$$

$$
\check{\mathbf{G}}^{\dagger}_{\mathbf{S}} = \begin{pmatrix} \mathrm{diag}(\mathbf{\Sigma}^{\dagger\dagger|\star}_{11|1}, \mathbf{\Sigma}^{\dagger\dagger|\star}_{22|2}, \ldots, \mathbf{\Sigma}^{\dagger\dagger|\star}_{gg|g}) & \mathrm{diag}(\mathbf{\Sigma}^{\dagger\dagger|\star}_{11|1}, \mathbf{\Sigma}^{\dagger\dagger|\star}_{22|2}, \ldots, \mathbf{\Sigma}^{\dagger\dagger|\star}_{gg|g}) - \check{\mathbf{S}}^{\dagger} \\ \mathrm{diag}(\mathbf{\Sigma}^{\dagger\dagger|\star}_{11|1}, \mathbf{\Sigma}^{\dagger\dagger|\star}_{22|2}, \ldots, \mathbf{\Sigma}^{\dagger\dagger|\star}_{gg|g}) - \check{\mathbf{S}}^{\dagger} & \mathrm{diag}(\mathbf{\Sigma}^{\dagger\dagger|\star}_{11|1}, \mathbf{\Sigma}^{\dagger\dagger|\star}_{22|2}, \ldots, \mathbf{\Sigma}^{\dagger\dagger|\star}_{gg|g}) \end{pmatrix},
$$

$$
\check{\mathbf{S}}^{\dagger} = (\mathbf{Q}^{\star\dagger})^T\mathbf{S}^\star\mathbf{Q}^{\star\dagger} + (\mathbf{Q}^{\star\dagger})^T\mathbf{S}^{\star\dagger} + (\mathbf{S}^{\star\dagger})^T\mathbf{Q}^{\star\dagger} + \mathbf{S}^{\dagger},
$$

As a result, we observe that

1. Minimizing $L_{\mathrm{ME}}(\mathbf{S}) = \log\det(\mathbf{G}_{\mathbf{S}}^{-1})$ is equivalent to maximizing $\det(\mathbf{G}_{\mathbf{S}})$.
2. Since $\det(\mathbf{Q}) = 1$, we have $\det(\check{\mathbf{G}}_{\mathbf{S}}) = \det(\mathbf{G}_{\mathbf{S}})$ and thus minimizing L_{ME} is equivalent to maximizing $\det(\check{\mathbf{G}}_{\mathbf{S}})$.
3. There exists a one-to-one correspondence between $(\mathbf{S}^\star, \mathbf{S}^{\star\dagger}, \mathbf{S}^{\dagger})$ and $(\mathbf{S}^\star, \check{\mathbf{S}}^{\star\dagger}, \check{\mathbf{S}}^{\dagger})$.
4. For any $(\mathbf{S}^\star, \check{\mathbf{S}}^{\dagger})$, $\det(\check{\mathbf{G}}_{\mathbf{S}})$ is maximized if and only if $\check{\mathbf{S}}^{\star\dagger} = \mathbf{0}$ or equivalently,
$$
\mathbf{S}^{\star\dagger} = -\mathbf{S}^\star\mathbf{Q}^{\star\dagger}. \tag{S.15}
$$
5. Given (S.15), for any $\mathbf{S}^\star$, $\det(\check{\mathbf{G}}_{\mathbf{S}})$ is maximized if and only if $\check{\mathbf{S}}^{\dagger} = \mathrm{diag}(\mathbf{\Sigma}^{\dagger\dagger|\star}_{11|1}, \mathbf{\Sigma}^{\dagger\dagger|\star}_{22|2}, \ldots, \mathbf{\Sigma}^{\dagger\dagger|\star}_{gg|g})$ or equivalently,
$$
\begin{aligned}
\mathbf{S}^{\dagger} &= \mathrm{diag}(\mathbf{\Sigma}^{\dagger\dagger|\star}_{11|1}, \mathbf{\Sigma}^{\dagger\dagger|\star}_{22|2}, \ldots, \mathbf{\Sigma}^{\dagger\dagger|\star}_{gg|g}) - (\mathbf{Q}^{\star\dagger})^T\mathbf{S}^\star - \mathbf{S}^\star\mathbf{Q}^{\star\dagger} - (\mathbf{Q}^{\star\dagger})^T\mathbf{S}^\star\mathbf{Q}^{\star\dagger} \\
&= \mathrm{diag}(\mathbf{\Sigma}^{\dagger\dagger|\star}_{11|1}, \mathbf{\Sigma}^{\dagger\dagger|\star}_{22|2}, \ldots, \mathbf{\Sigma}^{\dagger\dagger|\star}_{gg|g}) + (\mathbf{Q}^{\star\dagger})^T\mathbf{S}^\star\mathbf{Q}^{\star\dagger} \qquad \text{(S.16)}
\end{aligned}
$$
for $\gamma = 1, \ldots, g$.
6. Given (S.15)-(S.16), $\det(\check{\mathbf{G}}_{\mathbf{S}})$ is maximized if and only if $\det(\mathbf{G}^\star_{\mathbf{S}^\star})$ is maximized or equivalently,
$$
L^\star_{\mathrm{ME}}(S^\star) \text{ is minimized.} \tag{S.17}
$$

In summary, (S.15)-(S.17) are sufficient and necessary conditions of the minimizer of $L_{\mathrm{ME}}(\mathbf{S}) = \log\det(\mathbf{G}_{\mathbf{S}}^{-1})$ if X has the group key conditional independence property with respect to $\{\mathcal{A}_\gamma\}_{\gamma=1}^g$.

In the following, we need to show that the variance-covariance matrix of $(X, \tilde{X})$ generated according to Algorithm 2 and minimizing $L^\star_{\mathrm{ME}}(S^\star)$ satisfies (S.15)-(S.17).

- It is trivial that (S.17) is satisfied.
- Because $(X, \tilde{X})$ generated according to Algorithm 2 has the group key conditional independence property with respect to $\{\mathcal{A}_\gamma, \tilde{\mathcal{A}}_\gamma\}_{\gamma=1}^g$, we have by (S.14),

$$
\begin{aligned}
\mathrm{Cov}\left\{(X_\star, \tilde{X}_\star), (X_\dagger, \tilde{X}_\dagger)\right\} &= \mathrm{Cov}\left\{(X_\star, \tilde{X}_\star), (X_\star, \tilde{X}_\star)\right\} \begin{pmatrix} -\mathbf{Q}^{\star\dagger} & \mathbf{0} \\ \mathbf{0} & -\mathbf{Q}^{\star\dagger} \end{pmatrix} \\
&= \begin{pmatrix} \mathbf{\Sigma}^\star & \mathbf{\Sigma}^\star - \mathbf{S}^\star \\ \mathbf{\Sigma}^\star - \mathbf{S}^\star & \mathbf{\Sigma}^\star \end{pmatrix} \begin{pmatrix} -\mathbf{Q}^{\star\dagger} & \mathbf{0} \\ \mathbf{0} & -\mathbf{Q}^{\star\dagger} \end{pmatrix} \\
&= \begin{pmatrix} \mathbf{\Sigma}^{\star\dagger} & \mathbf{\Sigma}^{\star\dagger} - (-\mathbf{S}^{\star\dagger}\mathbf{Q}^{\star\dagger}) \\ \mathbf{\Sigma}^{\star\dagger} - (-\mathbf{S}^{\star\dagger}\mathbf{Q}^{\star\dagger}) & \mathbf{\Sigma}^{\star\dagger} \end{pmatrix},
\end{aligned}
$$

and thus (S.15) is satisfied. In addition, by (S.14),

$$
\begin{aligned}
&\operatorname{Cov}\left\{(X_\dagger, \tilde{X}_\dagger), (X_\dagger, \tilde{X}_\dagger)\right\} \\
=&\begin{pmatrix} -(\mathbf{Q}^{\star\dagger})^T & \mathbf{0} \\ \mathbf{0} & -(\mathbf{Q}^{\star\dagger})^T \end{pmatrix} \operatorname{Cov}\left\{(X_\star, \tilde{X}_\star), (X_\dagger, \tilde{X}_\dagger)\right\} \\
&+ \begin{pmatrix} \operatorname{diag}(\mathbf{\Sigma}_{11|1}^{\dagger\dagger|\star}, \mathbf{\Sigma}_{22|2}^{\dagger\dagger|\star}, \ldots, \mathbf{\Sigma}_{gg|g}^{\dagger\dagger|\star}) & \mathbf{0} \\ \mathbf{0} & \operatorname{diag}(\mathbf{\Sigma}_{11|1}^{\dagger\dagger|\star}, \mathbf{\Sigma}_{22|2}^{\dagger\dagger|\star}, \ldots, \mathbf{\Sigma}_{gg|g}^{\dagger\dagger|\star}) \end{pmatrix} \\
=&\begin{pmatrix} -(\mathbf{Q}^{\star\dagger})^T & \mathbf{0} \\ \mathbf{0} & -(\mathbf{Q}^{\star\dagger})^T \end{pmatrix} \begin{pmatrix} \mathbf{\Sigma}^{\star\dagger} & \mathbf{\Sigma}^{\star\dagger} - (-\mathbf{S}^{\star\dagger}\mathbf{Q}^{\star\dagger}) \\ \mathbf{\Sigma}^{\star\dagger} - (-\mathbf{S}^{\star\dagger}\mathbf{Q}^{\star\dagger}) & \mathbf{\Sigma}^{\star\dagger} \end{pmatrix} \\
&+ \begin{pmatrix} \operatorname{diag}(\mathbf{\Sigma}_{11|1}^{\dagger\dagger|\star}, \mathbf{\Sigma}_{22|2}^{\dagger\dagger|\star}, \ldots, \mathbf{\Sigma}_{gg|g}^{\dagger\dagger|\star}) & \mathbf{0} \\ \mathbf{0} & \operatorname{diag}(\mathbf{\Sigma}_{11|1}^{\dagger\dagger|\star}, \mathbf{\Sigma}_{22|2}^{\dagger\dagger|\star}, \ldots, \mathbf{\Sigma}_{gg|g}^{\dagger\dagger|\star}) \end{pmatrix} \\
=&\begin{pmatrix} \mathbf{\Sigma}^{\dagger} & \mathbf{\Sigma}^{\dagger} - ((\mathbf{Q}^{\star\dagger})^T\mathbf{S}^{\star}\mathbf{Q}^{\star\dagger} + \operatorname{diag}(\mathbf{\Sigma}_{11|1}^{\dagger\dagger|\star}, \mathbf{\Sigma}_{22|2}^{\dagger\dagger|\star}, \ldots, \mathbf{\Sigma}_{gg|g}^{\dagger\dagger|\star})) \\ \mathbf{\Sigma}^{\dagger} - ((\mathbf{Q}^{\star\dagger})^T\mathbf{S}^{\star}\mathbf{Q}^{\star\dagger} + \operatorname{diag}(\mathbf{\Sigma}_{11|1}^{\dagger\dagger|\star}, \mathbf{\Sigma}_{22|2}^{\dagger\dagger|\star}, \ldots, \mathbf{\Sigma}_{gg|g}^{\dagger\dagger|\star})) & \mathbf{\Sigma}^{\dagger} \end{pmatrix}
\end{aligned}
$$

and thus (S.16) is satisfied.

S5 Heuristic strategy to identify groups and group-key variables to approximately achieve conditional independence

There are many ways for defining groups and selecting their representatives. In this section, we present 2 plausible strategies for constructing groups using interpolative decomposition or hierarchical clustering. Then we discuss the intuition of Algorithm A2 for selecting group-key representatives from each group.

S5.1 Defining groups by Hierarchical clustering

The standard approach for defining groups is to use hierarchical clustering, e.g. as featured in (Sesia et al. 2021). In this approach, we use the absolute value of the empirical correlation matrix as the distance matrix

$$d_{ij} = 1 - |corr(X_i, X_j)| = 1 - |\Sigma_{ij}|,$$

where $d_{ij} \in [0, 1]$ represents the distance between features i and j. Thus, $d_{ii} = 0$, and more correlated variables are considered "closer". Groups are defined by applying (single, average, or complete linkage) hierarchical clustering to the resulting distance matrix, with some correlation cutoff. When contiguous groups are desired, we can use adjacency-constrained hierarchical clustering rather than standard hierarchical clustering.

S5.2 Defining groups by Interpolative Decomposition (ID)

Given design matrix $\mathbf{X} \in \mathbb{R}^{n\times p}$ with highly correlated columns, the ID approach selects a subset $J \subset \{1, ..., p\}$ of k columns from $\mathbf{X}$ such that

$$\underset{n\times(p-k)}{\mathbf{X}_{J^c}} \approx \underset{n\times k}{\mathbf{X}_J} \times \underset{k\times(p-k)}{\mathbf{T}}.$$

The selected columns in J are sometimes called the *skeleton* columns, the non selected columns $J^c = \{1, ..., p\} \backslash J$ are called the *redundant* columns, and $\mathbf{T}$ is the interpolation matrix. Thus, ID is trying to represent the redundant columns via the skeleton columns.

To define groups, we will let the J skeleton columns be "group centers". For a variable i that is not a cluster center, we put it in the same group as cluster center j that is most correlated with i, i.e. $\max_j |corr(x_i, x_j)|$. If we would like to enforce contiguous groups, we simply assign i to its left or right center, whichever is more correlated with i, without breaking the adjacency constraint.

S5.2.1 Interpolative decomposition for covariance matrices

In some applications we only have the covariance matrix $\mathbf{\Sigma}$. To define groups, we can compute the Cholesky $\mathbf{\Sigma} = \mathbf{A}^t\mathbf{A}$ where $\mathbf{A}$ is an upper triangular matrix, and apply the ID procedure to $\mathbf{A}$. This produces the following algorithm

1. Let $\mathbf{\Sigma}$ be the correlation matrix and S be the set of selected representatives
2. Compute Cholesky $\mathbf{\Sigma} = \mathbf{A}^t\mathbf{A}$ where $\mathbf{A}$ is upper triangular
3. Apply interpolative decomposition to $\mathbf{A} = \mathbf{CZ}$ where $\mathbf{C}$ is a column permutation of $\mathbf{A}$ and $\mathbf{Z}$ is some interpolation matrix
4. Choose S to be first k columns of $\mathbf{C}$
5. Increase k until: for all $j \notin S, max(c_j) < 0.25$ where $c_j = \Sigma_{jj} - \mathbf{\Sigma}_{j,S}\mathbf{\Sigma}_{SS}^{-1}\mathbf{\Sigma}_{S,j}$

Here the constant c_j is the sum of squares residuals of regression the j variant on those in S. To see why, let $\mathbf{a}_j$ denote the jth column of $\mathbf{A}$, $\mathbf{A}_S$ denote the columns of $\mathbf{A}$ that are in S, then

$$\begin{aligned} c_j &= (\mathbf{a}_j - \mathbf{A}_S\hat{\boldsymbol{\beta}})^t(\mathbf{a}_j - \mathbf{A}_S\hat{\boldsymbol{\beta}}) \\ &= (\mathbf{a}_j - \mathbf{A}_S(\mathbf{A}_S^t\mathbf{A}_S)^{-1}\mathbf{A}_S\mathbf{a}_j)^t(\mathbf{a}_j - \mathbf{A}_S(\mathbf{A}_S^t\mathbf{A}_S)^{-1}\mathbf{A}_S\mathbf{a}_j) \\ &= \mathbf{a}_j^t\mathbf{a}_j - 2\mathbf{a}_j^t\mathbf{A}_S^t(\mathbf{A}_S^t\mathbf{A}_S)^{-1}\mathbf{A}_S\mathbf{a}_j + \mathbf{a}_j^t\mathbf{A}_S^t(\mathbf{A}_S^t\mathbf{A}_S)^{-1}\mathbf{A}_S^t\mathbf{A}_S(\mathbf{A}_S^t\mathbf{A}_S)^{-1}\mathbf{A}_S\mathbf{a}_j \\ &= \mathbf{a}_j^t\mathbf{a}_j - \mathbf{a}_j^t\mathbf{A}_S^t(\mathbf{A}_S^t\mathbf{A}_S)^{-1}\mathbf{A}_S\mathbf{a}_j \\ &= \Sigma_{jj} - \mathbf{\Sigma}_{j,S}\mathbf{\Sigma}_{S,S}^{-1}\mathbf{\Sigma}_{S,j}. \end{aligned}$$

At first glance, step (5) requires computing $\mathbf{\Sigma}_{S,S}^{-1}$ afresh. However, assuming we have its expression at the previous k, we can compute $\mathbf{\Sigma}_{S,S}^{-1}$ by taking advantage of the block-matrix inversion formula which circumvents inverting a $k \times k$ matrix.

S5.3 Selecting group-key variables to exploit conditional independence

Algorithm A2 provides a heuristic algorithm for identifying group-key conditional independence described in Definition 1, somewhat motivated by the algorithms presented in Sood and Hastie (2023). It proceeds as follows.

Firstly, we can think of $\mathcal{A}_\gamma^\star$ as *key* variables selected for group γ, and $\mathcal{A}_\gamma^\dagger$ are the non-selected variables (i.e. redundant variables) in group γ. Consider variant j belonging in group γ but not yet selected as a key. The quantity

$$\eta_j = \mathbf{\Sigma}_{j,\mathcal{A}_\gamma^\star}\mathbf{\Sigma}_{\mathcal{A}_\gamma^\star,\mathcal{A}_\gamma^\star}^{-1}\mathbf{\Sigma}_{\mathcal{A}_\gamma^\star,j}$$

is the explained sum of squares $\|\hat{\mathbf{x}}_j\|^2$ when regressing the jth variant on the key variants in group γ. To see why, let $\mathbf{H}$ denote the hat matrix, we have

$$\boldsymbol{\Sigma}_{j,\mathcal{A}_\gamma^\star}\boldsymbol{\Sigma}_{\mathcal{A}_\gamma^\star,\mathcal{A}_\gamma^\star}^{-1}\boldsymbol{\Sigma}_{\mathcal{A}_\gamma^\star,j} = \mathbf{x}_j^t\mathbf{X}_{\mathcal{A}_\gamma^\star}(\mathbf{X}_{\mathcal{A}_\gamma^\star}^t\mathbf{X}_{\mathcal{A}_\gamma^\star})^{-1}\mathbf{X}_{\mathcal{A}_\gamma^\star}^t\mathbf{x}_j = \mathbf{x}_j^t\mathbf{H}\mathbf{x}_j = \mathbf{x}_j^t\mathbf{H}^2\mathbf{x}_j = \|\mathbf{H}\mathbf{x}_j\|^2 = \|\hat{\mathbf{x}}_j\|^2.$$

Analogously, the quantity $\zeta_j = \boldsymbol{\Sigma}_{j,-\mathcal{A}_\gamma^\dagger}\boldsymbol{\Sigma}_{-\mathcal{A}_\gamma^\dagger,-\mathcal{A}_\gamma^\dagger}^{-1}\boldsymbol{\Sigma}_{-\mathcal{A}_\gamma^\dagger,j}$ is the explained sum of squares when regressing $\mathbf{x}_j$ on the key variables plus all variants outside group γ. Thus, $\frac{\eta_j}{\zeta_j}$ is the proportion of variation explained by $\mathcal{A}_\gamma^\star$ vs variation explained by $\mathcal{A}_\gamma^\star \cup -\mathcal{A}_\gamma$, which is supposed to be 1 under the conditional independence assumption in Definition 1. The algorithm proceeds by increasing the number of key variables until

$$\underset{j\notin\mathcal{A}_\gamma^\star, j\in\mathcal{A}_\gamma}{\text{mean}} \frac{\boldsymbol{\Sigma}_{j,\mathcal{A}_\gamma^\star}\boldsymbol{\Sigma}_{\mathcal{A}_\gamma^\star,\mathcal{A}_\gamma^\star}^{-1}\boldsymbol{\Sigma}_{\mathcal{A}_\gamma^\star,j}}{\boldsymbol{\Sigma}_{j,-\mathcal{A}_\gamma^\dagger}\boldsymbol{\Sigma}_{-\mathcal{A}_\gamma^\dagger,-\mathcal{A}_\gamma^\dagger}^{-1}\boldsymbol{\Sigma}_{-\mathcal{A}_\gamma^\dagger,j}} \quad \geq \quad c \in [0,1].$$

When this condition in not met, we search through all variables j in $\mathcal{A}_g^\dagger$ and find the one that can explain the most amount of the remaining variation, elect that variant as a member of $\mathcal{A}_g^\star$, and repeat the process.

Algorithm A2 Searching for covariates to explain most of between-group dependencies with respect to the group structure $\{\mathcal{A}_\gamma : \gamma \in [g]\}$.

Input: Random variables X, covariance matrix $\boldsymbol{\Sigma}$ and the threshold $c \in [0,1]$ (the target proportion of between-group dependencies to be explained).
for $\gamma \in [g]$ **do**
  Initialize $\mathcal{A}_\gamma^\star = \emptyset$ and $\mathcal{A}_\gamma^\dagger = \mathcal{A}_\gamma$.
  for $j \in \mathcal{A}_\gamma^\dagger$ **do**
    Compute $\eta_j = 0$ and $\zeta_j = \boldsymbol{\Sigma}_{j,-\mathcal{A}_\gamma^\dagger}\boldsymbol{\Sigma}_{-\mathcal{A}_\gamma^\dagger,-\mathcal{A}_\gamma^\dagger}^{-1}\boldsymbol{\Sigma}_{-\mathcal{A}_\gamma^\dagger,j}$.
  end for
  while $\sum_{j\in\mathcal{A}_\gamma^\dagger}\eta_j/\zeta_j < c|\mathcal{A}_\gamma^\dagger|$ **do**
    Find j^* via

$$j^* = \arg\max_{j\in\mathcal{A}_\gamma^\dagger} \sum_{j^\dagger\in\mathcal{A}_\gamma^\dagger\setminus\{j\}} \boldsymbol{\Sigma}_{j^\dagger,\mathcal{A}_\gamma^\star\cup\{j\}}\boldsymbol{\Sigma}_{\mathcal{A}_\gamma^\star\cup\{j\},\mathcal{A}_\gamma^\star\cup\{j\}}^{-1}\boldsymbol{\Sigma}_{\mathcal{A}_\gamma^\star\cup\{j\},j^\dagger}.$$

    Update $\mathcal{A}_\gamma^\star \leftarrow \mathcal{A}_\gamma^\star \cup \{j^*\}$ and $\mathcal{A}_\gamma^\dagger \leftarrow \mathcal{A}_\gamma^\dagger \setminus \{j^*\}$.
    for $j \in \mathcal{A}_\gamma^\dagger$ **do**
      Update $\eta_j = \boldsymbol{\Sigma}_{j,\mathcal{A}_\gamma^\star}\boldsymbol{\Sigma}_{\mathcal{A}_\gamma^\star,\mathcal{A}_\gamma^\star}^{-1}\boldsymbol{\Sigma}_{\mathcal{A}_\gamma^\star,j}$ and $\zeta_j = \boldsymbol{\Sigma}_{j,-\mathcal{A}_\gamma^\dagger}\boldsymbol{\Sigma}_{-\mathcal{A}_\gamma^\dagger,-\mathcal{A}_\gamma^\dagger}^{-1}\boldsymbol{\Sigma}_{-\mathcal{A}_\gamma^\dagger,j}$
    end for
  end while
end for
Output: $\{\mathcal{A}_\gamma^\star : \gamma \in [g]\}$.

S6 Practical strategy for estimating $\hat{\boldsymbol{\mu}}$ and $\hat{\boldsymbol{\Sigma}}$

In practice, we are often given individual level data $\mathbf{X} \in \mathbb{R}^{n\times p}$ and asked to generate second-order model-X knockoffs. This relies on obtaining suitable estimates for $\hat{\boldsymbol{\mu}} \in \mathbb{R}^p$ and $\hat{\boldsymbol{\Sigma}} \in \mathbb{R}^{p\times p}$. We

always use the sample mean $\mu_j = \sum_i X_{ij}/n$ to estimate $\hat{\boldsymbol{\mu}}$, but when $p \gg n$, the sample covariance or maximum-likelihood based estimators are ill-suited to estimate $\hat{\boldsymbol{\Sigma}}$ (Schäfer and Strimmer 2005).

In `Knockoffs.jl`, we use a linear shrinkage estimator of the form

$$\hat{\boldsymbol{\Sigma}} = (1-\lambda)\mathbf{S} + \lambda \mathbf{F}$$

where $\mathbf{F}$ is a *target* matrix of appropriate dimensions, $\lambda \in [0,1]$ is a shrinkage intensity, and $\mathbf{S}$ is the sample covariance estimator. Several choises of $\mathbf{F}$ are possible (Schäfer and Strimmer 2005). By default, we use the common choice $F_{ii} = S_{ii}$ and $F_{ij} = 0$ and compute λ via Lediot-Wolf shrinkage (Ledoit and Wolf 2003). These features, including more choices for $\mathbf{F}$ and estimating λ, are implemented in the Julia package `CovarianceEstimation.jl`.

S7 GhostKnockoff pipeline on the Pan-UKB panel

S7.1 Summary statistics on Pan-UKB matrices

Here we provide some summary statistics on the Pan-UKB matrices featured in our real data analysis of Albuminuria GWAS data. Figures S1 provides some summary statistics on the 1703 independent blocks, restricting to the genotyped SNPs. Most blocks have around 500 SNPs per block, while possessing group sizes of up to 300 variables per group. After the identification of group-key variables by running Algorithm (A2), the number of (key) variables per block is reduced to around 200 SNPs per block, and the maximum group size becomes at most 4. Thus, the identification of group-key variables significantly reduced the number of parameters that need to be optimized ($> 90,000$ to 16 for the largest group) for GWAS summary statistics analysis.

S7.2 Regularizations applied to Pan-UKB LD matrices

Regularization to the LD matrices were done in multiple steps.

First, given the downloaded $\hat{\boldsymbol{\Sigma}}_{\text{PanUK}}$, we identified approximately independent blocks of SNPs with the `ldetect` software (Berisa and Pickrell 2016). A total of 1703 blocks $\hat{\boldsymbol{\Sigma}}_1, ..., \hat{\boldsymbol{\Sigma}}_{1703}$ of size varying between $\sim 10^2$ and $\sim 10^3$ were identified. Next, after reading the relevant portions of the data in $\hat{\boldsymbol{\Sigma}}_i$, we force $\hat{\boldsymbol{\Sigma}}_i$ to be positive definite by computing its eigen-decomposition and setting all eigenvalues to be $\geq 10^{-5}$. Next, we solve for $\mathbf{S}_1, ..., \mathbf{S}_{1703}$ by applying Algorithm 2 to each regularized $\hat{\boldsymbol{\Sigma}}_i$ separately. This action delivers

$$\mathbf{G}_{\mathbf{S}_i} = \begin{bmatrix} \boldsymbol{\Sigma}_i & \boldsymbol{\Sigma}_i - \mathbf{S}_i & \cdots & \boldsymbol{\Sigma}_i - \mathbf{S}_i \\ \boldsymbol{\Sigma}_i - \mathbf{S}_i & \boldsymbol{\Sigma}_i & \cdots & \boldsymbol{\Sigma}_i - \mathbf{S}_i \\ \vdots & \vdots & \ddots & \vdots \\ \boldsymbol{\Sigma}_i - \mathbf{S}_i & \cdots & \cdots & \boldsymbol{\Sigma}_i \end{bmatrix} \in \mathbb{R}^{p_i(m+1)\times p_i(m+1)}.$$

for each $i = \{1, ..., 1703\}$ which represents the covariance matrix for $(\mathbf{z}_i, \tilde{\mathbf{z}}_{i1}, ..., \tilde{\mathbf{z}}_{im})$. This allows us to assemble the overall covariance matrix

$$\mathbf{A}_{\text{unregularized}} = \begin{bmatrix} \mathbf{G}_{\mathbf{S}_1} & & \\ & \ddots & \\ & & \mathbf{G}_{\mathbf{S}_{1703}} \end{bmatrix} \in \mathbb{R}^{p(m+1)\times p(m+1)}.$$

Finally, we set

$$\mathbf{A} = \mathbf{A}_{\text{unregularized}} + 0.01\mathbf{I} \tag{S.18}$$

and plug $\mathbf{A}$ into the lasso solver (Yang and Hastie 2023).

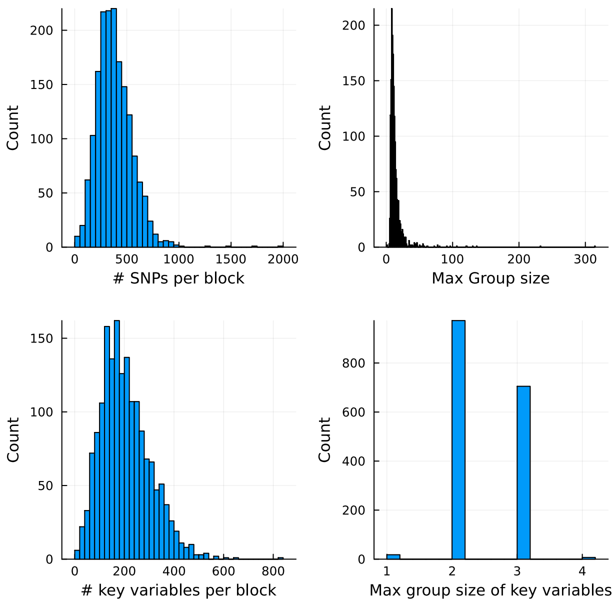

Figure S1: Summary statistics for the Pan-UKBB LD panels after partitioning into 1703 quasi-independent blocks. We restrict each region to typed SNPs present on the UKB-genotyping array. For each region, we compute the number of SNPs, number of representatives selected, maximum group size in each region (determined by average linkage hierarchical clustering with correlation cutoff 0.5), and the maximum group size in each region after selecting representatives.

S7.3 Visualization of group knockoff exchangeability with different thresholds for selecting group-key variables

Figure S2 gives an illustration of exchangeability measures between knockoffs and original data for 4 randomly selected genomic regions. On the x-axis, we plot Σ_{ij} which represents $corr(X_i, X_j)$, and on the y-axis we plot $\Sigma_{ij} - S_{ij}$ which represents $corr(X_i, \tilde{X}_j)$. When (i, j) belong to different groups, $\tilde{X}_j$ should be exchangeable with X_j in the sense that $corr(X_i, X_j) = corr(X_i, \tilde{X}_j)$, which will result in a point lying perfectly on the diagonal. Thus, decreasing the threshold value c has the effect of producing less exchangeable knockoffs as a consequence of selecting fewer key variables per group. Since our block-diagonal approximation seems to produce slightly conservative FDR for regular group knockoffs, small deviations do not violate empirical FDR.

S7.4 Tuning Lasso hyperparameter without individual level data

Optimizing the Lasso objective typically involves cross-validating for the hyper parameter λ. In the absence of individual level data, we adopt the pseudo-summary statistics approach (Mak et al. 2017; Zhang et al. 2021). Given $(\mathbf{r}, \tilde{\mathbf{r}})$ where $\mathbf{r} = \frac{1}{\sqrt{n}}\mathbf{z}$ and $\tilde{\mathbf{r}} = \frac{1}{\sqrt{n}}\tilde{\mathbf{z}}$ and the matrix $\mathbf{A}$ in equation (S.18), we create training and validation summary statistics

$$\begin{aligned}\begin{pmatrix}\mathbf{r}\\ \tilde{\mathbf{r}}\end{pmatrix}_t &= \begin{pmatrix}\mathbf{r}\\ \tilde{\mathbf{r}}\end{pmatrix} + \sqrt{\frac{n_v}{n \times n_t}} N(0, \mathbf{A}),\\ \begin{pmatrix}\mathbf{r}\\ \tilde{\mathbf{r}}\end{pmatrix}_v &= \frac{1}{n_v}\left[n\begin{pmatrix}\mathbf{r}\\ \tilde{\mathbf{r}}\end{pmatrix} - n_t\begin{pmatrix}\mathbf{r}\\ \tilde{\mathbf{r}}\end{pmatrix}_t\right].\end{aligned}$$

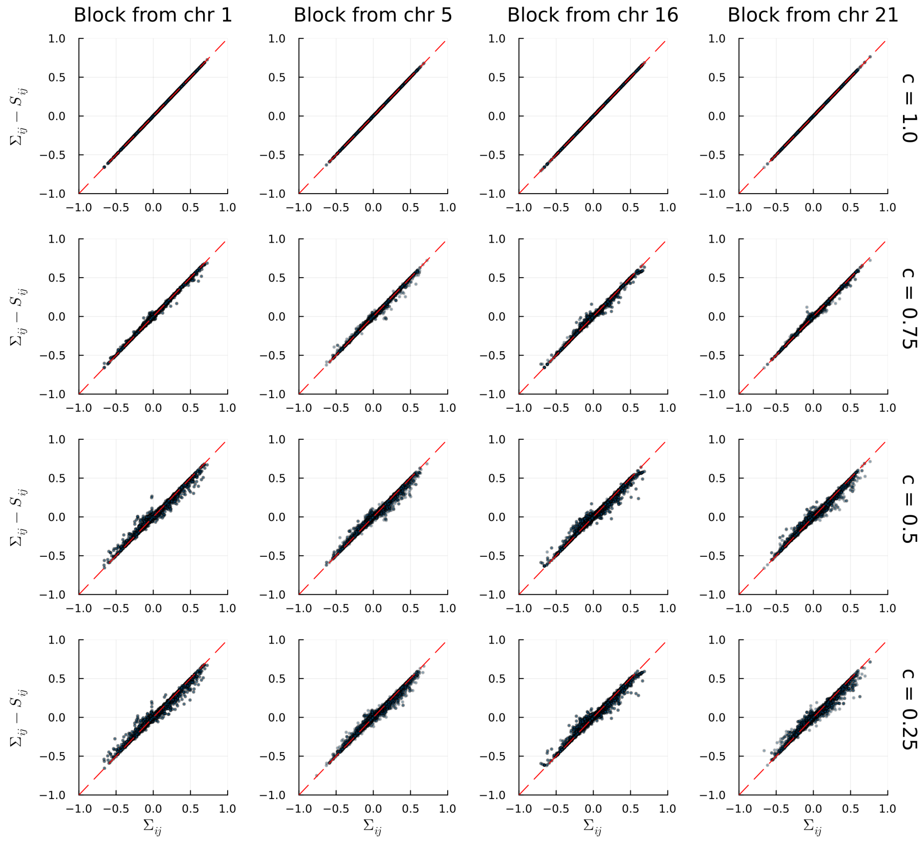

Figure S2: Visualization of group knockoff exchangeability by applying Algorithms 2 and (A2) on the Pan-UKB data. Each column corresponds to a randomly selected LD block, each row chooses a different threshold for selecting representatives, and each i, j pair are indices from different groups. Here Σ_{ij} represents $corr(X_i, X_j)$, while $\Sigma_{ij} - S_{ij}$ represents $corr(X_i, \tilde{X}_j)$. A threshold of 1 includes all variables within groups. The 4 selected regions contain 247, 511, 333, and 431 SNPs partitioned into 122, 279, 177, and 234 groups, respectively.

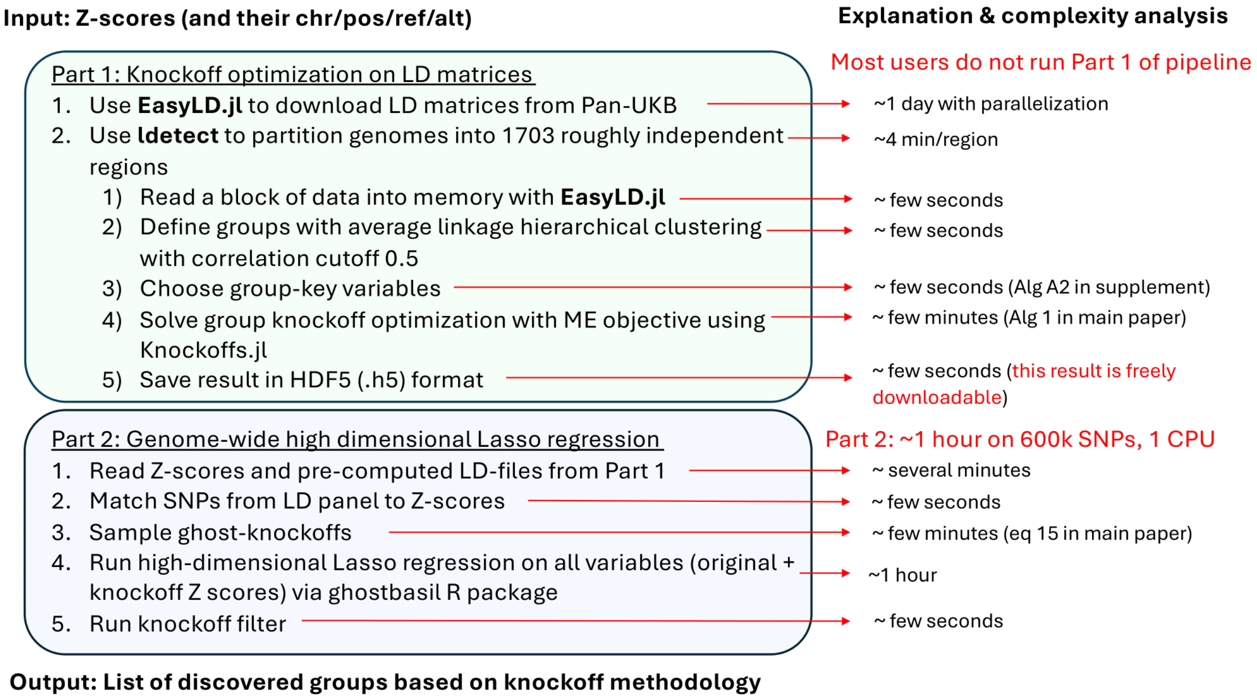

Figure S3: High-level summary of the ghost-knockoff pipeline featured in this paper.

If the sample size is n, we let $n_t = 0.8n, n_v = 0.2n$, and choose λ that maximizes

$$f(\lambda) = \frac{\boldsymbol{\beta}_\lambda^t \begin{pmatrix} \mathbf{r} \\ \tilde{\mathbf{r}} \end{pmatrix}_v}{\sqrt{\boldsymbol{\beta}_\lambda^t \mathbf{A} \boldsymbol{\beta}_\lambda}},$$

where $\boldsymbol{\beta}_\lambda$ is trained on the training summary statistics.

S7.5 Summary of Ghost Knockoff pipeline

Figure S3 provides a high-level overview of the current Ghost Knockoff pipeline when applied on the Albuminuria GWAS data. The only user-provided input are Z-scores, and the output is the list of discovered groups according to the knockoff methodology. The pipeline is divided into 2 parts, where the algorithms and software developed in the current paper mainly work on part 1 of this pipeline. Because the output of part 1 contains no individual level data, it can be freely distributed online. In our companion paper (He et al. 2024), we further develop a software pipeline that allows users to carry out the entire analysis with little technical requirements.

S8 Additional simulations

S8.1 The advantage of group-based inference

Although group knockoffs and regular model-X knockoffs test different hypotheses, it is still of practical interest to determine the porportion of true signals being discovered. In this section, we

perform a basic simulation featuring a symmetric Toeplitz matrix

$$\mathbf{\Sigma} = \begin{bmatrix} 1 & \rho & \rho^2 & \cdots & \rho^{p-1} \\ \rho & 1 & \rho & \cdots & \\ \rho^2 & & & & \\ \vdots & & & \ddots & \vdots \\ \rho^{p-1} & & & \rho & 1 \end{bmatrix}_{p \times p} \tag{S.19}$$

where correlation between features $\rho = 0.9$ and $p = 200$. The response $\mathbf{y}$ is simulated as $\mathbf{y} = \mathbf{X}\boldsymbol{\beta} + \mathcal{N}(\mathbf{0}, \mathbf{I})$ with $k = 10$ causal effects randomly chosen across the p features with effect size $\beta_j = \pm 0.25$. Then we generate model-X group and ungrouped knockoffs and compute the proportion of signals discovered and the grouped/ungrouped FDR based on the Lasso coefficient difference statistic. The result is visualized in Figure S4. Due to the high-correlation between neighboring features, regular model-X knockoffs discovers much less causal features than group knockoffs.

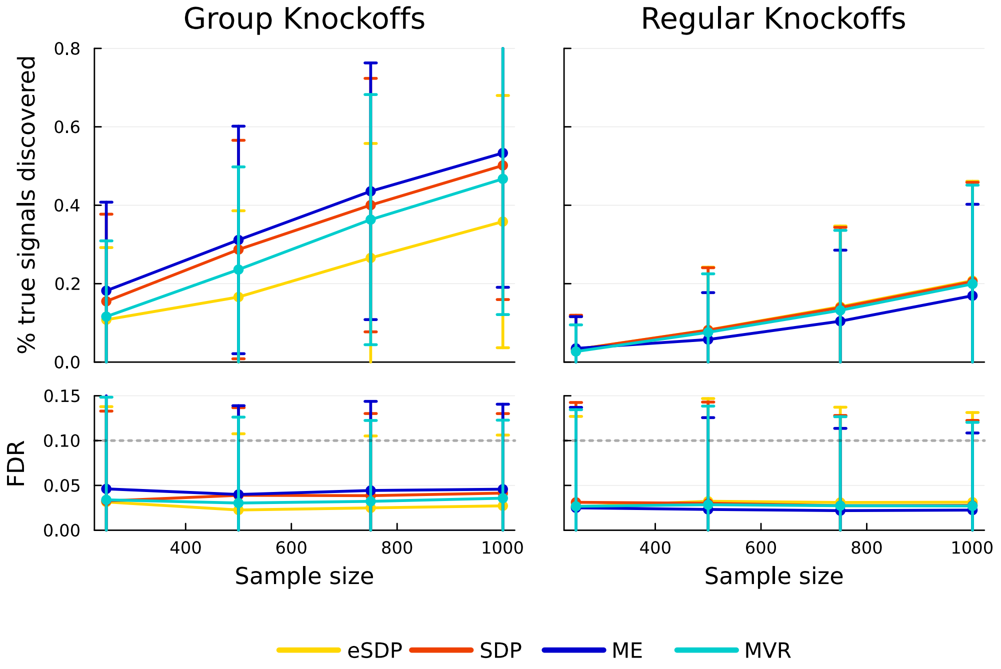

Figure S4: Group vs regular model-X knockoffs. When features are highly correlated, group knockoffs discover more causal features.

S8.2 Simulation details for section 6.1.1 in main text

Here we describe the 5 main covariance matrices used in section 6.1.1 of the main text.

Block cov. This simulation roughly follows Dai and Barber 2016. In this basic setting, we define 200 contiguous blocks each of size 5, and the covariance $\mathbf{\Sigma}$ is set as

$$\Sigma_{ij} = \begin{cases} 1 & i = j \\ \rho & (i,j) \text{ in the same block} \\ \gamma\rho & (i,j) \text{ in different blocks} \end{cases}$$

where $\rho = 0.75$ and $\gamma = 0.25$. Note that the blocks corresponds to "true" group structure, but we do not leverage this, defining groups membership empirically as described above.

ER (Erdos-Renyi). This simulation roughly follows the clustered ER simulation of Li and Maathuis 2021. Here we define 100 contiguous blocks $\mathbf{\Omega}^1, ..., \mathbf{\Omega}^{100}$ each of size 10×10. For each block k, $\mathbf{\Omega}^k$ is simulated from an Erdos-Renyi graph by letting $\Omega^k_{ii} = 1$ and $\Omega^k_{ij} = \Omega^k_{ji} = \omega_{ij}\phi_{ij}$ where $\omega_{ij} \sim \pm\text{Uniform}(0.3, 0.9)$ and $\phi_{ij} \sim \text{Bernoulli}(0.1)$. Intuitively, if features i and j are in the same block, then with with probability 0.1 they will have correlation ω_{ij}. We let $\mathbf{V} = \text{diag}(\mathbf{\Omega}^1, ..., \mathbf{\Omega}^{100})$ and define

$$\mathbf{\Sigma} = \mathbf{V} + (|\lambda_{\min}(\mathbf{V})| + 0.1)\mathbf{I}.$$

ER(prec). Similar to the ER setting, we define

$$\mathbf{\Sigma} = (\mathbf{V} + (|\lambda_{\min}(\mathbf{V})| + 0.1)\mathbf{I})^{-1}.$$

AR1. This simulation follows Spector and Janson 2022. In the AR(1) setting, we simulate

$$\Sigma_{ij} = \begin{cases} 1 & i = j \\ \exp\left\{-|\sum_{k=2}^{i} \log(\rho_k) - \sum_{k=2}^{j} \log(\rho_k)|\right\} & i \neq j. \end{cases}$$

We sample $\rho_j \sim \text{Beta}(3, 1)$ to generate a setting where neighboring features are highly correlated. To ensure positive definiteness, if $\lambda_{min}(\mathbf{\Sigma}) < 0.001$, we add $(0.001 - \lambda_{min}(\mathbf{\Sigma}))\mathbf{I}_p$ to $\mathbf{\Sigma}$, where $\lambda_{min}(\mathbf{\Sigma})$ computes the minimum eigenvalue of $\mathbf{\Sigma}$, and rescale back to a correlation matrix.

AR1(corr). Here, $\mathbf{\Sigma}$ is the same as in AR(1) above, but the true coefficient vectors are simulated such that all k non-zero β_js are placed contiguously. This simulation aims to capture the genetic reality that many disease variants are clustered tigthly together in the same LD block (e.g. residing in the same gene) but each exerts an independent effect on disease outcome.

S8.3 Additional simulations using the Pan-UKB panel

Here we use covariance matrices extracted from Pan-UKB (Pan-UKB team 2020) to conduct simulations. Given the pre-processed data $\mathbf{\Sigma}_1, ..., \mathbf{\Sigma}_{1703}$ described in section 5.1 of the main text, we randomly select with replacement 500 covariances $\mathbf{\Sigma}_i$ and generate corresponding design matrices $\mathbf{X}_i \in \mathbb{R}^{n \times p_i}$ with $n = 250$ independent samples. For each replicate, we assume $k = 10$ non-zero effects, with $\beta_j \sim N(0, 0.5)$ where the non-zero β_js are randomly chosen across the p_i features. Then the response is simulated as $\mathbf{y}_i = \mathbf{X}_i\boldsymbol{\beta}_i + N(\mathbf{0}, \mathbf{I}_{p\times p})$ as before. To explore to which extend the group-key conditional independence hypothesis is appropriate for these matrices, we first define groups with average linkage hierarchical clustering with correlation cutoff 0.5, then identified key variables with Algorithm A2 and considering four different levels for $c \in \{0.25, 0.5, 0.75, 1.0\}$. Note that $c = 1$ is equivalent to not using the conditional independence assumption. Importantly, it is possible for a causal variant to not be selected as a key variable. We ran one simulation for each of the selected covariance matrices, and averaged the power/FDR across 500 simulations.

Figure S5 summarizes power and FDR level achieved in a simulation constructed starting from genetic variance-covariance matrices. In general, ME have the best power, followed by MVR, SDP, and finally eSDP. Utilizing conditional independence offers slightly better power than regular group knockoffs. Group-FDR can be controlled on GWAS data when the selected representatives explain at least 50% of variation within groups, while a threshold of 25% leads to slightly inflated empirical FDR.

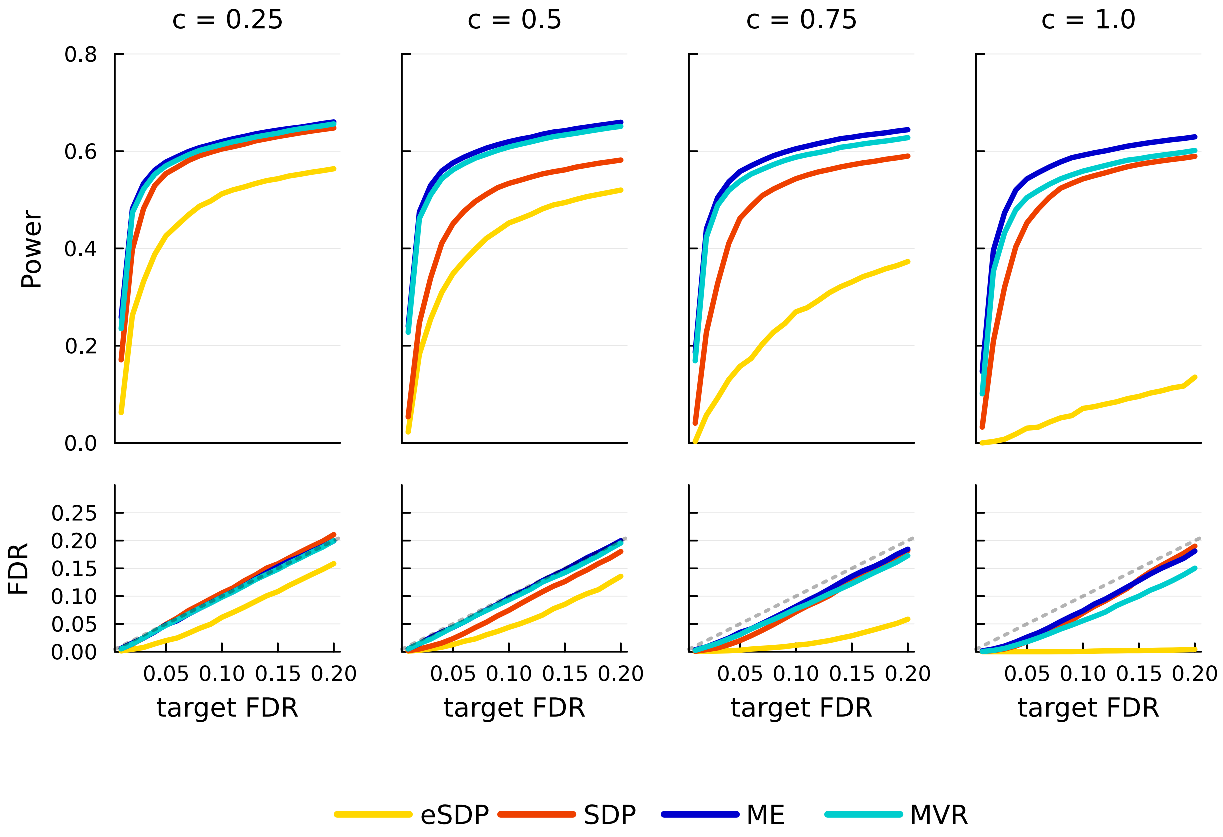

Figure S5: Power/FDR comparison of group knockoffs utilizing conditional independence assumption using 500 different covariance matrices extracted from the Pan-UKBB LD panel. FDR is controlled when the threshold c for selecting group representatives exceeds 0.5. Note that a threshold of 1 corresponds to regular group knockoffs.

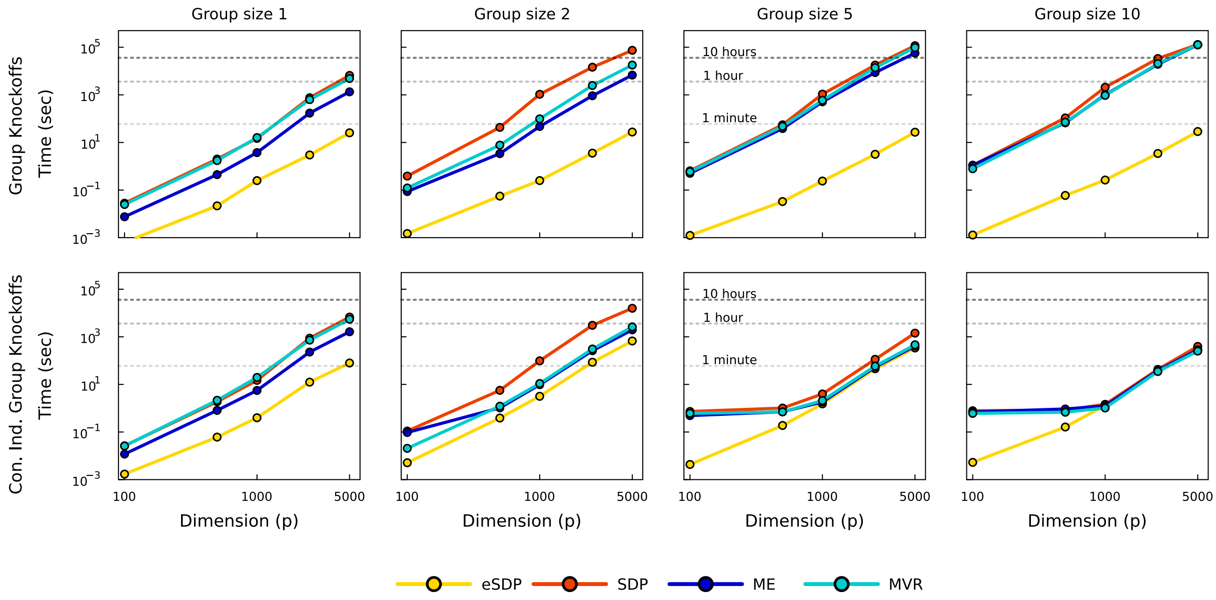

Figure S6: Runtime comparison of group knockoffs solvers (top) and when conditional independence assumption are utilized with threshold $c = 0.5$ (bottom). The gray dashed lines indicate 1-minute, 1-hour, and 10-hour marks. Each data point plotted is an average over 25 replicates. $\mathbf{\Sigma}$ is drawn from the AR1 model. A group size of 1 corresponds to regular (non-grouped) knockoffs. The convergence tolerance was set to 0.0001.

S8.4 Additional runtime comparisons of group knockoff solver vs utilization of conditional indepenence

In the main text, timing results for Figure 1 are listed in Table 1. Figure S6 investigates the computational efficiency of our proposed algorithms in more detail. Two important parameters, namely, number of features p and group sizes, are varied according to what seems appropriate for the GWAS analysis example. Data are generated according to the AR1 setting supplemental section S8.2, where the size of each group is fixed between 1 (no group structure) and 10 (each group contains 10 contiguous variables). When conditional independence assumption is utilized, we let $c = 0.5$. Equi-correlated constructions (eSDP) offer the best speed due to its convenient closed form formula (Dai and Barber 2016). Otherwise, ME solvers tend to run faster than MVR or SDP solvers. We find that SDP solver requires more iterations to converge, making it the slowest method in general. When comparing MVR and ME solvers, both require the same number of Cholesky updates, but each ME iteration requires only solving 1 forward-backward equation, while MVR requires 3. We wrote an efficient vectorized routine for performing Cholesky updates, and we use LAPACK (Anderson et al. 1999) to perform required forward-backward solves. Careful benchmarks reveal the latter step constitute approximately 90% of compute time, which explains the timing difference between MVR and ME solvers.

S8.5 Marginal correlation as feature importance statistics

Figure 1 in the main text explored the performance of our proposed algorithms using Lasso coefficient difference statistic as feature importance scores. In practice, marginal association are used more often in genome-wide association studies (GWAS). Therefore, here we use squared marginal

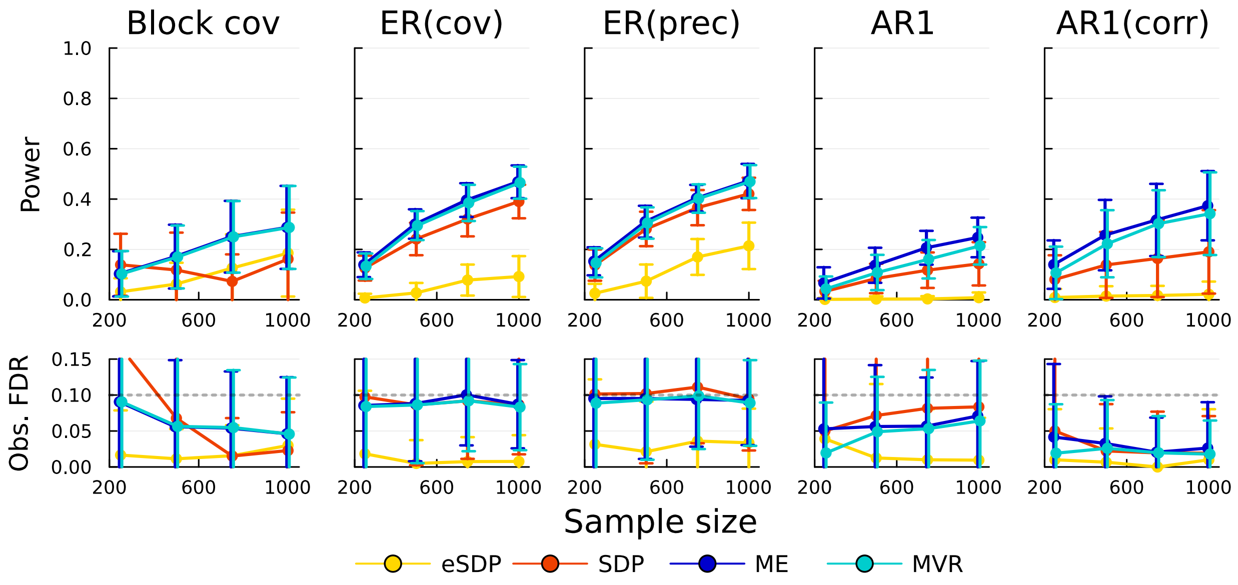

Figure S7: Power/FDR comparison of Equi, SDP, MVR, and ME group knockoffs on simulated covariances. This is the same simulation as Figure 1 of the main text, except we use the squared marginal Z-statistics as feature importance scores.

correlation as importance measure. For each feature i, we compute its knockoff scores as

$$T_i = \frac{1}{n}(\mathbf{x}^t \mathbf{y})^2, \quad \tilde{T}_i = \frac{1}{n}(\tilde{\mathbf{x}}^t \mathbf{y})^2,$$

where both $\mathbf{y}$ and $\mathbf{x}_i$ have been standardized to mean 0 variance 1. Then the feature importance score for each group γ can be computed as $Z_\gamma = \sum_{i \in \mathcal{A}_\gamma} T_i$. The result is presented in Figure S7.

S8.6 Distribution of S_{ij} and minimum eigenvalues of $\mathbf{G}_\mathbf{S}$

Other than comparing power and FDR, one often employs heuristics to assess the quality of knockoff solvers. One commonly used metric is to look at the distribution of the diagonal elements of $\mathbf{S}$, where larger values are considered "better". As discussed in Spector and Janson (2022), this heuristic could fail and very large $\mathbf{S}$ values can imply *lower* power.

To verify this phenomenon, here we compare the four group knockoff solvers and plot the distribution of non-zero entries of $\mathbf{S}$ and the minimum eigenvalue of $\mathbf{G}_\mathbf{S}$ in Figure S8. The simulation here replicates the ER(prec) simulation in section S8.2 except we generate $m = 1$ knockoff for simplicity. One observes that SDP solvers do produce rather large $\mathbf{S}$ values, with a heavy tail for values closer to 1. However, we know from Figure 1 of main text that SDP solvers exhibit worse power than ME/MVR in this example. Furthermore, the minimum eigenvalue of $\mathbf{G}_{\mathbf{S}_{\text{SDP}}}$ appears much closer to 0 than that of MVR/ME solvers, causing $\mathbf{G}_{\mathbf{S}_{\text{SDP}}}$ to become numerically singular. Thus, the columns of the concatenated design matrix $[\mathbf{X}\tilde{\mathbf{X}}]$ are linearly dependent, exemplifying the reconstruction effect discussed in Spector and Janson (2022). In both plots, MVR and ME results are quite similar, as they ought to be since L_{MVR} and L_{ME} are very similar in the Gaussian case. This explains their comparable power and FDR.

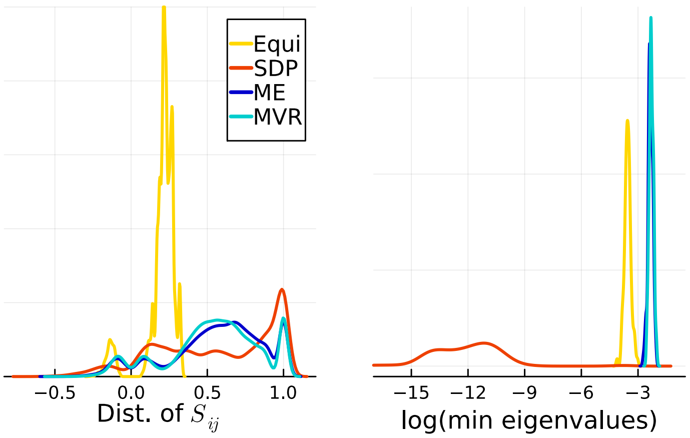

Figure S8: Comparison between methods for generating Gaussian knockoffs. On the left, we plot the distribution of non-zero values in $\mathbf{S}$ matrix. On the right, we plot the log of the minimum eigenvalue of the covariance matrix $\mathbf{G_S}$

S9 Albuminuria GWAS - in depth analysis

S9.1 Result using eSDP knockoffs

To further explore the improved power of our new group-knockoff algorithms, we performed another analysis of our Albuminuria GWAS using the eSDP construction of Dai and Barber (2016). Here we applied the same GWAS pipeline as outlined in section 5 of the main text, except we substitute the eSDP solver for the knockoff optimization step. The resulting manhattan plot is displayed in Figure S9. After knockoff filter, eSDP knockoffs identified 4 independent SNPs after LD pruning. Compare this to Figure S10 in the main paper, one sees that ME group knockoffs can identify 35 signals, including the 4 signals identified by eSDP. This example showcases the enhanced power of new algorithms described in the current paper.

S9.2 Manhattan plot for Marginal association test of Albuminuria

In the main text, we presented the knockoff Manhattan plot. It is useful to contrast that with the standard Manhattan plot applied on marginal p-values. This result is shown in Figure S10.

S9.3 Full list of discoveries from Albuminuria GWAS

Table S1 list the SNPs discovered by our second-order Ghost knockoff analysis of Albuminuria GWAS. Note that for each discovered group, we only list the group-key variant with the most significant marginal Z score. The full result, including all SNPs within groups and non-significant SNPs, can be accessed from our online GitHub page. To compare against previous studies, we manually searched each discovered variant against the NHGRI-EBI GWAS catalog (MacArthur et al. 2017) and list genes that have been mapped to the SNP.

S9.4 Functional annotations for additionally discovered SNPs

Table S2 examines variants discovered by our method but missed by traditional GWAS, using the web tool FAVOR (Zhou et al. 2023). Notably, variant rs33950747 causes a non-synonymous mutation. The mutation changes arginine to glutamine at position 408 (p.R408Q) in NPHS1,

rsID	Variant	AF	Z	p-value	W	Mapped gene
rs12032996	1:33454985:G:A	0.162	-6.477	9.33E-11	0.0028	PHC2,TLR12P
rs2154319	1:41280098:T:C	0.218	-5.121	3.04E-07	0.0023	FOXO6,SCMH1
rs12727019	1:47495360:T:C	0.087	-6.131	8.75E-10	0.0036	
rs7540974	1:47503824:G:A	0.881	6.097	1.08E-09	0.0035	
rs471608	1:47519020:T:G	0.185	7.271	3.57E-13	0.0045	
rs934287	2:202843584:A:G	0.813	6.688	2.26E-11	0.003	ICA1L
rs1047891	2:210675783:C:A	0.316	-6.729	1.71E-11	0.0053	CPS1
rs4665972	2:27375230:T:C	0.607	-6.858	6.96E-12	0.0047	SNX17
rs17026396	2:85532004:T:C	0.43	-6.248	4.15E-10	0.0024	RPSAP22,PARTICL
rs1077216	3:46850671:C:T	0.069	5.396	6.81E-08	0.0022	MYL3
rs7670121	4:148207444:A:G	0.24	5.941	2.84E-09	0.0035	NR3C2
rs4109437	4:189848068:G:A	0.038	10.685	1.20E-26	0.0112	FRG1-DT
rs6831256	4:3471412:A:G	0.421	5.251	1.51E-07	0.0022	DOK7
rs10032549	4:76476862:A:G	0.537	-6.013	1.82E-09	0.0037	SHROOM3
rs1465405	5:148736639:T:G	0.248	-5.348	8.90E-08	0.0026	
rs4865796	5:53976834:G:A	0.692	5.764	8.20E-09	0.0027	ARL15
rs3776051	5:64993329:A:G	0.229	6.132	8.70E-10	0.0039	CWC27
rs6569648	6:130027974:C:T	0.759	5.379	7.50E-08	0.0027	L3MBTL3
rs9472138	6:43844025:C:T	0.291	5.156	2.52E-07	0.0026	VEGFA,LINC02537
rs11983745	7:100626055:T:C	0.2	-5.137	2.79E-07	0.0023	
rs4410790	7:17244953:T:C	0.634	10.394	2.63E-25	0.0106	AHR
rs3735533	7:27206274:T:C	0.926	6.053	1.42E-09	0.0021	HOTTIP
rs17321515	8:125474167:A:G	0.476	-5.687	1.29E-08	0.0032	TRIB1,LINC00861
rs1801239	10:16877053:T:C	0.104	16.025	8.51E-58	0.0118	CUBN
rs45551835	10:16890385:G:A	0.014	20.385	2.28E-92	0.0226	CUBN
rs116867125	10:16991505:G:A	0.019	7.487	7.06E-14	0.0066	CUBN
rs10824368	10:76115357:G:A	0.226	6.246	4.22E-10	0.0041	
rs7115200	11:72041114:T:G	0.44	5.309	1.10E-07	0.0026	NUMA1
rs3201	12:69579612:T:C	0.345	-6.573	4.92E-11	0.0044	
rs4902647	14:68787474:C:T	0.464	-5.691	1.26E-08	0.0036	ZFP36L1,RNU6-921P
rs1288775	15:45369480:T:A	0.256	-5.95	2.68E-09	0.0034	GATM
rs2472297	15:74735539:C:T	0.266	9.642	5.31E-22	0.0068	CYP1A1,CYP1A2
rs12150031	17:81453198:C:G	0.391	-5.389	7.09E-08	0.0027	
rs547629	18:26627838:A:G	0.607	5.143	2.71E-07	0.0023	
rs613872	18:55543071:G:T	0.826	-5.517	3.44E-08	0.0029	TCF4
rs33950747	19:35848345:C:T	0.075	4.956	7.21E-07	0.002	
rs117287096	19:40835508:G:A	0.022	-5.042	4.61E-07	0.0021	CYP2A6,CYP2F2P
rs4021	19:48750004:A:G	0.278	-6.184	6.25E-10	0.0023	FUT1

Table S1: List of discovered conditionally-independent groups for Albuminuria GWAS. The mapped gene column corresponds to gene names provided by the NHGRI-EBI GWAS catalog. Variant = chromosome:position:ref-allele:alt-allele where position uses HG38 coordinates, AF = alternate allele frequency, Z = marginal Z score, W = Lasso coefficient difference statistic.

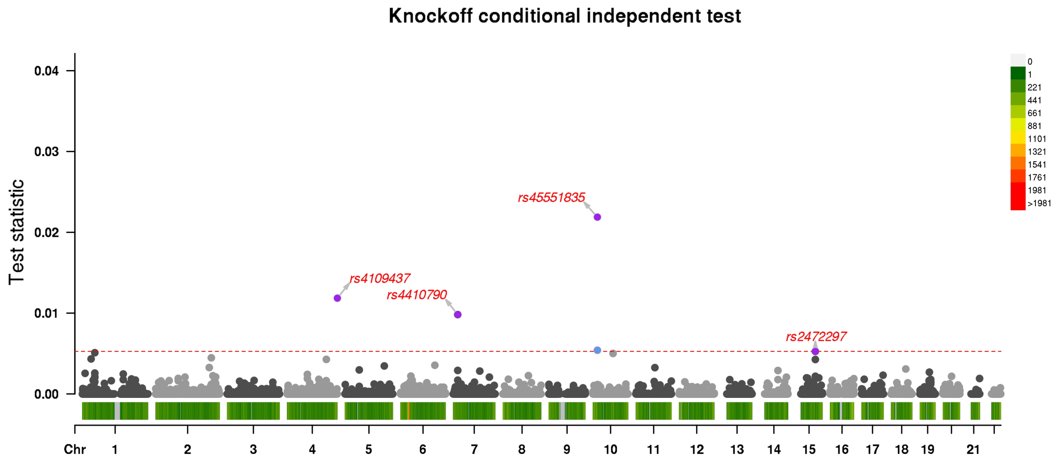

Figure S9: Albuminuria GWAS result using the tranditional eSDP algorithm for knockoff construction. Here the eSDP construction finds 4 significant SNPs after LD pruning. In contrast, ME group knockoffs featured in Figure S10 of the main text found 35 SNPs, which includes the 4 SNPs discovered by the eSDP method displayed here.

which is a gene that codes for the nephrin protein found in kidneys. It is known that this particular mutation leads to proteinuria (Deltas et al. 2023), but as far as we know, rs33950747 has not been previously reported. In fact, there are no marginally significant SNPs within a 1Mb window of NPHS1 in the original GWAS study. Variant rs7115200 is classified as a non-coding variant in the 5' untranslated region (5' UTR), which does not alter the amino acid sequence of a protein. All other variants fall within intronic and integenic regions and does not alter the amino acid sequence of a protein.

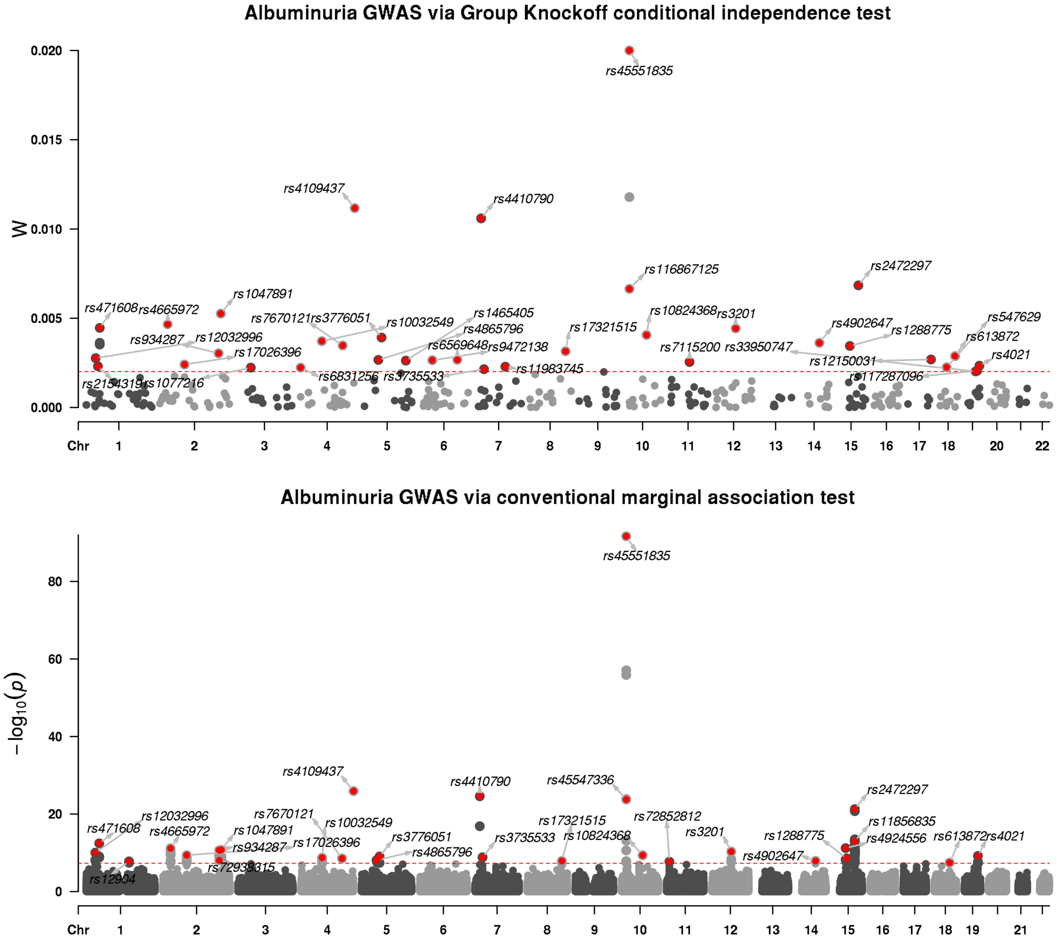

Figure S10: Summary statistics analysis of Albuminuria ($n = 382,500$ and $p = 630,017$) using group-knockoff-ghost-Lasso (top) compared to a conventional marginal-association testing approach (bottom). In the top panel, each dot represents a group, where the SNP with the most significant marginal p-value within the group is plotted. Only an independent discovery is highlighted in red. A table formatted result is available as Supplemental Table S1.

rsID	Gene name	Functional category
rs2154319	NcRNA intronic	
rs1077216	Intronic	
rs6831256	Intronic	
rs1465405	Intergenic	
rs6569648	Intronic	
rs9472138	Downstream	
rs11983745	Intronic	
rs7115200	NUMA1	non-coding
rs12150031	Intronic	
rs547629	Intronic	
rs33950747	NPHS1	Nonsynonymous SNV (Arg to Gln)
rs117287096	NcRNA intronic	

Table S2: Functional annotations for list of SNPs additionally discovered by ghost knockoff analysis in the Albuminuria GWAS. Gene name column identify whether variants cause protein coding changes using Gencode genes definition systems, and if so, lists the gene name that are impacted. The functional category column identifies the functional category of the variant, for which only the variant rs33950747 causes an amino acid sequence change.

References

[1] E. Anderson, Z. Bai, C. Bischof, S. Blackford, J. Demmel, J. Dongarra, J. Du Croz, A. Greenbaum, S. Hammarling, A. McKenney, and D. Sorensen. *LAPACK Users' Guide*. Third. Philadelphia, PA: Society for Industrial and Applied Mathematics, 1999.

[2] A. Askari, Q. Rebjock, A. d'Aspremont, and L. E. Ghaoui. "FANOK: Knockoffs in linear time". In: *SIAM Journal on Mathematics of Data Science* 3.3 (2021), pp. 833–853.

[3] T. Berisa and J. K. Pickrell. "Approximately independent linkage disequilibrium blocks in human populations". In: *Bioinformatics* 32.2 (2016), p. 283.

[4] R. Dai and R. Barber. "The knockoff filter for FDR control in group-sparse and multitask regression". In: *International conference on machine learning*. PMLR. 2016, pp. 1851–1859.

[5] C. Deltas, G. Papagregoriou, S. F. Louka, A. Malatras, F. Flinter, D. P. Gale, S. Gear, O. Gross, J. Hoefele, R. Lennon, et al. "Genetic modifiers of Mendelian monogenic collagen IV nephropathies in humans and mice". In: *Genes* 14.9 (2023), p. 1686.

[6] J. R. Gimenez and J. Zou. "Improving the stability of the knockoff procedure: Multiple simultaneous knockoffs and entropy maximization". In: *The 22nd International Conference on Artificial Intelligence and Statistics*. PMLR. 2019, pp. 2184–2192.

[7] Z. He, B. B. Chu, J. Yang, J. Gu, Z. Chen, L. Liu, T. Morrison, M. E. Belloy, X. Qi, G. Y. Le, S. Montgomery, H. M. Tang, T. M. Grecius Hastie, I. Ionita-laza, C. Sabatti, and E. Candes. "Beyond guilty by association at scale: searching for causal variants on the basis of genome-wide summary statistics". In: *BioRxiv* (2024), pp. 2024–02.

[8] O. Ledoit and M. Wolf. "Honey, I shrunk the sample covariance matrix". In: *UPF economics and business working paper* 691 (2003).

[9] J. Li and M. H. Maathuis. "GGM knockoff filter: False discovery rate control for Gaussian graphical models". In: *Journal of the Royal Statistical Society Series B: Statistical Methodology* 83.3 (2021), pp. 534–558.

[10] J. MacArthur, E. Bowler, M. Cerezo, L. Gil, P. Hall, E. Hastings, H. Junkins, A. McMahon, A. Milano, J. Morales, et al. "The new NHGRI-EBI Catalog of published genome-wide association studies (GWAS Catalog)". In: *Nucleic acids research* 45.D1 (2017), pp. D896–D901.

[11] T. S. H. Mak, R. M. Porsch, S. W. Choi, X. Zhou, and P. C. Sham. "Polygenic scores via penalized regression on summary statistics". In: *Genetic epidemiology* 41.6 (2017), pp. 469–480.

[12] Pan-UKB team. *Pan-UK Biobank*. https://pan.ukbb.broadinstitute.org. 2020.

[13] J. Schäfer and K. Strimmer. "A shrinkage approach to large-scale covariance matrix estimation and implications for functional genomics". In: *Statistical applications in genetics and molecular biology* 4.1 (2005).

[14] M. Sesia, S. Bates, E. Candès, J. Marchini, and C. Sabatti. "False discovery rate control in genome-wide association studies with population structure". In: *Proceedings of the National Academy of Sciences* 118.40 (2021), e2105841118.

[15] A. Sood and T. Hastie. "A Statistical View of Column Subset Selection". In: *arXiv preprint arXiv:2307.12892* (2023).

[16] A. Spector and L. Janson. "Powerful knockoffs via minimizing reconstructability". In: *The Annals of Statistics* 50.1 (2022), pp. 252–276.

[17] J. Yang and T. Hastie. *ghostbasil*. https://github.com/JamesYang007/ghostbasil. 2023.

[18] Q. Zhang, F. Privé, B. Vilhjálmsson, and D. Speed. "Improved genetic prediction of complex traits from individual-level data or summary statistics". In: *Nature communications* 12.1 (2021), p. 4192.

[19] H. Zhou, T. Arapoglou, X. Li, Z. Li, X. Zheng, J. Moore, A. Asok, S. Kumar, E. E. Blue, S. Buyske, et al. "FAVOR: functional annotation of variants online resource and annotator for variation across the human genome". In: *Nucleic acids research* 51.D1 (2023), pp. D1300–D1311.
